# Classification and prediction of cognitive performance differences in older age based on brain network patterns using a machine learning approach

**DOI:** 10.1162/netn_a_00275

**Published:** 2023-01-01

**Authors:** Camilla Krämer, Johanna Stumme, Lucas da Costa Campos, Christian Rubbert, Julian Caspers, Svenja Caspers, Christiane Jockwitz

**Affiliations:** Institute of Neuroscience and Medicine (INM-1), Research Centre Jülich, Jülich, Germany; Institute for Anatomy I, Medical Faculty & University Hospital Düsseldorf, Heinrich Heine University Düsseldorf, Düsseldorf, Germany; Department of Diagnostic and Interventional Radiology, Medical Faculty & University Hospital Düsseldorf, Heinrich Heine University Düsseldorf, Düsseldorf, Germany

**Keywords:** Cognition, Aging, Resting-state functional connectivity, Graph-theoretical analyses, Machine learning

## Abstract

Age-related cognitive decline varies greatly in healthy older adults, which may partly be explained by differences in the functional architecture of brain networks. Resting-state functional connectivity (RSFC) derived network parameters as widely used markers describing this architecture have even been successfully used to support diagnosis of neurodegenerative diseases. The current study aimed at examining whether these parameters may also be useful in classifying and predicting cognitive performance differences in the normally aging brain by using machine learning (ML). Classifiability and predictability of global and domain-specific cognitive performance differences from nodal and network-level RSFC strength measures were examined in healthy older adults from the 1000BRAINS study (age range: 55–85 years). ML performance was systematically evaluated across different analytic choices in a robust cross-validation scheme. Across these analyses, classification performance did not exceed 60% accuracy for global and domain-specific cognition. Prediction performance was equally low with high mean absolute errors (*MAE*s ≥ 0.75) and low to none explained variance (*R*^2^ ≤ 0.07) for different cognitive targets, feature sets, and pipeline configurations. Current results highlight limited potential of functional network parameters to serve as sole biomarker for cognitive aging and emphasize that predicting cognition from functional network patterns may be challenging.

## Author Summary

In recent years, new insights into brain network communication related to cognitive performance differences in older age have been gained. Simultaneously, an increasing number of studies has turned to machine learning (ML) approaches for the development of biomarkers in health and disease. Given the increasing aging population and the impact cognition has on the quality of life of older adults, automated markers for cognitive aging gain importance. This study addressed the classification and prediction power of resting-state functional connectivity (RSFC) strength measures for cognitive performance in healthy older adults using a battery of standard ML approaches. Classifiability and predictability of cognitive abilities was found to be low across analytic choices. Results emphasize limited potential of these metrics as sole biomarker for cognitive aging.

## INTRODUCTION

Healthy older adults vary greatly in the extent to which they experience age-related cognitive decline (Habib et al., 2007). While some older adults seem to maintain their cognitive abilities until old age, others show higher rates of cognitive decline during the aging process (Cabeza, 2001; Damoiseaux et al., 2008; Hedden & Gabrieli, 2004; Raz, 2000; Raz & Rodrigue, 2006). In light of the continuously growing aging population, the impact of cognitive decline on everyday functioning of older adults has gained momentum in research (Avery et al., 2020; Deary et al., 2009; Depp & Jeste, 2006; Fountain-Zaragoza et al., 2019; Luciano et al., 2009; Vieira et al., 2022).

In this context, differences in the functional architecture of brain networks have been identified as a potential source of variance explaining cognitive performance differences during aging (Chan et al., 2014; Stumme et al., 2020). Age-related differences have been linked to changes in resting-state functional connectivity (RSFC) of major resting-state networks, for example, the default mode network (DMN), the sensorimotor network (SMN), and the fronto-parietal and visual networks (Andrews-Hanna et al., 2007; Chong et al., 2019; Ng et al., 2016; Stumme et al., 2020). In detail, age-related cognitive decline is associated with both decreases in the functional specialization of brain networks (reduced network segregation) and increasingly shared coactivation patterns between functional brain networks (increased network integration) (Andrews-Hanna et al., 2007; Chan et al., 2014; Chong et al., 2019; Fjell et al., 2015; Grady et al., 2016; Ng et al., 2016; Onoda et al., 2012; Stumme et al., 2020). Furthermore, RSFC differences in older age may differentiate between healthy older adults and individuals suffering from mild cognitive impairment (MCI) or Alzheimer’s disease (AD). For instance, both MCI and AD have been related to reduced RSFC within the DMN and SMN, the degeneration of specific brain hubs, and aberrant functional brain network organization (Dai et al., 2015; Farahani et al., 2019; Sanz-Arigita et al., 2010; Supekar et al., 2008; Wang et al., 2013).

Given the role of RSFC network patterns in cognition in healthy and pathological aging, research on neurodegenerative diseases has started to embark on the development of diagnostic biomarker for automatic patient classification based on RSFC. For the development of diagnostic biomarkers, machine learning (ML) methods may be particularly suited. This is due to their ability to deal with high-dimensional data and to detect spatially distributed effects in the brain that might otherwise not be detected using univariate approaches (Dadi et al., 2019; Orrù et al., 2012; Woo et al., 2017; Zarogianni et al., 2013). In this context, RSFC-derived metrics capturing network integration and segregation have already been successfully used as diagnostic markers for MCI and AD, using ML approaches (Hojjati et al., 2017; Khazaee et al., 2016). In healthy older populations, functional network measures have also provided new insights into brain network communication related to cognitive performance differences (Chan et al., 2014; Chong et al., 2019; Stumme et al., 2020). Specifically, a previous study has demonstrated that shifts in within- and inter-network connectivity may be linked to differences in cognitive performance in older age (Stumme et al., 2020). Thus, RSFC network properties may also constitute potential meaningful candidates in search for a marker for nonpathological age-related cognitive decline (Chan et al., 2014; Stumme et al., 2020).

Previous studies have mainly used RSFC matrices, either containing information across the whole-brain or within specific networks, as input features to ML revealing initial promising results in the prediction of different cognitive facets in older adults (Avery et al., 2020; He et al., 2020; Kwak et al., 2021; Pläschke et al., 2020). For instance, it has been shown that working memory performance could be predicted by specific RSFC patterns in meta-analytically defined brain networks in an older but not younger age group by using relevance vector regression (RVR) (Pläschke et al., 2020). Furthermore, a variety of neuropsychological test scores and fluid intelligence could be successfully predicted from RSFC in large older samples using ML (He et al., 2020; Kwak et al., 2021). Nevertheless, it remains unclear if RSFC strength measures targeting network integration and segregation may provide additional useful information in classifying and predicting global and domain-specific cognitive performance in older adults (Avery et al., 2020; Dubois et al., 2018; He et al., 2020; Kwak et al., 2021; Pläschke et al., 2020). Further knowledge in this context may be helpful on the road to building a reliable and accurate biomarker for cognitive performance in healthy older adults that could ultimately be used to predict prospective cognitive decline. The current investigation, therefore, aims to systematically examine whether RSFC strength parameters, capturing within- and inter-network connectivity, may reliably classify and predict cognitive performance differences in a large sample of older adults (age: 55–85) from the 1000BRAINS study by using a battery of standard ML approaches.

## MATERIALS AND METHODS

### Participants

**Figure 1.**
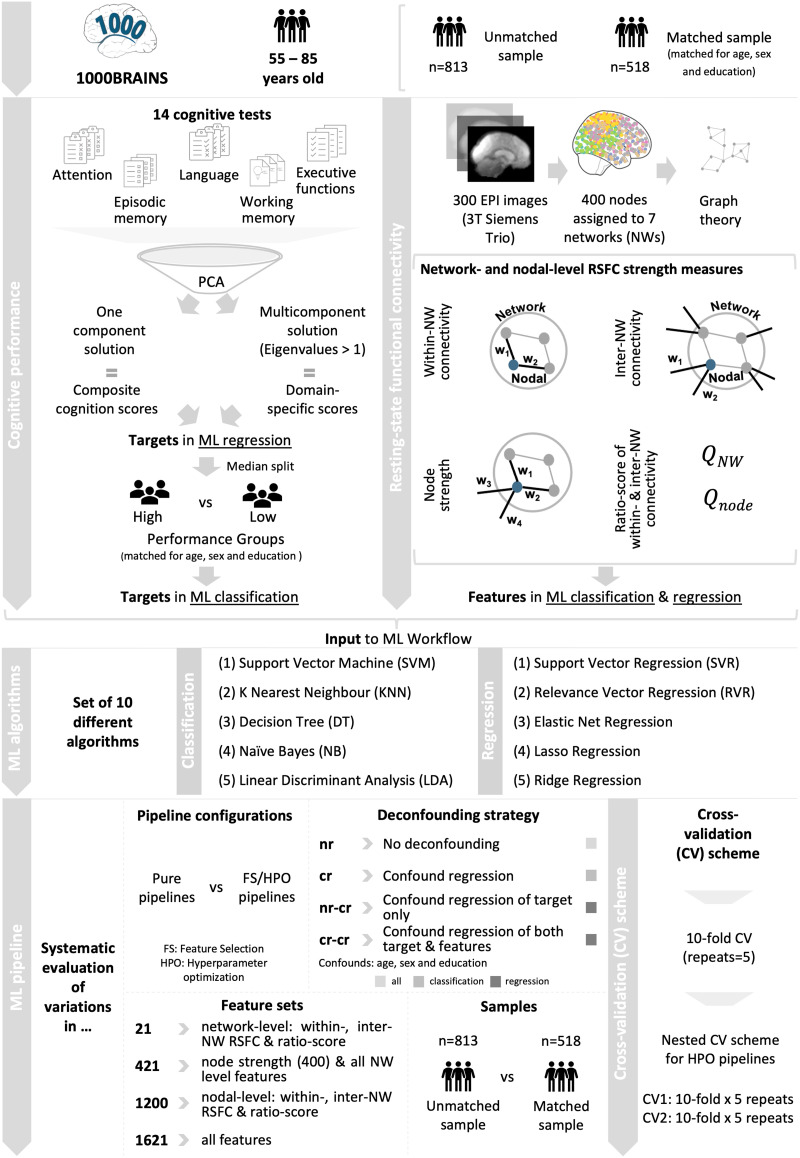
Schematic overview of workflow.

Data for the current investigation stems from the 1000BRAINS project (Caspers et al., 2014), an epidemiologic population-based study examining variability of brain structure and function during aging in relation to behavioral, environmental, and genetic factors. The 1000BRAINS sample was drawn from the 10-year follow-up cohort of the Heinz Nixdorf Recall Study and the associated MultiGeneration study (Schmermund et al., 2002). As 1000BRAINS aims at the characterization of the aging process in the general population, no exclusion criteria other than eligibility for MR measurements (Caspers et al., 2014) were applied. In the current study, 966 participants were included within the age range 55 to 85 years. From this initial sample, 99 participants were excluded due to missing resting-state functional magnetic resonance imaging (fMRI) data or failed preprocessing. Furthermore, 25 participants were excluded due to insufficient quality of the preprocessed functional data described in further detail below (see Data Acquisition and Preprocessing section). Another 27 participants with missing scores on the DemTect, a dementia screening test, or those scoring smaller or equal to 8 were excluded due to the possibility of substantial cognitive impairment (Kalbe et al., 2004). Finally, two participants were excluded due to more than three missing values within the neuropsychological assessment (see Cognitive Performance section). This resulted in an initial (unmatched) sample of 813 participants (372 females, *M*_age_ = 66.99, *SD*_age_ = 6.70; see Table 1A and Figure 1: Sample). All subjects provided written consent prior to inclusion and the study protocol of 1000BRAINS was approved by the Ethics Committee of the University of Essen, Germany.

**Table 1.** Demographic information for unmatched and matched samples regarding age, educational level, and risk of dementia

	A. Unmatched sample				B. Matched sample			
	*N*	Age	Education	DemTect	*N*	Age	Education	DemTect
Female	372	66.38 (6.53)	5.93 (1.84)	15.42 (2.29)	232	65.33 (5.48)	5.88 (1.7)	15.43 (2.22)
Male	441	67.5 (6.8)	6.95 (1.91)	14.38 (2.33)	286	67.81 (6.44)	6.96 (1.87)	14.45 (2.25)
Total	813	66.99 (6.70)	6.48 (1.94)	14.86 (2.37)	518	66.7 (6.15)	6.48 (1.87)	14.89 (2.29)

*Note*. Mean displayed with standard deviation (*SD*) appearing in parentheses.

### Cognitive Performance

All subjects underwent a large neuropsychological assessment testing the cognitive domains attention, executive functions, episodic memory, working memory (WM), and language (for further details, see Caspers et al., 2014). Fourteen cognitive variables targeting selective attention, processing speed, figural and verbal fluency, problem solving, vocabulary, WM, and episodic memory were selected for the purpose of the current study (see Figure 1: Cognitive performance). Further information on the tests and variables chosen in the current investigation are found in Supporting Information Table S1. In case of missing values (more than three missing values led to exclusion) in the neuropsychological assessment, missing values were replaced by the median for respective sex (males, females) and age groups (55–64 years, 65–74 years, 75–85 years). Imputation of missing values was performed to avoid further loss of information and power. In a next step, raw scores from all 14 neuropsychological tests used in the analysis were transformed into *z*-scores. For interpretability purposes, scores for neuropsychological tests with higher values meaning lower performance (i.e., time to complete the tasks or number of errors made) were inverted.

Neuropsychological test performance was reduced to cognitive composite scores using principal component analysis (PCA). To disentangle effects specific to certain cognitive facets, global and domain-specific cognitive performance were examined (Tucker-Drob, 2011). PCA was used to extract a one-component solution for global cognition and a multicomponent solution for cognitive subdomains based on eigenvalues >1. Lastly, varimax rotation was applied to enhance the interpretability of extracted components. Individual global and domain-specific component scores obtained from the PCA were used as targets in ML prediction of cognitive performance differences.

For classification of cognitive performance differences, the initial (unmatched) sample was separated into high- and low-performing groups. To do so, a median split was performed based on each of the three cognitive component scores (as extracted in the PCA). To remove the effect of potential confounders, the high- and low-performance groups derived from global cognition were additionally matched with respect to age, sex, and educational level by using propensity score matching, which constitutes a statistical approach to match participants based on their propensity scores (McDermott et al., 2016; Randolph et al., 2014; Stern et al., 1994; Vemuri et al., 2014). This led to a matched sample with *N* = 518 (232 females, *M*_age_ = 66.7, *SD*_age_ = 6.15; see Table 1B and Figure 1: Sample and Cognitive performance). Further demographic information regarding age, educational level, and sex distribution between high- and low-performance groups in the unmatched and matched sample can be found in Table 2. All cognitive analyses were performed using IBM SPSS Statistics 26 (https://www.ibm.com/de-de/analytics/spss-statistics-software) and customized Python (Version 3.7.6) and R scripts (Version 4.00).

**Table 2.** Differences in cognitive scores, age, educational level, and sex distribution between high- and low-performance groups in the unmatched and matched sample

		COGNITIVE COMPOSITE					NON-VERBAL MEMORY & EXECUTIVE					VERBAL MEMORY & LANGUAGE				
		Group					Group					Group				
		Low	High	t	p	df	Low	High	t	p	df	Low	High	t	p	df
Unmatched Sample	Cog. Score	−.79 (0.72)	.79 (0.47)	−37.17	<0.001	697.9	−.78 (0.68)	.78 (0.56)	−36.02	<0.001	784.8	−.81 (0.60)	.80 (0.59)	−36.67	<0.001	811
	Age	69.49 (6.43)	64.49 (5.99)	11.48	<0.001	811	69.24 (6.58)	64.72 (6.02)	10.28	<0.001	805.1	68.09 (6.72)	65.89 (6.5)	4.74	<0.001	811
	Education	5.84 (1.76)	7.13 (1.91)	−10.51	<0.001	805.0	6.03 (1.88)	6.94 (1.9)	−6.87	<0.001	810.8	5.97 (1.76)	7.00 (1.99)	−7.81	<0.001	800
	Males	206	235	–	–	–	187	254	–	–	–	245	196	–	–	–
	Females	200	172	–	–	–	220	152	–	–	–	161	211	–	–	–

Matched Sample	Cog. Score	−.66 (0.63)	.71 (0.44)	−28.67	<0.001	460.2	−.68 (0.61)	.75 (0.54)	−28.35	<0.001	516	−.74 (0.54)	.74 (0.53)	−31.24	<0.001	516
	Age	67.06 (6.1)	66.34 (6.2)	1.32	0.19	516	67.69 (6.20)	65.74 (5.95)	3.65	<0.001	516	66.63 (6.01)	66.77 (6.29)	−.25	.81	516
	Education	6.39 (1.82)	6.56 (1.92)	−.1.06	0.29	516	6.31 (1.85)	6.64 (1.88)	−2.01	<0.05	516	6.3 (1.77)	6.67 (1.96)	−2.25	<0.05	506.1
	Males	143	143	–	–	–	127	159	–	–	–	165	121	–	–	–
	Females	116	116	–	–	–	128	104	–	–	–	99	133	–	–	–

*Note*. Standard deviation (*SD*) appears in parentheses. Cog. Score = cognitive score. Unmatched sample: global: *X*^2^(1) = 4.01, *p* < .05; memory and executive: *X*^2^(1) = 22.61, *p* < .001; language: *X*^2^(1) = 12.16, *p* < .001; Matched Sample: global: *X*^2^(1) = 0, *p* = 1; memory and executive: *X*^2^(1) = 5.94, *p* < .05; language: *X*^2^(1) = 11.56, *p* < .001.

### Functional Imaging

#### Data acquisition and preprocessing.

Imaging data was acquired using a 3T Siemens Tim-TRIO MR scanner with a 32-channel head coil. Out of the whole MR imaging protocol (for details, see Caspers et al. 2014), the current study used for surface reconstruction the 3D high-resolution T1-weighted magnetization-prepared rapid acquisition gradient-echo (MPRAGE) (176 slices, slice thickness = 1 mm, TR = 2,250 ms, TE = 3.03 ms, FoV = 256 × 256 mm^2^, flip angle = 9°, voxel resolution = 1 × 1 × 1 mm^3^); and for resting-state analyses, the 11:30 minutes resting-state fMRI with 300 EPI (gradient-echo planar imaging) volumes (slices 36, slice thickness = 3.1 mm, TR = 2,200 msec, TE = 30 msec, FoV = 200 × 200 mm^2^, voxel resolution = 3.1 × 3.1 × 3.1 mm^3^). During the resting-state scan, participants were instructed to keep their eyes closed, to relax and let their mind wander, but not to fall asleep. This was checked during a postscan debriefing.

Preprocessing steps closely followed those from Stumme and colleagues (2020). During preprocessing, the first four volumes from the 300 EPI were removed for each participant. All functional images were corrected for head movement using a two-pass procedure. First, all volumes were aligned to the first image and then to the mean image using affine registration. Spatial normalization to the MNI152 template (2-mm-voxel size) of all functional images was achieved by using a “unified segmentation” approach as previous studies have shown increased registration accuracies compared to normalization based on T1-weighted images (Ashburner & Friston, 2005; Calhoun et al., 2017; Dohmatob et al., 2018). Furthermore, ICA-AROMA, that is, ICA-based automatic removal of motion artifacts (Pruim et al., 2015), which constitutes a data-driven method for the identification and removal of motion-related components from MRI data, was applied. Additionally, global signal regression (GSR) was performed in order to minimize the association between motion and RSFC (Burgess et al., 2016; Ciric et al., 2017; Parkes et al., 2018). Moreover, GSR has been found to improve behavioral prediction performance and to enhance the link between RSFC and behavior (Li et al., 2019). In a final step, a band-pass filter was applied (0.01–0.1 Hz). As a quality check for our preprocessing, further steps were implemented. Initially, we checked for potential misalignments in the mean functional AROMA data with the check sample homogeneity option in the Computational Anatomy Toolbox (CAT 12) (Gaser et al., 2022). Participants detected as outliers with >2 *SD* away from the mean were excluded. Additionally, we checked for volume-wise severe intensity dropouts (DVARS) in the preprocessed data by using an algorithm by Afyouni and Nichols (2018). For each participant, *p* values for spikes are generated, and participants with more than 10% of the 300 volumes detected as dropouts were excluded from further analyses. To check the quality control applied, we assessed the correlation between age and motion after the application of AROMA and the exclusion of deviating participants and found it to be nonsignificant (percentage (%) of corrupted volumes * age, *r* = .03, *p* = .39).

#### Functional connectivity analyses.

For connectivity analyses, the 400-node cortical parcellation by Schaefer and colleagues (2018) was adopted. The 400 regions of interest from the parcellation scheme can be allocated to seven network parcels of known functional resting-state networks (Yeo et al., 2011). These include the visual, sensorimotor, limbic, fronto-parietal, default mode, dorsal, and ventral attention network.

A whole-brain graph was established from functional data (Rubinov & Sporns, 2010). This included, (i) a mean time series extraction for each node using fslmeants (Smith et al., 2004), (ii) individual edge definition as the Pearson’s correlation of respective average time series of two nodes, (iii) a statistical significance test of each correlation coefficient using the Fourier transform and permutation testing (repeats = 1,000) with nonsignificant edges at *p* ≥ 0.05 being set to zero (Stumme et al., 2020; Zalesky et al., 2012), and (iv) Fisher’s *r*-to-*z*-transformation applied to the 400 × 400 adjacency matrix. Furthermore, since there is still debate about the true nature of anticorrelations in the brain, only positive correlations were considered in subsequent analyses (negative correlations were set to zero) (Murphy et al., 2009; Murphy & Fox, 2017; Saad et al., 2012). Finally, no further thresholding related to network density or network size was applied to the brain graph as it may, in addition to controlling the absolute number of edges, also increase the number of false positives and induce systematic differences in overall RSFC (Stumme et al., 2020; van den Heuvel et al., 2017; van Wijk et al., 2010). For the estimation of strength measures, the final network used, thus, may be described as a positively weighted network.

In a next step, connectivity estimates were calculated using the software bctpy with network parameters defined as in Rubinov and Sporns (2010) (https://pypi.org/project/bctpy/). All metrics estimated in the current investigation are based on the estimation of strength values, which do not appear to be distorted by varying amounts of edges and have been shown to reliably quantify networks (Finn et al., 2015). In total, seven parameters were computed for later use in ML. Within- and inter-network RSFC as well as a ratio-score indicating network segregation were obtained at both network and nodal level (see Figure 1: RSFC; for further details on network parameters, see Stumme et al., 2020). Within-network RSFC was defined as the sum of strength values from all nodes (network) or one node (nodal) within a network to all nodes within its related network divided by the number of existing edges in the network (network: 7 features; nodal: 400 features). Inter-network RSFC referred to the sum of strength values from all nodes (network) or one node (nodal) within a network to all nodes outside its network divided by the number of all edges in the network (network: 7 features; nodal: 400 features). The ratio-score captured within-network RSFC of all nodes (network) or one node (nodal) in relation to its inter-network RSFC (network: 7 features; nodal: 400 features). Additionally, the strength of each node was calculated as the sum of all connectivity weights attached to a node (i.e., 400 features). In total, the feature vector for each subject consisted of 1,621 features (4 × 400 = 1,600 nodal features and 3 × 7 = 21 network-level features). From this, four different feature sets were derived and used in ML (21 features: all network-level features; 421 features: node strength and all network-level features; 1,200 features: nodal within- and inter-network and ratio of within/inter-network RSFC; 1,621 features: all features).

### Systematic Application of a Battery of Standard Machine Learning Approaches

ML was used to assess whether RSFC strength measures can be used to distinguish (i.e., classification) and predict (i.e., regression) cognitive performance differences in older adults. As there is currently no agreement on a standard ML pipeline using neuroimaging data given the high variability in dataset properties, we systematically evaluated different analytical choices (see Figure 1: ML algorithms and pipeline). Performance of different ML algorithms, pipeline compositions, extents of deconfounding, and variations in feature set and sample sizes were assessed (Arbabshirani et al., 2017; Cui & Gong, 2018; Khazaee et al., 2016; Mwangi et al., 2014; Paulus & Thompson, 2021; Pervaiz et al., 2020). As such, we tested a total of 556 unique pipelines in the classification (406 pipelines) and regression (150 pipelines) setting. The scikit-learn library (version: 0.22.1) in Python (Version 3.7.6) (Pedregosa et al., 2011; https://scikit-learn.org/stable/index.html) was used for all ML analyses unless specified.

#### ML algorithms.

For classification, Five different algorithms were examined: support vector machine (SVM), K-nearest while (KNN), decision tree (DT), naïve Bayes (NB) and linear discriminant analysis (LDA). Further information on the algorithms can be found in the Supporting Information Methods.

For regression, five different algorithms were assessed: support vector regression (SVR), RVR, Ridge regression (Ridge), least absolute shrinkage and selection operator regression (LASSO), and elastic net regression (Elastic Net) (Cui & Gong, 2018). The package scikit-rvm compatible with scikit-learn by James Ritchie (https://github.com/JamesRitchie/scikit-rvm) was used for RVR computation. Further information on the regression algorithms can be found in the Supporting Information Methods.

#### Basic ML pipeline.

The basic ML pipeline was constructed as follows: the previously calculated connectivity estimates were used as input features for the ML workflow. Targets varied between classification (high vs. low cognitive performance group; matched sample) and regression (global and domain-specific cognitive scores; unmatched sample) (see Cognitive Performance section in Materials and Methods). Input features were scaled to unit variance in a first step in all pipeline configurations within the cross-validation setting. All models were evaluated using a repeated 10-fold cross-validation (CV) (five repeats). In case of an additional hyperparameter optimization (HPO) step, a repeated nested CV scheme was implemented for selecting optimal parameters (outer and inner loop: 10 folds × 5 repeats) (see Figure 1: CV scheme; Lemm et al., 2011). This was done to avoid data leakage and to obtain an unbiased estimate of the generalization performance of complete models (Lemm et al., 2011). Balanced accuracy (BAC) was used to assess classification performance. It was chosen to account for potential group size differences in domain-specific cognition. Sensitivity and specificity were also calculated to provide a more complete picture and can be found in the Supporting Information. Mean absolute error (*MAE*) and coefficient of determination (*R*^2^) were computed in the prediction setting.

#### Systematic evaluation of ML pipeline options.

Regarding pipeline configurations, different pipeline configurations were investigated. Performance of baseline models were compared to those from pipelines with feature selection (FS) and HPO as they have been found to greatly impact ML performance (Brown & Hamarneh, 2016; Guyon & Elisseeff, 2003; Hua et al., 2009; Mwangi et al., 2014). For baseline models, algorithms were run with default settings from scikit-learn without additional FS and HPO steps (pure pipeline). If FS was not performed in conjunction with HPO, default parameters were equally used. We investigated different FS methods in the present study (Mwangi et al., 2014).

For classification, two univariate filters, that is, ANOVA *F*-test and mutual information, were compared to L1-based (using a linear SVM) and hybrid FS. For the univariate filters, the top 10% of features were selected. Furthermore, L1-based (i.e., regularization) FS using a linear SVM to create sparse models in combination with the five classifiers was examined. Finally, a hybrid FS method, which combines both filter and wrapper methods, was considered (Kazeminejad & Sotero, 2019; Khazaee et al., 2016). Initially, a univariate filter (ANOVA *F*-test) was applied selecting 50% of the top performing features. On the remaining half of the features, a sequential forward floating selection wrapper was used to determine the top 10 features contributing to the classification using the mlxtend package for Python (Khazaee et al., 2016; Pudil et al., 1994; Raschka, 2018). FS was always performed on the training set.

Different FS methods were also examined in ML regression. A univariate correlation–based filter was applied in case of SVR, RVR, and Ridge regression (Finn et al., 2015; Guyon & Elisseeff, 2003). Again the top 10% of features were selected. In contrast, LASSO and Elastic Net regression are embedded FS algorithms. Due to their regularization penalty, only features with a high discriminatory power will have a nonzero weight and will contribute to the task at hand (Zou & Hastie, 2005). Thus, they enforce sparsity and with it integrate FS in their optimization problem (Mwangi et al., 2014).

In terms of HPO, three of the five classification algorithms had hyperparameters to be tuned, that is, SVM, KNN, and DT. HPO was carried out for (i) regularization parameter C for SVM (10^−4^ to 10^1^, 10 steps, logarithmic scale) for linear, radial basis function (RBF) and polynomial (poly) kernel, (ii) maximum tree depth (4, 6, 8, 10, 20, 40, None) and optimum criterion (gini impurity vs entropy) for DT, and (iii) number of neighbors for KNN (1, 3, 5, 7, 9, 11,13, 15, 17, 19, 21, 23, 25). HPO was assessed with and without additional FS (ANOVA *F*-test) in classification. The following hyperparameters were tuned in ML prediction: (i) regularization parameter lambda *λ* for LASSO and Ridge regression (LASSO: 10^−1^ to 10^2^, Ridge: 10^−3^ to 10^5^, 10 steps, logarithmic scale); (ii) parameters lambda, *λ*, and alpha, *α*, for Elastic Net (*λ* : 10^−1^ to 10^2^, 10 steps, logarithmic scale; *α*: 0 to 1, 10 steps); and (iii) regularization parameter C for SVR (10^−4^ to 10^1^, 10 steps, logarithmic scale) and kernel type (linear, RBF, and poly). HPO was assessed in conjunction with FS in prediction as some algorithms incorporated embedded feature selection. All HPO was performed on the inner loop using grid search assessing the performance of all parameter combinations and choosing the best one in terms of inner loop performance. All pipeline options were explored for feature sets without (nr condition) and with deconfounding (cr, nr-cr, cr-cr condition) applied.

For deconfounding strategy, if deconfounding was applied, the covariates age, sex and educational level were regressed from features/targets. To avoid data leakage, confound regression was always carried out within the ML pipeline. Following Rasero and colleagues (2021), confounders were regressed from targets/features by using a linear regression model, which was fit using only the training set and then applied to both training and test data to obtain residuals. Different extents of deconfounding (nr = no deconfounding; classification: cr = confounders regressed from features; regression: nr-cr = confounders regressed from targets, cr-cr = confounders regressed from both features and targets) were implemented to assess its impact on ML performance (Pervaiz et al., 2020).

For ML validation analyses, we performed several further analyses to validate our ML approach. First, we investigated the influence of a finer grained parcellation on ML performance (Dadi et al., 2019; Khazaee et al., 2016). Therefore, we compared ML performance results obtained from using a 400-node and 800-node parcellation (Schaefer et al., 2018). Additionally, ML performance was explored separately in males and females, given the well-established gender differences in RSFC and its potential impact on ML performance (Nostro et al., 2018; Stumme et al., 2020; Weis et al., 2019). Furthermore, we examined whether the inclusion of information from negative correlations in terms of functional connectivity may alter ML performance results. In this context, we calculated our strength measures based on (i) the absolute values from both positive and negative correlations and (ii) only on the absolute values from negative correlations and used these separately as features to ML. Additionally, we investigated how classification performance changes when only extreme groups, defined as the highest and lowest 25% of individuals scoring on the global cognition component, are included (Dadi et al., 2021; Vieira et al., 2022). Classification performance was examined in unmatched and matched (for age, sex, and education) samples (see Supporting Information Tables S2–S3). In terms of validating our pipeline, we tested our ML pipelines in the context of age, which has repeatedly been shown to be successfully predicted from RSFC patterns (Liem et al., 2017; Meier et al., 2012; Pläschke et al., 2017; Vergun et al., 2013). To adapt this to our classification setting, we examined the classification of extreme age groups (old vs. young; see Supporting Information Tables S4–S5) in feature set 421 (Vieira et al., 2022). In the prediction setting, age was predicted continuously. Prediction analyses were carried out for extreme groups, the unmatched sample and the whole age range of the 1000BRAINS cohort (18–85 age) (see Supporting Information Tables S4–S5).

### Model Comparisons and Statistical Analyses

To assess the reliability and stability of the derived principal components (PCs), we performed two additional analyses. First, we checked for the robustness of the PCA against the imputation of missing values on different cognitive tests. Therefore, we obtained a validation sample, in which all participants with missing values in any of the cognitive tests were excluded from the unmatched sample (*N* = 749, 343 females, *M*_age_ = 66.86, *SD*_age_ = 6.62). Then, we compared component loadings from the original PCA results to the recalculated ones in the validation sample by calculating Pearson’s correlations. Second, we turned to the stability of the PCs across data splits to address the dependency between training and test sets introduced by performing PCA as a first step in the analysis outside of the ML framework. In case of stability of PCs, we may assume that this dependency will not affect our results. Therefore, we additionally divided the data into two subsamples (random split-half procedure was implemented; Sripada et al., 2020b; Thompson et al., 2019) and performed a PCA on each sample separately. Component loadings from the split halves were compared to the original loadings by computing Pearson’s correlations (see Supporting Information Tables S9–S10).

To assess the relation between cognitive scores derived from PCA and potential confounding factors, we calculated partial correlations between all cognitive scores (global and domain specific) and age (corrected for education and sex) as well as education (corrected for age and sex) in the unmatched sample. Furthermore, to examine sex differences in cognitive scores, a multivariate analysis of covariance (MANCOVA) was computed with cognitive scores as dependent variables, sex as the independent variable, and the inclusion of age and education as covariates.

For checking the quality of the dichotomization into a high- and low-performance group, we performed independent samples *t*-tests to test for significant differences in cognitive performance (global and domain specific) between high- and low-performance groups in the unmatched and matched sample. Additionally, we assessed the relation between confounding factors and group membership. Thus, we performed independent samples *t*-test to examine group differences in terms of age and education and chi-square tests for independence to assess differences in the sex distribution across high- and low-performance groups in unmatched and matched samples.

To contextualize ML performance and obtain a chance-level prediction equivalent, we compared ML model estimations to those from a reference model, that is, dummy classifier and regressor, given the low computational costs of dummy estimates and their similarity in distribution to approaches based on permutation (Engemann et al., 2020; Vieira et al., 2022). In this case, the percentage of folds, for which the ML models were better than the reference model in terms of accuracy (classification) and *R*^2^ (regression), was calculated with higher percentages (>80%) indicating robust outperformance of the reference model.

## RESULTS

We performed twofold analyses to investigate whether cognitive performance differences could be distinguished and predicted based on RSFC strength measures. In a first step, a simple classification setting was chosen to examine if high- and low-performance groups can be accurately classified from RSFC strength parameters using different ML pipeline configurations, analytic choices, and feature sets. In a second step, we sought to address if the continuous prediction of cognitive scores leads to ML performance differences compared to the classification. Thus, we implemented a regression framework to analyze, whether cognitive performance differences could be predicted from RSFC strength measures.

### Cognitive Performance Across Unmatched and Matched Samples

**Figure 2.**
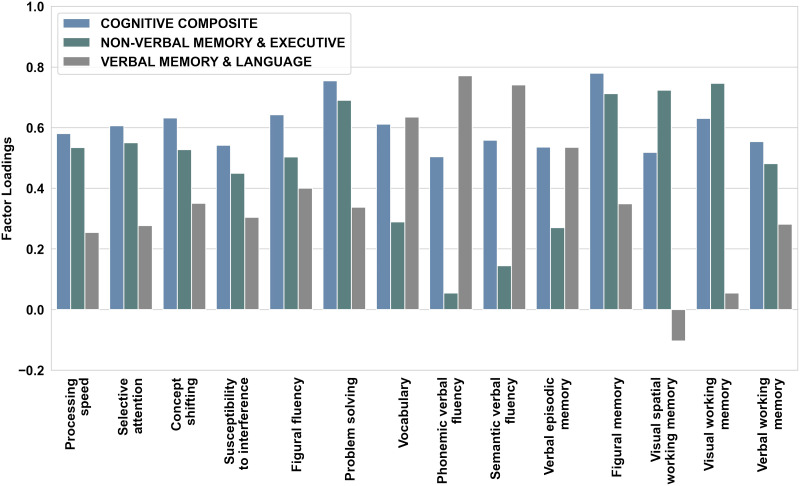
Factor loadings of each cognitive function on the one-component and multicomponent solution extracted from PCA analysis (after varimax rotation).

A one-component solution for global cognition and a multicomponent solution for cognitive subdomains based on the eigenvalue criterion (eigenvalue > 1) were extracted. Data suitability for PCA was tested with the Kaiser–Meyer–Olkin (KMO) index examining the extent of common variability. With a value of KMO = 0.91, data appeared suitable for PCA. Component scores from the one-component solution were stored as the COGNITIVE COMPOSITE (i.e., global cognition) score for each individual (see Figure 2 and Supporting Information Tables S6 and S7 and Figure S8). With regards to domain-specific cognitive scores, two components could be discovered from the PCA (see Figure 2 and Supporting Information Tables S6 and S7). The first component mainly covered performance in visual spatial and spatial WM, figural memory, problem solving, selective attention, and processing speed (NON-VERBAL MEMORY & EXECUTIVE component; see Figure 2 and Supporting Information Table S7). The second component centrally reflected performance on semantic and phonemic verbal fluency, vocabulary, and verbal episodic memory (VERBAL MEMORY & LANGUAGE component; see Figure 2 and Supporting Information Table S7). In terms of robustness and stability of PCs, component loadings for all three extracted components were highly similar across the original sample, the random split half samples and the validation sample (*r* > 0.86, *p* > 0.01; Supporting Information Tables S9 and S10) indicating that PCs appear stable across subsets of data and robust against the imputation of missing values. Age was significantly negatively correlated with global and domain-specific cognitive performance scores (controlled for sex and educational level; COGNITIVE COMPOSITE: *r* = −.48, *p* < .001; NON-VERBAL MEMORY & EXECUTIVE: *r* = −.43, *p* < .001; VERBAL MEMORY & LANGUAGE: *r* = −.19, *p* < .001). Higher educational level was significantly associated with higher global and domain-specific cognitive performance (COGNITIVE COMPOSITE: *r* = .40, *p* < .001; NON-VERBAL MEMORY & EXECUTIVE: *r* = .21, *p* < .001; VERBAL MEMORY & LANGUAGE: *r* = .35, *p* < .001; controlled for age and sex). A multivariate analysis of covariance (MANCOVA) with age and education as covariates revealed males to perform significantly better than females on the NON-VERBAL MEMORY & EXECUTIVE component (*F*(1, 809) = 30.22, *p* < .001, η_p_^2^ = 0.036), while females outperformed males on the VERBAL MEMORY & LANGUAGE component (*F*(1, 809) = 46.11, *p* < .001, η_p_^2^ = 0.056). In turn, no sex differences were found for global cognition (COGNITIVE COMPOSITE: *F*(1, 809) = 0.024, *p* = .877, η_p_^2^ = 0.0). Component scores (global and domain-specific) obtained from PCA were used as targets in ML prediction.

For classification of cognitive performance differences, high- and low-performance groups were created by a median split after the extraction of participants’ component scores (as extracted in the PCA). High- and low-performance groups in the initial (unmatched) sample differed significantly in global and domain-specific cognitive performance, as well as in terms of age, educational level, and sex (see Table 2). The high-performing group was found to be significantly younger and better educated than the low-performing group (see Table 2). More males than females were represented in the high-performance group for the COGNITIVE COMPOSITE and the NON-VERBAL MEMORY & EXECUTIVE component (see Table 2). The reversed pattern was found for the VERBAL MEMORY & LANGUAGE component (see Table 2).

To control for the impact of confounding factors, high- and low-performance groups of the COGNITIVE COMPOSITE component were matched on age, educational level, and sex. This led to a matched subsample (*N* = 518; see Figure 1: Sample and Table 1B). High- and low-performance groups again differed significantly in their global and domain-specific cognitive performance (see Table 2). No significant group differences were encountered in terms of age, educational level and sex distribution for the COGNITIVE COMPOSITE component (see Table 2). Participants in the low-performance group on the NON-VERBAL MEMORY & EXECUTIVE and VERBAL MEMORY & LANGUAGE component were found to be significantly less educated than participants in the high-performance group. A similar significant pattern for differences in the sex distribution was encountered as in the unmatched sample (see Table 2). Group memberships (high vs. low) were used as targets in ML classification.

### Classification Results

#### Classification performance across global cognition and cognitive domains.

**Figure 3.**
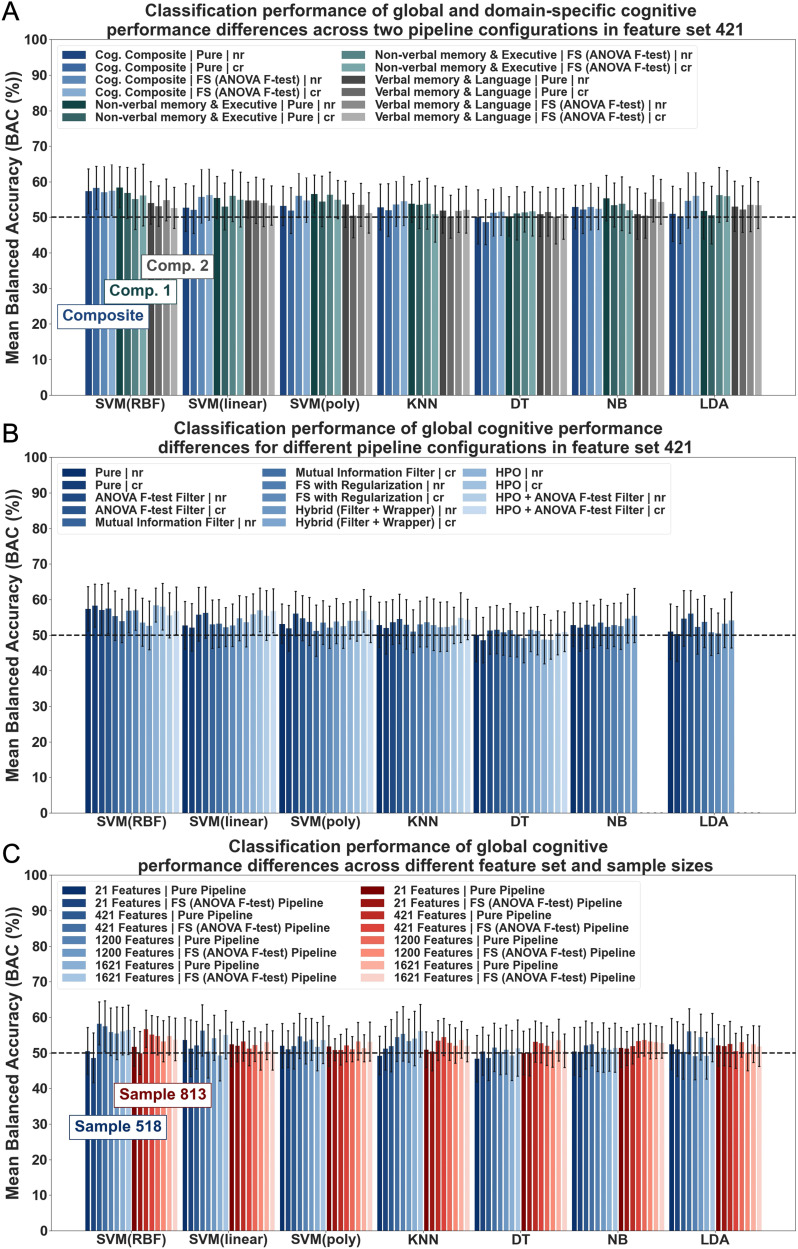
Classification performance results of cognitive performance differences (based on global and domain-specific scores) from RSFC strength measures. Classification results across algorithms: Support Vector Machine (SVM) with Radial Basis Function (RBF), linear and polynomial (poly) kernel, K-Nearest Neighbour (KNN), Decision Tree (DT), Naïve Bayes (NB), Linear Discriminant Analysis (LDA). Results shown for (A) different targets (cognitive composite and cognitive components), (B) pipeline configurations (pure (no FS/HPO) vs. FS/HPO pipelines), (C) samples (matched vs. unmatched sample) and feature set sizes (21, 421, 1,200, 1,621). Error bars correspond to standard deviation (*SD*); nr = no confound regression applied to features; cr = age, sex, and education regressed from features; unless otherwise specified, cr condition showed.

ML was used in a first step to assess the usefulness of RSFC strength measures to distinguish cognitive performance differences in older adults. All algorithms were first implemented in a feature set with 421 features to examine classification performance of global and domain-specific cognitive performance differences in the matched sample. Across all implemented ML pipelines with and without univariate feature selection (FS), performance did not exceed 60% accuracy (see Figure 3A and Supporting Information Table S11). Mean BACs ranged between 48.68% to 58.33% for global cognition and 50.21% to 58.44% for domain-specific cognition. These results were further supported by the comparison to the dummy classifier. The majority of models did not outperform the dummy classifier in more than 80% of folds. Higher accuracies compared to the dummy were achieved mainly in no more than 50% to 80% of folds, suggesting rather modest overall performance and limitations in reliability (see Supporting Information Table S12). Classification accuracies for the NON-VERBAL MEMORY & EXECUTIVE component were marginally higher than for the VERBAL MEMORY & LANGUAGE component, which was also supported by results from comparisons to the dummy estimate (see Figure 3A and Supporting Information Tables S11–S13). No systematic differences between models based on features with (cr) or without (nr) deconfounding, that is, controlling for the effects of age, sex, and education on features, could be observed (Figure 3A). Initial results suggested poor discriminatory power of RSFC strength measures for global and domain-specific cognitive performance differences in a large population-based older sample.

#### Classification performance across different pipeline configurations for global cognition.

To examine the impact of different pipeline configurations, we investigated ML performance in a pure pipeline, that is, without FS, and in FS/hyperparameter optimization (HPO) pipelines, that is, additional step of feature selection (FS) and HPO, for global cognition. All algorithms were first implemented in a pure pipeline using 421 features. Baseline results revealed classification accuracies between 48.68% to 58.33% (see Figure 3B). Baseline results were then compared to those from different FS/HPO pipelines. Estimations from FS/HPO pipelines were found to be similar to baseline estimations (M_BAC_ range: 48.77–58.46%; in 42–96 % of folds BAC > dummy classifier; see Figure 3B and Supporting Information Tables S14–S16). Thus, additional pipeline steps, that is, FS and HPO, which are commonly found to enhance performance, did not substantially increase classification accuracies in the current study (Brown & Hamarneh, 2016; Mwangi et al., 2014).

#### Classification performance across different feature sets and sample sizes for global cognition.

Classification performance for global cognition was also examined for varying feature sets (i.e., 21, 421, 1,200, 1,621) and sample sizes (matched vs. unmatched). No performance improvements could be observed for greater feature set sizes (Feature sets 21 and 421: M_BAC_ range: 48.42–59.31%, in 34–98% of folds BAC > dummy classifier; feature sets 1,200 and 1,621: M_BAC_ range: 48.96–58.72%, in 38–94% of folds BAC > dummy classifier) in both samples across pipeline configurations and algorithms (see Figure 3C and Supporting Information Tables S17–S20). A small difference between samples emerged in the nr condition. Relatively higher accuracies across feature sets were found in the nr condition of the unmatched sample than in the matched sample (Unmatched sample: M_BAC_ range nr: 49.33–59.31%, in 44–98% of folds BAC > dummy classifier; Matched sample: M_BAC_ range nr: 48.96–57.41%, in 40–86% of folds BAC > dummy classifier; see Supporting Information Tables S17–S20). This effect was no longer found in the cr condition (Unmatched sample: M_BAC_ range cr: 50.00–56.81%, in 42–94% of folds BAC > dummy classifier; Matched sample: M_BAC_ range cr: 48.42–58.33%, in 34–94% of folds BAC > dummy classifier; see Figure 3C and Supporting Information Tables S17–S20). ML performance in this specific case (nr condition/unmatched sample), however, is most likely influenced by confounds. Overall, findings suggest that increasing feature set and sample size may not systematically aid classification performance in our study. It, however, further underlines the relatively low discriminatory power of the specific RSFC strength measures for the research question at stake.

### Regression

#### Prediction performance of global cognition and cognitive domains across pipeline configurations.

**Figure 4.**
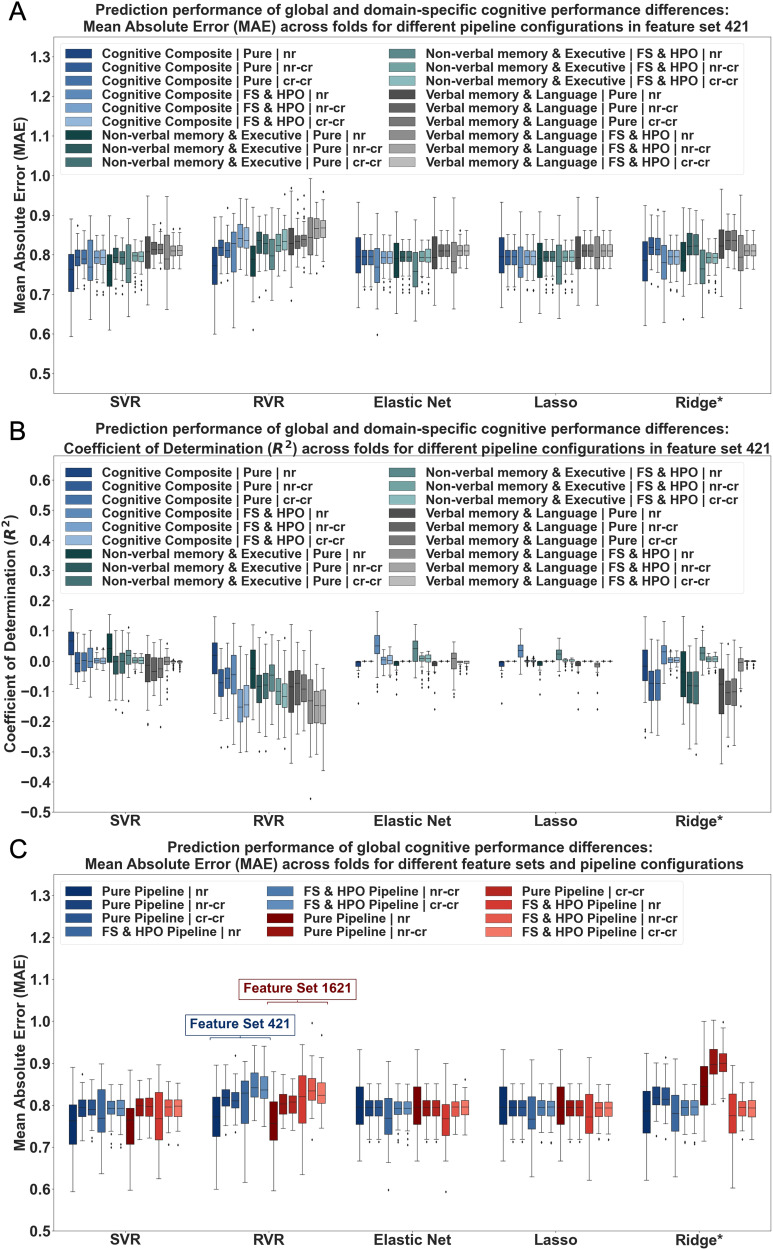
Regression performance results of cognitive performance differences (based on global and domain-specific cognitive scores) from RSFC strength measures. Regression performance across algorithms: Support Vector Regression (SVR), Relevance Vector Regression (RVR), Elastic Net, LASSO and Ridge Regression. Results shown for (A and B) cognitive composite and cognitive component scores, (A and C) different pipeline configurations (pure (no FS/HPO) vs. FS and HPO pipelines), and (C) feature set sizes (421, 1621) (C). Ridge*: default values in pure pipeline manually adjusted; nr = no confound regression; nr-cr = age, sex, and education regressed from target; cr-cr = age, sex, and education regressed from target and features.

In a second step, ML was used to assess whether RSFC strength measures can be used to continuously predict cognitive performance in older adults. ML prediction performance of global and domain-specific cognition from RSFC strength measures was initially evaluated in feature set 421 in the unmatched sample. Across pipeline configurations and deconfounding strategies, MAEs obtained for global and domain-specific cognition were high, ranging between 0.76 and 1.14 (see Figure 4A). Simultaneously, the coefficient of determination (*R*^2^) was found to be low (≤0.06) or even negative, indicating that predicting the mean of cognitive scores would have yielded better results than our model’s predictions (see Figure 4B and Supporting Information Tables S21 and S22). The NON-VERBAL MEMORY & EXECUTIVE component revealed slightly lower MAE and higher *R*^2^ than the VERBAL MEMORY & LANGUAGE component across conditions (see Figure 4A and B and Supporting Information Tables S21 and S22). Nevertheless, predictability compared to global cognition was similar in range. Furthermore, results were comparable for different algorithms except for Ridge regression in pure pipelines, which showed markedly elevated MAE, and reduced explained variance for all targets for default values of the hyperparameter lambda (see Supporting Information Table S21). Manual adjustment of the hyperparameter led to similar performance to other algorithms (see Figure 4A and B and Supporting Information Table S21). No systematic predictive performance differences were found for FS and HPO pipelines (see Figure 4A and B and Supporting Information Tables S21 and S22). In terms of different extents of deconfounding, the nr condition resulted in slightly better prediction results compared to the other two conditions (nr: MAEs ≥ 0.76; *R*^2^ ≤ 0.06; nr-cr and cr-cr: MAEs ≥ 0.79; *R*^2^ ≤ 0.00; see Supporting Information Table S21). This was also reflected in an improved robustness against the dummy regressor (see Figure 4C and Supporting Information Table S22). Nevertheless, it should be kept in mind that still only a limited number of models were consistently outperforming the dummy estimates in more than 80% of folds. Jointly, these results suggest that RSFC strength measures may not contain sufficient information to reliably predict global and domain-specific cognitive performance in older adults from a population-based cohort.

#### Prediction performance across varying feature set sizes for global cognition.

Feature set size did only have minimal impact in the classification setting. To verify the impact of varying feature combinations and number of features in ML prediction, feature set 421, which was used for comparability purposes throughout the analyses, and 1,621, which contains all possible features, were chosen for closer examination in the regression setting. Thus, ML performance estimations were examined in different pipeline configurations for global cognition. Across feature sets and deconfounding strategies, the *MAE* was again found to be high (≥0.75) and the coefficient of determination to be low (≤0.07) (see Supporting Information Tables S23 and S24). The impact of different algorithms, pipeline configurations, and extents of deconfounding on ML performance was again found to be minimal and to follow a similar pattern as before (see Figure 4C). No significant performance differences in terms of *MAE* and *R*^2^ emerged for different feature set sizes (see Figure 4C and Supporting Information Tables S23 and S24). Thus, findings suggest in addition to minimal discriminatory power also low predictive potential of cognitive performance differences in healthy older adults across feature sets, deconfounding strategies, and pipeline configurations from RSFC strength measures.

### Validation Analyses

Finally, we investigated the impact of a finer grained parcellation on ML performance. Results suggest that a higher granularity has only little impact on ML performance. Classification accuracies ranged between 47.79% and 56.53% across feature sets and pipeline configurations for the 800-node parcellation (see Supporting Information Tables S25 and S26 and Figure S28A), compared to the 48.42% to 58.33% range obtained for the 400-node parcellation. Prediction performance was found to be equally low as in the initial parcellation with high *MAE*s (≥0.75) and low to none explained variance (*R*^2^ ≤ 0.07) for different feature sets and pipeline configurations (see Supporting Information Table S27 and Figure S28B). Thus, no benefit of a higher granularity was observed. Furthermore, ML performance was examined in males and females separately. Classification performance in male and female samples equally did not exceed 60% accuracy for global cognition (M_BAC_: 49.69–55.57%; see Supporting Information Tables S29 and S30 and Figure S32A). Prediction performance in male and female samples revealed comparable high *MAE*s (≥0.73) and low *R*^2^ (≤0.04) (see Supporting Information Table S31 and Figure S32B). Findings, hence, further emphasize results found in the main analysis. Moreover, classification and prediction performance was assessed using connectivity estimates based on (i) positive and negative correlations and (ii) only negative correlations. For connectivity estimates based on positive and negative correlation values, classification performance varied between 47.91% to 56.25% BAC for global cognition across algorithms, feature sets and pipeline configurations (see Supporting Information Table S33 and Figure S35A). Prediction performance equally resembled results from the main analysis (*MAE*s ≥ 0.75; *R*^2^ ≤ 0.08; see Supporting Information Table S34 and Figure S35B). A similar pattern of results emerged for strength measures derived from negative correlations. Classification performance varied between 48.42% to 54.73% BAC for global cognition across algorithms, feature sets, and pipeline configurations (see Supporting Information Table S36). In turn, prediction performance was found to be equally low (*MAE*s ≥ 0.77; *R*^2^ ≤ 0.05; see Supporting Information Table S37). Adding further information from anticorrelations, thus, did not appear to improve ML performance. Furthermore, we investigated classification performance in extreme cognitive groups. Across samples, pipelines, feature sets, and algorithms, classification performance ranged between 49.70% to 62.50% BAC (see Supporting Information Tables S38 and S39). Although slightly better classification results were achieved for extreme cognitive groups, overall performance remained limited. This suggests that low classification results may not be primarily driven by difficulties in identifying participants close to the median and provides further sustenance to our findings from the main analyses. An age prediction and classification framework was chosen for validating our ML pipeline. In the classification of extreme age groups, highest classification performance was obtained for linear SVM in the pure and HPO pipeline with 85.13% and 83.13% accuracy, respectively (see Supporting Information Table S40). For the continuous prediction of age, RSFC strength measures were found to overall predict age reasonably well with *R*^2^ in the best cases ranging between 0.3 and 0.4 (extreme and whole sample across age spectrum; see Supporting Information Table S41). In comparison to dummy estimates, these models also showed reliably higher performance (see Supporting Information Table S42). While the obtained *MAE*s across samples were not competitive with those reported in the literature, results from the validation analyses, nevertheless, generally support the view that the current pipeline may yield reasonable prediction and classification performances (Liem et al., 2017; Pläschke et al., 2017; Vergun et al., 2013; Vieira et al., 2022). Thus, the low ML performance estimates may be specific to the setting of classifying and predicting cognitive performance differences from RSFC strength measures in healthy older adults rather than a general finding pertained to the ML setup, parcellation granularity, sampling, or features.

## DISCUSSION

The aim of the current investigation was to examine whether global and domain-specific cognitive performance differences may be successfully distinguished and predicted from RSFC strength measures in a large sample of older adults by using a systematic assessment of standard ML approaches. Results showed that classification and regression performance failed to reach adequate discriminatory and predictive power at the individual level. Importantly, these results persisted across different feature sets, algorithms, and pipeline configurations.

The present findings add to the notion that predicting cognition from the functional network architecture may yield heterogeneous findings (Dubois et al., 2018; Finn et al., 2015; Rasero et al., 2021; Vieira et al., 2022). For instance, RSFC patterns expressed in functional connectivity matrices have been shown to explain up to 20% of variance in a composite cognition score (NIH Cognitive Battery) and in a general intelligence factor (factor analysis) in two samples of the Human Connectome Project (HCP) S1200 young adult release (Dhamala et al., 2021; Dubois et al., 2018). In contrast, global cognition (NIH Cognitive Battery; cf. Dhamala et al., 2021) was predicted to a notably smaller degree from RSFC in young adults (median *R*^2^ = 0.016) (Rasero et al., 2021). In older adults, Vieira et al., (2022) reported RSFC to not predict prospective global cognitive decline, that is, change in two clinical assessments (OASIS-3 project; median *R*^2^_MMSE_ = 0; median *R*^2^_CDR_ = 0.01). Our results further emphasize that across different analytic choices RSFC strength measures may not reliably capture cognitive performance variations in older aged adults. In light of our goal of robust and accurate classification and prediction at the individual level, the minimum acceptable prediction accuracy is achieved only if the model outperforms the dummy estimate in more than 80% of the folds. This threshold is not met by the majority of our classification and prediction models, hinting at a limited potential as biomarker for age-related cognitive decline. Validation analyses further highlight the specificity of our results to cognitive abilities. RSFC strength measures could be used to successfully classify extreme age groups and moderately predict age (Meier et al., 2012; Pläschke et al., 2017; Vergun et al., 2013). RSFC patterns underlying cognition, however, may be more difficult to discern with current analytic tools, leading to mixed or null results. It should be stressed that null results may be highly informative as they provide important insights for future research, support a more realistic and unbiased view on brain-behavior relations, and allow for learning from experiences across the field (Janssen et al., 2018; Masouleh et al., 2019). Nevertheless, they tend to be underreported in the literature, leading to a potential publication bias (Janssen et al., 2018).

Successful prediction or classification of cognitive functioning from RSFC patterns has been reported previously (Dhamala et al., 2021; Dubois et al., 2018; Hojjati et al., 2017; Khazaee et al., 2016; Rosenberg et al., 2016; Yoo et al., 2018). One possible explanation for the fact that the results could not be replicated is related to the composition of the sample. Most previous studies reporting satisfactory ML performance focused on younger cohorts or patient populations (Dhamala et al., 2021; Dubois et al., 2018; Hojjati et al., 2017; Khazaee et al., 2016; Rosenberg et al., 2016; Yoo et al., 2018). In comparison to younger samples (*M*_age_ < 30), low discriminatory and predictive power in the current study may be attributable to a more complex link between RSFC and cognition evolving during the aging process (Dhamala et al., 2021; Dubois et al., 2018; Rosenberg et al., 2016; Yoo et al., 2018). Aging is not only associated with cognitive decline and functional network reorganization, but also with an increasing interindividual variability (Andrews-Hanna et al., 2007; Chan et al., 2014; Chong et al., 2019; Fjell et al., 2015; Grady et al., 2016; Habib et al., 2007; Hartshorne & Germine, 2015; Hedden & Gabrieli, 2004; Mowinckel et al., 2012; Ng et al., 2016; Onoda et al., 2012; Stumme et al., 2020). Consequently, the RSFC patterns that explain cognitive performance levels in older adults are more difficult to identify (Scarpazza et al., 2020).

When comparing promising patient classification results to the current results, effect sizes might be responsible for the unsatisfactory ML performance (Amaefule et al., 2021; Cui & Gong, 2018; Kwak et al., 2021). For example, patients with MCI and AD show markedly altered functional network organization compared to cognitively normal older adults (Badhwar et al., 2017; Brier et al., 2014; Buckner et al., 2009; Greicius et al., 2004; Sanz-Arigita et al., 2010; Wang et al., 2013). The sizable alterations related to pathological aging are reflected in encouraging results in patient classification (de Vos et al., 2018; Dyrba et al., 2015; Hojjati et al., 2017; Khazaee et al., 2016; Teipel et al., 2017). For instance, ML performance in patient classification (HC vs. MCI vs. AD) based on RSFC graph metrics reached above 88% accuracy (Hojjati et al., 2017; Khazaee et al., 2016). Nevertheless, these effect sizes may not be found for healthy older populations. For instance, cognition could be significantly predicted in samples of cognitive normal and clinically impaired older adults from whole-brain RSFC patterns (*r* = 0.08–0.44) (Kwak et al., 2021). However, prediction accuracy dropped substantially once models were trained only on clinically unimpaired older adults (*r* = −0.04–0.24) (Kwak et al., 2021). Accurate cognitive performance prediction from RSFC patterns in older aged adults without the inclusion of clinical populations may, hence, be impeded by small effect sizes.

Another aspect that needs to be addressed when discussing the low ML performance concerns the cognitive parameters used. Most studies including older cohorts have focused on specific cognitive abilities (Avery et al., 2020; Fountain-Zaragoza et al., 2019; Gao et al., 2020; Kwak et al., 2021; Pläschke et al., 2020). For instance, WM capacity could be successfully predicted from meta-analytically defined RSFC networks in older individuals (Pläschke et al., 2020). This may be due to a more explicit mapping of RSFC patterns to specific cognitive abilities than for general or clustered cognitive abilities, which we were interested in (Avery et al., 2020; Gao et al., 2020; Kwak et al., 2021).

Furthermore, most prior studies have used pair-wise functional connectivity as input features (Avery et al., 2020; Dhamala et al., 2021; Dubois et al., 2018; Gao et al., 2020; He et al., 2020; Pläschke et al., 2020). We used functional connectivity estimates linked to cognitive performance differences in aging and with promising classification performance in neurodegenerative diseases (Chan et al., 2014; Hausman et al., 2020; Hojjati et al., 2017; Iordan et al., 2018; Khazaee et al., 2016; Malagurski et al., 2020; Ng et al., 2016; Stumme et al., 2020). Findings highlight that for reliably detecting cognitive performance differences in normally aging individuals, the additional dimensionality reduction inherent to the calculation of RSFC strength values may be too extensive, that is, relevant information for ML was lost during the computation (Cui & Gong, 2018; Lei et al., 2020). Also, redundancy of feature information, that is, within- and inter-network connectivity, may have resulted in poorer ML performance, especially in larger feature sets (Mwangi et al., 2014).

### Methodological Considerations and Future Outlook

While the current investigation concentrated on RSFC strength measures, future studies might use other imaging features, that is, more complex graph metrics, such as betweenness centrality or modularity, multimodal or task-based fMRI data, to improve the prediction of cognitive performance in older age (Draganski et al., 2013; Gbadeyan et al., 2022; McConathy & Sheline, 2015; Pacheco et al., 2015; Sripada et al., 2020b; Vieira et al., 2022). For example, prior research has shown that global cognitive abilities could be better predicted from task-based than resting-state fMRI data in large samples of younger adults from the HCP dataset (Greene et al., 2018; Sripada et al., 2020a). Along these lines, it may be interesting to investigate whether task-based fMRI data in these circumstances also outperforms RSFC in older adults. Likewise, it is also warranted to keep a distinction between basic research and clinical applicability. Classification and prediction results might already be informative, if they are statistically significant in healthy subjects; however, they may not be practically relevant for the clinical context.

Furthermore, only cross-sectional data has been used in the current investigation. Although important insights can be gained cross-sectionally, the investigation of longitudinal data becomes indispensable in the biomarker development for prospective age-related cognitive decline (Davatzikos et al., 2009; Liem et al., 2021). Initial efforts to predict future cognitive decline from imaging and nonimaging data have revealed promising results (Vieira et al., 2022).

A further methodological consideration pertains to the choice of data preparation steps, for example, the parcellation scheme and choice of network assignment (Dubois et al., 2018). In the current investigation, a functional network parcellation derived from younger brains was used, which directly links brain networks to behavioral processing and is commonly used in lifespan studies (Schaefer et al., 2018; Yeo et al., 2011). Although ML performance in the current study was low regardless of data preparation, that is, parcellation granularity, and ML model choices, future studies are warranted to examine generalizability to other population-based cohorts of older aged adults and other functional network divisions.

### Conclusions

The present study addressed the biomarker potential of RSFC strength measures for cognitive performance differences in normal aging in a systematic evaluation of standard ML approaches. Present results across different analytic choices emphasize that the potential of RSFC strength measures as sole biomarker for age-related cognitive decline may be limited. Findings add to past research demonstrating that reliable cognitive performance prediction and distinction in healthy older adults based on RSFC strength measures may be challenging due to small effects, high heterogeneity, and the removal of relevant information during the computation of these parameters. Although current results are far from promising, they still may prove useful in providing guidance on future research targets. Specifically, multimodal and longitudinal approaches appear warranted in future studies developing a robust biomarker for cognitive performance in healthy aging.

## ACKNOWLEDGMENTS

This project was partially funded by the German National Cohort and the 1000BRAINS-Study of the Institute of Neuroscience and Medicine, Research Centre Jülich, Germany. We thank the Heinz Nixdorf Foundation (Germany) for the generous support of the Heinz Nixdorf Study. We thank the investigative group and the study staff of the Heinz Nixdorf Recall Study and 1000BRAINS. This research was supported by the Joint Lab Supercomputing and Modeling for the Human Brain. The authors gratefully acknowledge the computing time granted through JARA on the supercomputer JURECA (Jülich Supercomputing Centre, 2021) at Forschungszentrum Jülich.

## SUPPORTING INFORMATION

Supporting information for this article is available at https://doi.org/10.1162/netn_a_00275.

## AUTHOR CONTRIBUTIONS

Camilla Krämer: Conceptualization; Formal analysis; Methodology; Visualization; Writing – original draft; Writing – review & editing. Johanna Stumme: Formal analysis; Methodology; Writing – review & editing. Lucas da Costa Campos: Formal analysis; Methodology; Writing – review & editing. Christian Rubbert: Methodology; Writing – review & editing. Julian Caspers: Conceptualization; Methodology; Writing – review & editing. Svenja Caspers: Conceptualization; Funding acquisition; Resources; Supervision; Writing – review & editing. Christiane Jockwitz: Conceptualization; Methodology; Supervision; Writing – review & editing.

## FUNDING INFORMATION

Svenja Caspers, European Union’s Horizon 2020 Research and Innovation Programme (HBP SGA3), Award ID: Grant Agreement No. 945539.

## TECHNICAL TERMS

Machine learning (ML): Set of methods used to automatically find patterns in data that allow classification and prediction.
Global cognition: General cognitive ability that encompasses cognitive functioning across different domains.
Inter-network RSFC: Connectivity strength estimate of one node (nodal) or all nodes (network) within a network to all nodes outside its network.
Ratio-score: A metric capturing within-network RSFC of one node (nodal) or all nodes (network) within a network in relation to its inter-network RSFC.
Within-network RSFC: Connectivity strength estimate of one node (nodal) or all nodes (network) within a network to all nodes within its network.
Feature set: The specific combination of input features used in ML.
Pipeline configuration: A specific setup of an ML pipeline to be tested in the analysis.
Domain-specific cognition: Cognitive processes that are linked and dedicated to specific mental abilities, e.g., executive and memory functions.
Deconfounding strategy: The approach of how to control for the impact of potential confounders, e.g., age or sex.
