# Supplementary material for: Classification and prediction of cognitive performance differences in older age based on brain network patterns using a machine learning approach
Source: Netw Neurosci. 2023 Jan 1;7(1):122–47. doi: 10.1162/netn_a_00275 (PMC10270720; doi:10.1162/netn_a_00275)
Supplement: Supplementary file 1 [file netn-7-1-122-s001.pdf]

Krämer, C., Stumme, J., da Costa Campos, L., Rubbert, C, Caspers, J., Caspers, S. & Jockwitz, C. (2023). Supporting information for "Classification and prediction of cognitive performance differences in older age based on brain network patterns using a machine learning approach." *Network Neuroscience*, *7*(1), 122–147. https://doi.org/10.1162/netn_a_00275

Supplementary Material

Supplementary Methods

Supplementary Table S1. Overview and description of neuropsychological tests employed in the current study to assess cognitive performance.

Domain	Function	Test	Description	Abbrev.	Mean (raw ± SD)	Z-score		
						Min	Max	Range
Attention	Selective Attention	Alters-Konzentrations-Test (Gatterer, 2008)	Time (sec.) to find and cancel targets among distractors	AKT	34.36 ± 10.66	-1.63	8.22	9.85
	Processing speed	Trail Making Test (part A) (taken from CERAD-Plus; Morris et al., 1989)	Time (sec.) to connect randomly arranged numbers in ascending order (part A)	TMT-A	40.04± 13.90	-1.73	6.31	8.04
Executive functions	Susceptibility to interference	Farb-Wort-Interferenztest (Jülich version; similar to: Bäumler, 1985; Stroop, 1935)	Time difference (sec.) between naming the ink in which color words were printed (part 3) and naming the color of boxes (part 2)	Stroop	43.09 ± 23.22	-1.71	11.41	13.12
	Figural fluency	Fünf-Punkte-Test (Jülich version; similar to: Regard et al., 1982)	Total number of unique patterns created by connecting 5 dots in (3 minutes)	FPT	26.65 ± 7.54	-3.00	3.36	6.36
	Problem solving	Leistungsprüfungssystem 50+ (Subtest 3) (Sturm et al., 1993)	Total number of correctly tagged irregularities in sequences of geometric figures (5 minutes)	LPS	20.75 ± 5.10	-3.09	2.79	5.88
	Concept shifting	Trail Making Test (part B-A) (taken from CERAD-Plus; Morris et al., 1989)	Time difference (sec.) between connecting numbers and letters in ascending order in an alternating fashion (part B) and (part A)	TMT-BA	54.30 ± 40.95	-1.68	7.95	9.62
Language	Vocabulary	Wortschatztest (Schmidt & Metzler, 1992)	Total number of correctly identified real words within rows of pseudo words	AWSTOP3	30.96 ± 4.90	-5.90	2.05	7.95
	Semantic verbal fluency	Regensburger Wortflüssigkeitstest (Aschenbrenner et al., 2000)	Total number of produced words in the category "Berufe (job)" (2 minutes)	RWT-S	23.98 ± 6.68	-2.84	3.89	6.73
	Phonemic verbal fluency	Regensburger Wortflüssigkeitstest (Aschenbrenner et al., 2000)	Total number of produced words beginning with the letter B (2 minutes)	RWT-P	18.53 ± 6.25	-2.64	3.27	5.91
Episodic memory	Figural memory	Benton-Test (Benton et al., 2009)	Total number of errors made during the free recall of 20 previously presented figures	Benton	16.41 ± 8.11	-1.9	3.53	5.43
	Verbal episodic memory	Verbaler Gedächtnistest (Lux et al., 2012)	Total number of free recalled words from a list of 15 words across 5 trials (sum score)	BKW	42.06 ± 10.25	-3.52	2.34	5.85
Working memory	Visual spatial working memory	Block-Tapping-Test (Schelling, 1997)	Total number of correctly repeated blocks given in a sequence (sum score backward and forward)	CBT	10.12 ± 1.57	-4.52	3.10	7.62
	Visual working memory	Visual pattern (Jülich version; similar to: Della Sala et al., 1997)	Total number of correctly memorized matrix patterns of black and white squares with increasing complexity	VPT	7.79 ± 1.78	-2.13	2.36	4.49
	Verbal working memory	Zahlennachsprechen (from Nürnberger Alters-Inventar) (Oswald & Fleischmann, 1997)	Total number of correctly recalled digits given in a sequence (sum score backward and forward)	ZNS	10.80 ± 1.80	-2.65	3.98	6.64

Further descriptions are also found in Caspers et al. (2014) as well as Jockwitz et al. (2017).

Machine learning (ML) classification algorithms

Support Vector Machine (SVM). Support vector machine is a widely used classification method due to its ability to deal with high-dimensional data, flexibility and stable results (Schölkopf, Tsuda & Vert, 2004). It targets the classification of data points by maximizing the margin between classes in high-dimensional space (Pereira, Mitchell & Botvinick, 2009; Vapnik, 1995). This is achieved by transformation of training data into high dimensional-space and the calculation of a hyperplane that best separates between groups (Schölkopf & Smola, 2002). Only points on the margin, known as support vectors, are taken into account for determining the optimal hyperplane.

K-Nearest Neighbour (KNN). K-Nearest Neighbour has been argued to be one of the simplest ML algorithms. It classifies an input vector by assigning the individual target to the most similar group. The number of k neighbours or data points closest to the specific object vote on the class or group the example object may belong to (Erickson, Korfiatis, Akkus & Kline, 2017).

Decision Tree (DT). Decision Trees are non-parametric supervised learning methods. They are built on the idea that classification of groups can be achieved by establishing simple decision rules from input features. Binary trees are built by starting with the whole data set and recursively partitioning the feature space into smaller parts using a top-down search approach (Quinlan, 1986). An impurity criterion, such as Gini index or cross-entropy index, is minimized as the feature space is split (Chen, Kar, & Ralescu, 2012; Foody, 1995; Loh, 2011). Splitting of the tree continues until a termination criterion is met.

Naïve Bayes (NB). Naïve Bayes classifier fall into the category of probabilistic approaches to inference in ML. Group or class membership is determined by applying Bayes theorem on conditional probabilities assuming conditional independence (Serra, Galdi & Tagliaferri, 2018).

Linear Discriminant Analysis (LDA). Linear Discriminant Analysis constitutes a linear classifier operating on the assumption of a common covariance matrix of classes (Fisher, 1936; Hastie, Tibshirani & Friedman, 2009). Separation of classes is achieved by determining a linear combination of input variables that maximize the between-group and minimize the within-group variance (McLachlan, 2004).

Machine learning (ML) regression algorithms

Support Vector Regression (SVR). Support Vector Regression is an extended SVM method and aims at finding a regression line fitting as many data instances as possible (Drucker et al., 1997). In this context, a tube of width $\epsilon > 0$ is fitted to the data with some points falling inside the tube and some falling outside (Mohri, Rostamizadeh & Talwakar, 2018). Data points are penalized based on their distance to the regression line with points inside the tube and close to the regression line not being penalized (Mohri, Rostamizadeh & Talwakar, 2018). SVR models, thus, depend on those points laying outside or on the border of the tube known as support vectors.

Relevance Vector Regression (RVR). Relevance Vector Regression is a sparse kernel method in a Bayesian framework as an alternative to SVR (Bishop, 2006; Tipping, 2001). Relevance vectors differ from support vectors in one main aspect: while support vectors represent separating features, relevance vectors display the most prototypical examples of a ML task (Gaser et al., 2013). Due to the use of a prior in the estimation of model parameters, fewer relevance vectors compared to the number of support vectors in SVR are chosen (Tipping, 2001).

Ridge Regression (Ridge). Ridge regression is a linear regression model that adds a regularization penalty (L2-norm) to the loss function of ordinary squares least (OLS) regression (Hoerl & Kennard, 1970). The L2-penalty continuously reduces the size of the regression coefficients, while at the same time preventing them to become zero (Hoerl & Kennard, 1970).

Least Absolute Shrinkage and Selection Operator Regression (LASSO). In LASSO regression, a L1-regularization penalty is applied to the OLS loss function (Tibshirani, 1996). Besides continuously shrinking the coefficients, the L1-norm sets some coefficients to zero yielding sparse models, i.e. implementing feature selection (Tibshirani, 1996).

Elastic Net Regression (Elastic Net). Elastic Net regression can be viewed as a combination of Lasso and Ridge regression using both a L1- and L2-regularization norm. Similarly to LASSO, it produces a sparse solution and with it integrates feature selection into its optimization problem (Zou & Hastie, 2005). Further, Elastic Net may allow the grouping of highly correlated features and for making a joint decision of whether to include them into the model or not (Zou & Hastie, 2005).

Validation analysis: Classification of extreme cognitive groups (high vs. low cognitive performance group)

Supplementary Table S2. Demographic information for extreme cognitive samples used in validation analyses.

	Extreme Sample Unmatched			Extreme Sample Matched		
	N	M_{age}	Edu.	N	M_{age}	Edu.
Female	171	67.33 (7.22)	5.81 (1.92)	63	64.15 (5.23)	5.63 (1.56)
Male	235	67.22 (7.12)	6.82 (1.97)	85	67.39 (6.40)	6.56 (1.79)
Total	406	67.26 (7.15)	6.40 (2.01)	148	66.01 (6.13)	6.17 (1.75)

Note. Standard deviation (SD) appears in parentheses.

Supplementary Table S3. Demographic information for high and low performance groups in extreme sample used in validation analyses.

	Extreme Sample Unmatched			Extreme Sample Matched		
	N	M_{age}	Edu.	N	M_{age}	Edu.
High	203 (76 F)	63.10 (5.26)	7.45 (1.86)	74 (33 F)	65.25 (5.90)	6.12 (1.72)
Low	203 (95 F)	71.43 (6.33)	5.34 (1.55)	74 (30 F)	66.77 (6.29)	6.22 (1.79)

Note. Standard deviation (SD) appears in parentheses. F = females.

Validation analysis: Age classification & prediction

For age classification and prediction, two additional samples were drawn from the 1000BRAINS study. For the classification setting, extreme age groups (young vs old) were defined. 433 participants fell within the age range 20 to 35 years (N=86; young group) and the age range 70 to 85 years (N=347; old group).

From this initial sample, 82 participants were excluded due to missing resting-state fMRI data, methodological problems of functional imaging data, in a quality control step of the preprocessed functional data or in a sensitivity analyses based on the 800-node parcellation (N=351; same QC steps as in the main analysis). To achieve balanced groups, old and young age groups were matched for sex and education using propensity score matching, which yielded a final sample of 106 participants (27 females, M_{age} = 52.13, SD_{age} = 23.06, see Supplementary Table S2). For the classification, the sample was further divided into two groups of 53 participants (young vs. old) (see Supplementary Table S3 for demographic information).

In the prediction setting, the whole age range (18-87 years) of 1000BRAINS was additionally examined (N=1309). From the total sample, 240 subjects were excluded due to missing fMRI data, methodological problems of functional imaging data, in an additional quality control step or in a sensitivity analyses based on the 800-node parcellation (same QC steps as in main analysis). This led to a final sample of 1069 participants (486 females, M_{age} = 61.61, SD_{age} = 12.72, see Supplementary Table S2) to be used in ML. Age was, thus, continuously predicted in the extreme age group sample, the whole age range sample and the unmatched sample from the main analysis.

Supplementary Table S4. Demographic information for samples used in validation analyses.

	Whole Sample			Extreme Sample		
	N	M_{age}	Education	N	M_{age}	Education
Female	486	61.59 (11.94)	6.18 (1.89)	27	44.24 (21.74)	7.33 (1.24)
Male	583	61.63 (13.35)	7.10 (1.87)	79	54.82 (23.01)	7.95 (1.23)
Total	1069	61.61 (12.72)	6.68 (1.93)	106	52.13 (23.06)	7.79 (1.26)

Note. Standard deviation (SD) appears in parentheses.

Supplementary Table S5. Demographic information for high and low performance groups in extreme sample used in classification validation.

	N	M_{age}	Education
High	53 (9 F)	74.81 (3.18)	7.79 (1.26)
Low	53 (18 F)	29.45 (3.86)	7.79 (1.26)

Note.
Standard deviation (SD) appears in parentheses. F = females.

Supplementary Results

Supplementary Table S6. Explained variance and eigenvalues from PCA in the unmatched sample.

Component	Initial Eigenvalues			Rotation Sums of Squared Loadings		
	Total	% of Variance	Cumulative %	Total	% of Variance	Cumulative %
1	5.18	37.02	37.02	3.79	37.02	37.02
2	1.28	9.19	46.21	2.68	9.19	46.21
3	.99	7.04	53.25			
4	.90	6.45	59.71			
5	.76	5.42	65.12			
6	.72	5.15	70.27			
7	.67	4.80	75.07			
8	.62	4.44	79.52			
9	.57	4.04	83.56			
10	.54	3.86	87.41			
11	.50	3.57	90.98			
12	.47	3.33	94.3			
13	.42	3.00	97.3			
14	.38	2.70	100.0			

Supplementary Table S7. Factor loadings from PCA in the unmatched sample.

Cognitive Test	Factor Loadings		
	Cognitive Composite	Non-verbal Memory & EF	Verbal Memory & Language
FPT	0.642	0.503	0.400
LPS	0.754	0.690	0.337
AWSTO3P	0.611	0.289	0.635
RWT-P	0.504	0.054	0.771
RWT-S	0.558	0.144	0.741
BKW	0.536	0.270	0.535
CBT	0.518	0.723	-0.104
VPT	0.630	0.746	0.054
ZNS	0.554	0.481	0.281
TMT-A	0.580	0.534	0.254
AKT	0.606	0.550	0.277
TMT-BA	0.632	0.527	0.350
Stroop	0.542	0.449	0.304
Benton	0.779	0.712	0.349

Supplementary Figure S8. Scree plot of initial eigenvalues in the unmatched sample derived from PCA.

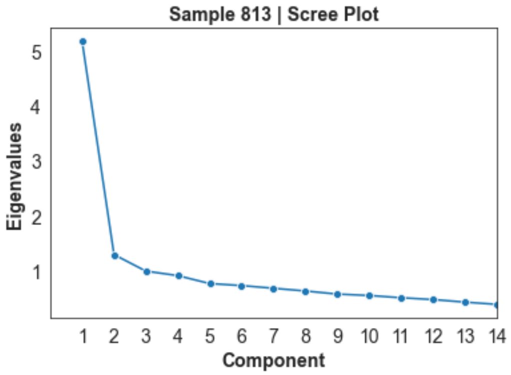

Supplementary Table S9. Factor loadings from PCA in original unmatched sample, in random split-half samples of the unmatched sample and a validation sample without participants with missing values in any of the cognitive tests.

	Original sample			Split half sample 1			Split half sample 2			Validation sample		
Cog. Test	Composite	Component 1	Component 2	Composite	Component 1	Component 2	Composite	Component 1	Component 2	Composite	Component 1	Component 2
FPT	.64	.50	.40	.68	.54	.42	.61	.46	.40	.64	.50	.40
LPS	.75	.69	.34	.78	.72	.33	.73	.62	.39	.75	.69	.33
AWSTO3P	.61	.29	.64	.62	.29	.66	.60	.26	.64	.62	.28	.65
RWT-P	.50	.05	.77	.47	.01	.80	.54	.09	.73	.50	.05	.76
RWT-S	.56	.14	.74	.54	.13	.75	.58	.14	.74	.54	.13	.73
BKW	.54	.27	.54	.53	.30	.49	.54	.23	.58	.56	.29	.54
CBT	.52	.72	-.10	.53	.71	-.08	.51	.74	-.11	.50	.72	-.12
VPT	.63	.75	.05	.65	.75	.06	.62	.73	.08	.63	.75	.05
ZNS	.55	.48	.28	.56	.50	.27	.54	.45	.32	.55	.48	.27
TMT-A	.58	.53	.25	.56	.55	.19	.60	.53	.30	.57	.53	.25
AKT	.61	.55	.28	.66	.63	.24	.56	.46	.33	.62	.55	.29
TMT-BA	.63	.53	.35	.63	.54	.34	.63	.52	.36	.63	.51	.37
Stroop	.54	.45	.30	.50	.37	.34	.59	.53	.27	.53	.43	.31
Benton	.78	.71	.35	.79	.71	.37	.77	.70	.36	.78	.71	.35

Note. Composite = Cognitive composite; Component 1 = Non-verbal memory & EF; Component 2 = Verbal Memory & Language.

Supplementary Table S10. Correlations between factor loadings from different samples.

		Composite				Component 1				Component 2			
		Original	Split Half 1	Split Half 2	Validation	Original	Split Half 1	Split Half 2	Validation	Original	Split Half 1	Split Half 2	Validation
Composite	Original	1	.97**	.96**	.99**	-	-	-	-	-	-	-	-
	Split Half 1	.97**	1	.86**	.98**	-	-	-	-	-	-	-	-
	Split Half 2	.96**	.86**	1	.93**	-	-	-	-	-	-	-	-
	Validation	.99**	.98**	.93**	1	-	-	-	-	-	-	-	-
Component 1	Original	-	-	-	-	1	.99**	.98**	.99**	-	-	-	-
	Split Half 1	-	-	-	-	.99**	1	.94**	.99**	-	-	-	-
	Split Half 2	-	-	-	-	.98**	.94**	1	.97**	-	-	-	-
	Validation	-	-	-	-	.97**	.99**	.99**	1	-	-	-	-
Component 2	Original	-	-	-	-	-	-	-	-	1	0.99**	0.99**	0.99**
	Split Half 1	-	-	-	-	-	-	-	-	0.99**	1	0.97**	0.99**
	Split Half 2	-	-	-	-	-	-	-	-	0.99**	0.97**	1	0.99**
	Validation	-	-	-	-	-	-	-	-	0.99**	0.99**	0.99**	1

Note. **Correlation significant at the 0.01 level.

Classification

Supplementary Table S11. Classification performance for global and domain-specific cognition across pure and univariate FS (ANOVA F-test) pipeline.

	Pure						FS					
	COGNITIVE COMPOSITE		NON-VERBAL MEMORY & EXECUTIVE		VERBAL MEMORY & LANGUAGE		COGNITIVE COMPOSITE		NON-VERBAL MEMORY & EXECUTIVE		VERBAL MEMORY & LANGUAGE	
	nr	cr	nr	cr	nr	cr	nr	cr	nr	cr	nr	cr
SVM (RBF)	57.41% (6.21%)	58.33% (6.02%)	58.44% (5.76%)	56.93% (7.15%)	54.12% (5.95%)	53.21% (5.69%)	57.13% (7.08%)	57.59% (7.10%)	55.18% (8.60%)	56.27% (8.68%)	54.93% (5.85%)	52.71% (5.79%)
SVM (linear)	52.77% (6.69%)	52.20% (6.68%)	55.57% (5.91%)	53.07% (6.56%)	54.86% (5.04%)	54.82% (6.50%)	55.87% (7.57%)	56.33% (7.21%)	56.10% (7.23%)	54.98% (7.70%)	54.09% (6.73%)	53.39% (5.53%)
SVM (poly)	53.25% (5.50%)	51.96% (6.43%)	56.61% (5.30%)	54.53% (7.09%)	53.72% (6.46%)	50.51% (6.27%)	56.09% (6.21%)	54.78% (6.30%)	56.41% (6.35%)	55.01% (5.39%)	53.54% (6.05%)	51.23% (5.66%)
KNN	52.90% (6.43%)	52.05% (7.39%)	53.92% (5.33%)	53.55% (6.63%)	52.01% (6.44%)	50.21% (6.06%)	53.74% (6.31%)	54.57% (6.93%)	53.88% (7.14%)	50.92% (7.91%)	51.91% (6.09%)	52.16% (6.59%)
DT	50.13% (7.62%)	48.68% (6.38%)	50.31% (5.50%)	51.17% (7.49%)	50.92% (6.20%)	51.59% (6.85%)	51.34% (6.65%)	51.62% (6.79%)	51.48% (5.62%)	51.74% (6.92%)	50.29% (7.78%)	51.01% (7.19%)
NB	52.94% (6.17%)	52.24% (6.71%)	55.41% (6.41%)	53.49% (6.19%)	50.96% (7.07%)	50.51% (6.33%)	52.98% (6.62%)	52.47% (5.99%)	53.85% (7.56%)	52.08% (6.48%)	55.18% (6.50%)	54.40% (6.27%)
LDA	51.04% (7.76%)	50.37% (7.69%)	51.89% (7.91%)	50.67% (8.11%)	53.11% (7.05%)	52.31% (6.50%)	54.72% (7.77%)	56.13% (6.35%)	56.34% (7.69%)	56.03% (7.14%)	53.59% (7.61%)	53.45% (6.59%)

Note. Mean balanced accuracies (BAC (%)) displayed with standard deviation (SD) appearing in parentheses. Classification results across algorithms: Support Vector Machine (SVM) with Radial Basis Function (RBF), linear and polynomial (poly) kernel, K Nearest Neighbour (KNN), Decision Tree (DT), Naïve Bayes (NB), Linear Discriminant Analysis (LDA). nr = no confound regression; cr = age, sex & education regressed from features.

Supplementary Table S12. Classification performance for global and domain-specific cognition across pure and univariate FS (ANOVA F-test) pipeline in comparison to a dummy classifier.

	Pure						FS					
	COGNITIVE COMPOSITE		NON-VERBAL MEMORY & EXECUTIVE		VERBAL MEMORY & LANGUAGE		COGNITIVE COMPOSITE		NON-VERBAL MEMORY & EXECUTIVE		VERBAL MEMORY & LANGUAGE	
	nr	cr	nr	cr	nr	cr	nr	cr	nr	cr	nr	cr
SVM (RBF)	86%	94%	92%	82%	70%	66%	82%	82%	70%	72%	76%	72%
SVM (linear)	58%	52%	86%	68%	84%	70%	80%	78%	78%	70%	70%	70%
SVM(poly)	66%	54%	88%	78%	68%	48%	80%	72%	84%	82%	72%	48%
KNN	66%	60%	80%	70%	62%	48%	64%	68%	70%	48%	64%	66%
DT	46%	40%	48%	48%	46%	52%	52%	54%	54%	52%	44%	44%
NB	58%	58%	80%	68%	44%	42%	62%	58%	68%	58%	80%	74%
LDA	62%	52%	60%	52%	60%	62%	76%	86%	72%	76%	64%	70%

Note. Percentage (%) of folds for which real models outperform dummy classifier in terms of balanced accuracy (BAC). Classification results across algorithms: Support Vector Machine (SVM) with Radial Basis Function (RBF), linear and polynomial (poly) kernel, K Nearest Neighbour (KNN), Decision Tree (DT), Naïve Bayes (NB), Linear Discriminant Analysis (LDA). nr = no confound regression; cr = age, sex & education regressed from features. Colour scheme: green = real model outperforms dummy in ≥ 80% of folds; orange = real model outperforms dummy in 50 - 80% of folds; red = real model outperforms dummy in ≤ 50% of folds.

Supplementary Table S13. Classification performance (sensitivity & specificity) for global and domain-specific cognition across pure and univariate FS (ANOVA F-test) pipeline.

		COGNITIVE COMPOSITE				NON-VERBAL MEMORY & EXECUTIVE				VERBAL MEMORY & LANGUAGE			
		nr		cr		nr		cr		nr		cr	
		Sens	Spec	Sens	Spec	Sens	Spec	Sens	Spec	Sens	Spec	Sens	Spec
Pure	SVM	59.08%	55.74%	58.92%	57.74%	60.99%	55.89%	61.05%	52.80%	52.99%	55.25%	51.18%	55.25%
	(RBF)	(10.03%)	(9.85%)	(8.06%)	(7.60%)	(9.04%)	(10.18%)	(8.31%)	(9.14%)	(10.16%)	(9.5%)	(8.24%)	(7.51%)
	SVM	52.50%	53.05%	51.50%	52.90%	57.33%	53.82%	54.23%	51.91%	52.65%	57.06%	53.47%	56.17%
	(linear)	(11.21%)	(10.5%)	(7.43%)	(8.78%)	(10.6%)	(8.95%)	(8.30%)	(8.51%)	(10.67%)	(7.48%)	(8.42%)	(6.93%)
	SVM	32.06%	74.44%	29.80%	74.13%	42.01%	71.21%	43.81%	65.24%	47.10%	60.33%	39.57%	61.44%
	(poly)	(13.05%)	(10.15%)	(10.90%)	(8.03%)	(10.80%)	(11.74%)	(11.62%)	(12.80%)	(12.72%)	(12.68%)	(12.34%)	(11.16%)
	KNN	62.31%	43.48%	56.44%	47.66%	60.16%	47.68%	57.50%	49.61%	56.75%	47.27%	51.92%	48.51%
		(9.03%)	(9.46%)	(9.63%)	(9.98%)	(8.55%)	(9.38%)	(8.50%)	(10.55%)	(9.42%)	(8.46%)	(9.91%)	(8.29%)
	DT	48.89%	51.37%	47.19%	50.17%	53.75%	46.87%	52.47%	49.86%	50.04%	51.79%	51.69%	51.48%
		(9.29%)	(11.61%)	(10.36%)	(9.43%)	(9.39%)	(9.8%)	(10.26%)	(12.57%)	(9.02%)	(8.69%)	(10.20%)	(10.04%)
	NB	43.56%	62.32%	42.63%	61.84%	44.57%	66.26%	41.44%	65.53%	50.09%	51.83%	48.90%	52.12%
		(9.92%)	(8.92%)	(8.33%)	(6.91%)	(9.37%)	(10.37%)	(6.80%)	(7.58%)	(14.33%)	(8.19%)	(10.89%)	(7.94%)
	LDA	50.03%	52.06%	49.04%	51.70%	52.21%	51.58%	50.51%	50.83%	52.30%	53.92%	52.41%	52.21%
		(11.29%)	(9.15%)	(8.02%)	(9.08%)	(11.15%)	(11.39%)	(9.10%)	(9.13%)	(10.51%)	(8.19%)	(7.24%)	(7.28%)
FS	SVM	57.52%	56.74%	56.20%	58.98%	56.32%	54.04%	58.57%	53.96%	54.47%	55.39%	51.98%	53.43%
	(RBF)	(10.68%)	(9.95%)	(8.60%)	(8.76%)	(12.05%)	(10.57%)	(10.01%)	(9.70%)	(11.46%)	(8.00%)	(8.51%)	(7.52%)
	SVM	54.66%	57.08%	53.12%	59.54%	56.70%	55.50%	55.30%	54.66%	50.70%	57.49%	52.09%	54.69%
	(linear)	(11.02%)	(9.87%)	(8.32%)	(7.78%)	(9.40%)	(10.60%)	(9.10%)	(9.20%)	(8.97%)	(9.79%)	(8.02%)	(7.16%)
	SVM	37.77%	74.42%	35.52%	74.03%	35.31%	77.51%	36.66%	73.36%	55.53%	51.56%	51.43%	51.03%
	(poly)	(10.83%)	(8.39%)	(11.29%)	(8.17%)	(11.91%)	(8.08%)	(9.32%)	(8.80%)	(15.04%)	(12.55%)	(12.60%)	(10.05%)
	KNN	63.86%	43.62%	64.68%	44.47%	66.21%	41.55%	58.20%	43.63%	58.87%	44.94%	58.73%	45.59%
		(9.13%)	(8.84%)	(10.67%)	(7.88%)	(9.36%)	(9.71%)	(11.03%)	(10.20%)	(9.50%)	(8.93%)	(10.31%)	(7.79%)
	DT	51.18%	51.50%	50.96%	52.28%	52.70%	50.26%	53.78%	49.70%	49.44%	51.14%	51.14%	50.88%
		(10.47%)	(8.89%)	(9.05%)	(9.38%)	(9.31%)	(8.60%)	(10.38%)	(8.38%)	(12.06%)	(9.90%)	(7.73%)	(11.69%)
	NB	44.25%	61.70%	44.32%	60.62%	44.50%	63.20%	41.23%	62.94%	56.01%	54.36%	54.31%	54.49%
		(11.05%)	(9.94%)	(7.46%)	(6.83%)	(9.19%)	(10.98%)	(7.26%)	(7.64%)	(12.58%)	(9.30%)	(9.57%)	(8.78%)
	LDA	54.13%	55.30%	53.74%	58.53%	57.02%	55.66%	56.38%	55.69%	49.76%	57.43%	51.45%	55.44%
		(10.58%)	(10.70%)	(8.19%)	(6.17%)	(10.83%)	(10.55%)	(9.51%)	(7.47%)	(8.50%)	(11.10%)	(8.30%)	(8.74%)

Note. Mean sensitivity (Sens, %) and specificity (Spec, %) displayed with standard deviation (SD) appearing in parentheses. nr = no confound regression; cr = age, sex & education regressed from features.

Supplementary Table S14. Classification performance across different feature selection (FS) and hyperparameter optimization (HPO) pipelines for global cognition.

	MI		FS Regularization		Hybrid		HPO + FS		HPO	
	nr	cr	nr	cr	nr	cr	nr	cr	nr	cr
SVM (RBF)	55.45% (6.93%)	54.05% (6.07%)	56.95% (6.27%)	57.06% (5.64%)	53.66% (6.79%)	52.74% (6.89%)	55.60% (6.33%)	56.86% (6.67%)	58.46% (4.77%)	58.02% (6.54%)
SVM (linear)	53.09% (6.82%)	53.30% (6.72%)	52.27% (5.49%)	52.77% (6.00%)	54.80% (6.31%)	53.74% (7.02%)	55.53% (6.98%)	56.83% (6.16%)	55.91% (5.68%)	57.10% (6.09%)
SVM (poly)	53.83% (6.74%)	51.28% (7.18%)	53.56% (6.17%)	52.24% (5.93%)	53.93% (6.33%)	52.56% (6.29%)	56.85% (5.95%)	54.41% (6.50%)	54.15% (5.22%)	54.16% (5.84%)
KNN	53.04% (6.95%)	51.11% (6.05%)	53.13% (6.44%)	53.68% (7.01%)	52.93% (7.23%)	52.34% (6.96%)	54.90% (7.01%)	54.44% (5.70%)	52.42% (6.96%)	52.77% (5.11%)
DT	50.89% (6.52%)	51.48% (7.39%)	50.28% (6.38%)	49.24% (6.96%)	51.61% (6.21%)	51.27% (6.79%)	50.67% (6.22%)	51.01% (5.58%)	48.88% (6.97%)	48.77% (5.45%)
NB	53.64% (6.48%)	52.35% (6.06%)	52.90% (6.06%)	52.55% (6.55%)	54.70% (6.85%)	55.51% (7.62%)	-	-	-	-
LDA	52.38% (7.69%)	53.82% (7.26%)	50.89% (6.57%)	50.58% (5.65%)	53.33% (6.85%)	54.25% (7.87%)	-	-	-	-

Note. Mean balanced accuracies (BAC (%)) displayed with standard deviation (SD) appearing in parentheses. Classification results across different FS and HPO pipelines: mutual information (MI) filter, FS with regularization (FS Regularization), filter and wrapper combined (Hybrid), hyperparameter optimization and ANOVA F-test filter (HPO + FS), hyperparameter optimization without additional FS (HPO). nr = no confound regression; cr = age, sex & education regressed from features.

Supplementary Table S15. Classification performance across different feature selection (FS) and hyperparameter optimization (HPO) pipelines for global cognition in comparison to a dummy classifier.

	MI		FS Regularization		Hybrid		HPO + FS		HPO	
	nr	cr	nr	cr	nr	cr	nr	cr	nr	cr
SVM (RBF)	72%	72%	88%	90%	70%	62%	76%	82%	96%	84%
SVM (linear)	56%	62%	62%	58%	82%	66%	78%	90%	86%	86%
SVM (poly)	62%	52%	72%	54%	68%	56%	84%	76%	70%	74%
KNN	56%	46%	56%	70%	64%	58%	74%	70%	60%	60%
DT	54%	54%	50%	42%	62%	54%	52%	60%	48%	42%
NB	62%	64%	62%	56%	74%	64%	-	-	-	-
LDA	58%	68%	52%	62%	64%	64%	-	-	-	-

Note. Percentage (%) of folds for which real models outperform dummy classifier in terms of balanced accuracy (BAC). Classification results across different FS and HPO pipelines: mutual information (MI) filter, FS with regularization (FS Regularization), filter and wrapper combined (Hybrid), hyperparameter optimization and ANOVA F-test filter (HPO + FS), hyperparameter optimization without additional FS (HPO). nr = no confound regression; cr = age, sex & education regressed from features. Colour scheme: green = real model outperforms dummy in ≥ 80% of folds; orange = real model outperforms dummy in 50 - 80% of folds; red = real model outperforms dummy in ≤ 50% of folds.

Supplementary Table S16. Classification performance (sensitivity & specificity) across different feature selection (FS) and hyperparameter optimization (HPO) pipelines for global cognition.

	MI				FS Regularization				Hybrid				FS + HPO				HPO			
	nr		cr		nr		cr		nr		cr		nr		cr		nr		cr	
	Sens	Spec	Sens	Spec	Sens	Spec	Sens	Spec	Sens	Spec	Sens	Spec	Sens	Spec	Sens	Spec	Sens	Spec	Sens	Spec
SVM (RBF)	57.37% (10.82%)	53.53% (9.80%)	55.59% (7.96%)	52.50% (7.92%)	57.85% (10.56%)	56.06% (9.92%)	57.22% (7.56%)	56.90% (7.11%)	54.35% (10.85%)	52.97% (9.24%)	51.97% (8.16%)	53.52% (9.61%)	56.68% (10.55%)	54.52% (10.74%)	56.28% (8.14%)	57.44% (8.71%)	58.61% (9.10%)	58.31% (8.64%)	58.45% (8.25%)	57.59% (8.42%)
SVM (linear)	51.81% (10.15%)	54.37% (10.28%)	51.14% (7.55%)	55.45% (8.49%)	51.02% (9.25%)	53.52% (8.8%)	51.73% (7.01%)	53.82% (7.50%)	51.66% (8.98%)	57.94% (9.88%)	50.42% (7.42%)	57.06% (8.62%)	51.82% (9.67%)	59.24% (10.24%)	52.14% (8.15%)	61.53% (6.10%)	53.45% (7.40%)	58.37% (8.88%)	54.06% (7.88%)	60.14% (7.36%)
SVM (poly)	46.79% (15.53%)	60.86% (13.99%)	36.67% (12.98%)	65.89% (10.43%)	33.70% (13.51%)	73.43% (11.44%)	30.43% (10.83%)	74.04% (7.33%)	47.53% (10.37%)	60.33% (11.39%)	46.02% (9.85%)	59.09% (10.01%)	53.89% (12.52%)	59.81% (11.23%)	48.15% (17.67%)	60.66% (14.59%)	61.86% (11.35%)	46.43% (13.06%)	64.24% (7.37%)	44.08% (8.72%)
KNN	59.46% (10.66%)	46.63% (9.24%)	55.65% (9.50%)	46.57% (9.65%)	61.23% (9.71%)	45.02% (9.43%)	58.91% (9.91%)	48.44% (10.31%)	56.20% (11.15%)	49.66% (11.06%)	56.53% (10.23%)	48.14% (9.08%)	67.65% (10.36%)	42.15% (8.74%)	65.08% (6.30%)	43.80% (8.76%)	61.61% (11.62%)	43.24% (8.64%)	60.16% (9.20%)	45.39% (8.98%)
DT	50.79% (9.53%)	50.98% (10.51%)	49.34% (9.10%)	53.61% (11.53%)	49.97% (8.81%)	50.59% (9.5%)	48.77% (10.01%)	49.72% (9.56%)	51.87% (10.21%)	51.36% (9.99%)	50.42% (9.08%)	52.12% (8.91%)	55.61% (14.40%)	45.73% (11.59%)	52.05% (8.90%)	49.98% (9.75%)	52.94% (15.79%)	44.81% (15.28%)	47.87% (11.01%)	49.66% (12.25%)
NB	44.10% (10.26%)	63.18% (8.77%)	42.78% (8.05%)	61.92% (7.55%)	43.10% (10.55%)	62.70% (8.65%)	43.26% (7.91%)	61.84% (6.96%)	50.03% (9.73%)	59.36% (10.06%)	50.58% (9.17%)	60.44% (8.03%)	-	-	-	-	-	-	-	-
LDA	50.40% (9.81%)	54.36% (10.59%)	52.35% (7.94%)	55.28% (8.68%)	49.87% (9.93%)	51.91% (8.95%)	49.72% (8.38%)	51.44% (6.82%)	50.82% (11.19%)	55.83% (8.71%)	52.27% (8.64%)	56.24% (8.93%)	-	-	-	-	-	-	-	-

Note. Mean sensitivity (Sens, %) and specificity (Spec, %) displayed with standard deviation (SD) appearing in parentheses. Classification results across different FS and HPO pipelines: mutual information (MI) filter, FS with regularization (FS Regularization), filter and wrapper combined (Hybrid), hyperparameter optimization and ANOVA F-test filter (HPO + FS), hyperparameter optimization without additional FS (HPO). nr = no confound regression; cr = age, sex & education regressed from features.

Supplementary Table S17. Classification performance estimates for global cognition across feature sets for pure and univariate FS (ANOVA F-test) pipeline in the matched and unmatched sample.

		Matched Sample								Unmatched Sample							
		21		421		1200		1621		21		421		1200		1621	
		nr	cr	nr	cr	nr	cr	nr	cr	nr	cr	nr	cr	nr	cr	nr	cr
Pure	SVM	51.75%	50.58%	57.41%	58.33%	55.46%	55.95%	55.65%	56.14%	56.91%	51.76%	59.31%	56.81%	58.30%	54.84%	58.72%	54.86%
	(RBF)	(6.09%)	(6.54%)	(6.21%)	(6.02%)	(5.63%)	(6.68%)	(6.11%)	(6.69%)	(5.18%)	(5.41%)	(5.35%)	(5.18%)	(5.18%)	(5.37%)	(5.24%)	(5.39%)
	SVM	54.77%	53.75%	52.77%	52.20%	51.23%	50.47%	50.92%	49.36%	57.77%	52.48%	56.60%	53.30%	55.34%	52.33%	55.60%	53.10%
	(linear)	(7.03%)	(6.15%)	(6.69%)	(6.68%)	(6.83%)	(7.54%)	(6.57%)	(7.15%)	(4.65%)	(6.24%)	(4.64%)	(5.54%)	(5.42%)	(4.71%)	(4.91%)	(4.97%)
	SVM	52.78%	52.06%	53.25%	51.96%	52.98%	53.31%	52.71%	51.77%	54.60%	51.95%	56.00%	50.91%	56.88%	51.10%	56.82%	51.36%
	(poly)	(7.14%)	(6.20%)	(5.50%)	(6.43%)	(5.27%)	(6.36%)	(5.50%)	(6.78%)	(4.91%)	(5.76%)	(3.87%)	(4.30%)	(4.15%)	(3.54%)	(4.50%)	(3.79%)
	KNN	49.85%	49.30%	52.90%	52.05%	54.67%	55.40%	54.83%	54.09%	51.07%	50.94%	54.72%	53.55%	53.72%	52.96%	54.36%	53.70%
		(6.54%)	(5.45%)	(6.43%)	(7.39%)	(6.88%)	(7.63%)	(6.15%)	(7.64%)	(6.70%)	(5.03%)	(5.32%)	(5.59%)	(4.68%)	(5.02%)	(5.15%)	(5.16%)
	DT	49.68%	48.42%	50.13%	48.68%	48.96%	50.41%	49.08%	49.32%	52.88%	50.03%	53.10%	53.24%	51.52%	52.07%	52.59%	53.63%
		(7.38%)	(6.54%)	(7.62%)	(6.38%)	(6.54%)	(6.51%)	(7.09%)	(7.00%)	(5.43%)	(6.09%)	(5.21%)	(5.78%)	(4.93%)	(5.55%)	(5.5%)	(5.92%)
	NB	51.32%	50.50%	52.94%	52.24%	50.99%	50.34%	51.93%	50.96%	55.00%	51.51%	55.25%	51.98%	56.13%	53.68%	55.98%	53.09%
		(6.10%)	(6.72%)	(6.17%)	(6.71%)	(5.52%)	(5.58%)	(5.67%)	(6.06%)	(4.86%)	(5.63%)	(5.03%)	(5.08%)	(5.02%)	(4.52%)	(5.01%)	(4.62%)
	LDA	54.04%	52.48%	51.04%	50.37%	51.63%	49.19%	51.82%	49.23%	57.24%	52.16%	55.51%	52.57%	51.95%	53.08%	52.73%	52.51%
		(6.58%)	(7.20%)	(7.76%)	(7.69%)	(7.21%)	(6.74%)	(6.64%)	(6.60%)	(4.78%)	(5.79%)	(5.32%)	(6.31%)	(5.4%)	(4.24%)	(4.72%)	(5.20%)
FS	SVM	49.71%	48.63%	57.13%	57.59%	54.84%	55.55%	55.65%	56.52%	53.87%	50.00%	55.99%	55.20%	56.77%	53.33%	55.95%	53.83%
	(RBF)	(6.72%)	(7.01%)	(7.08%)	(7.10%)	(7.14%)	(7.44%)	(7.65%)	(6.97%)	(5.03%)	(6.01%)	(5.05%)	(5.34%)	(4.86%)	(5.75%)	(5.34%)	(5.94%)
	SVM	52.10%	51.32%	55.87%	56.33%	54.21%	54.27%	54.83%	55.14%	54.66%	52.14%	55.44%	51.26%	56.33%	50.57%	56.31%	50.74%
	(linear)	(6.84%)	(7.96%)	(7.57%)	(7.21%)	(6.52%)	(6.34%)	(7.29%)	(6.80%)	(4.61%)	(4.55%)	(4.32%)	(4.86%)	(4.90%)	(5.34%)	(5.26%)	(5.48%)
	SVM	50.60%	51.05%	56.09%	54.78%	53.41%	53.86%	54.84%	53.70%	53.48%	50.93%	53.66%	52.19%	55.08%	53.27%	54.31%	53.23%
	(poly)	(4.53%)	(5.17%)	(6.21%)	(6.30%)	(6.69%)	(5.89%)	(7.74%)	(6.62%)	(3.65%)	(3.10%)	(4.02%)	(4.60%)	(4.26%)	(5.27%)	(3.97%)	(5.49%)
	KNN	50.96%	51.30%	53.74%	54.57%	53.80%	53.45%	52.83%	56.26%	49.33%	50.46%	53.82%	54.49%	52.82%	52.13%	51.99%	52.07%
		(7.13%)	(6.14%)	(6.31%)	(6.93%)	(6.70%)	(6.01%)	(5.72%)	(7.43%)	(4.99%)	(5.41%)	(4.48%)	(5.18%)	(5.10%)	(4.96%)	(5.63%)	(4.49%)
	DT	50.95%	50.50%	51.34%	51.62%	49.35%	51.01%	50.54%	51.39%	50.19%	50.13%	53.06%	52.86%	50.14%	50.91%	50.10%	50.60%
		(7.60%)	(6.72%)	(6.65%)	(6.79%)	(8.71%)	(6.92%)	(5.68%)	(7.90%)	(4.50%)	(6.59%)	(5.30%)	(5.59%)	(5.24%)	(4.90%)	(5.10%)	(4.72%)
	NB	50.99%	50.36%	52.98%	52.47%	51.43%	51.54%	52.81%	51.50%	54.31%	51.27%	55.76%	53.46%	56.16%	53.36%	55.95%	52.96%
		(6.59%)	(6.95%)	(6.62%)	(5.99%)	(5.95%)	(6.91%)	(6.02%)	(6.94%)	(4.88%)	(4.74%)	(3.98%)	(4.63%)	(4.37%)	(5.01%)	(4.09%)	(4.37%)
	LDA	52.34%	51.13%	54.72%	56.13%	54.29%	54.51%	55.71%	54.35%	54.48%	52.04%	55.19%	50.61%	56.55%	50.04%	54.59%	51.86%
		(7.28%)	(7.68%)	(7.77%)	(6.35%)	(6.81%)	(6.45%)	(6.36%)	(6.78%)	(5.10%)	(5.63%)	(4.69%)	(4.91%)	(4.82%)	(5.07%)	(5.89%)	(5.68%)

Note. Mean balanced accuracies (BAC (%)) displayed with standard deviation (SD) appearing in parentheses. nr = no confound regression; cr = age, sex & education regressed from features.

Supplementary Table S18. Classification performance estimates for global cognition across feature sets for pure and univariate FS (ANOVA F-test) pipeline in the matched and unmatched sample in comparison to a dummy classifier.

		Matched Sample								Unmatched Sample							
		21		421		1200		1621		21		421		1200		1621	
		nr	cr	nr	cr	nr	cr	nr	cr	nr	cr	nr	cr	nr	cr	nr	cr
Pure	SVM (RBF)	58%	50%	86%	94%	78%	76%	80%	82%	92%	62%	98%	94%	92%	82%	92%	84%
	SVM(linear)	78%	68%	58%	52%	50%	44%	52%	40%	94%	60%	86%	68%	82%	66%	86%	64%
	SVM (poly)	60%	60%	66%	54%	58%	64%	56%	54%	78%	62%	92%	58%	94%	60%	94%	58%
	KNN	46%	46%	66%	60%	76%	76%	76%	76%	58%	50%	66%	66%	80%	70%	82%	72%
	DT	44%	38%	46%	40%	42%	38%	52%	40%	66%	48%	64%	64%	68%	58%	66%	64%
	NB	52%	52%	58%	58%	46%	48%	50%	56%	88%	64%	88%	64%	92%	78%	88%	74%
	LDA	76%	60%	62%	52%	56%	46%	52%	38%	96%	60%	88%	70%	56%	80%	76%	72%
FS	SVM (RBF)	40%	34%	82%	82%	78%	68%	76%	74%	76%	42%	84%	76%	92%	68%	82%	72%
	SVM (linear)	56%	54%	80%	78%	76%	74%	66%	74%	86%	58%	92%	60%	86%	50%	88%	54%
	SVM (poly)	54%	56%	80%	72%	72%	68%	66%	68%	78%	64%	78%	68%	88%	76%	80%	76%
	KNN	48%	54%	64%	68%	74%	56%	62%	78%	44%	52%	64%	66%	68%	72%	66%	62%
	DT	52%	52%	52%	54%	42%	58%	50%	52%	58%	54%	58%	66%	56%	54%	46%	62%
	NB	52%	50%	62%	58%	60%	52%	62%	52%	82%	50%	90%	76%	92%	68%	94%	68%
	LDA	54%	58%	76%	86%	72%	76%	84%	74%	84%	56%	94%	62%	88%	44%	78%	62%

Note. Percentage (%) of folds for which real models outperform dummy classifier in terms of balanced accuracy (BAC). nr = no confound regression; cr = age, sex & education regressed from features. Colour scheme: green = real model outperforms dummy in ≥ 80% of folds; orange = real model outperforms dummy in 50 - 80% of folds; red = real model outperforms dummy in ≤ 50% of folds.

Supplementary Table S19. Classification performance (sensitivity & specificity) estimates for global cognition across feature sets for pure and univariate FS (ANOVA F-test) pipeline in the matched sample.

		21				421				1200				1621			
		nr		cr		nr		cr		nr		cr		nr		cr	
		Sens	Spec	Sens	Spec	Sens	Spec	Sens	Spec	Sens	Spec	Sens	Spec	Sens	Spec	Sens	Spec
Pure	SVM	56.31%	47.19%	55.05%	46.12%	59.08%	55.74%	58.92%	57.74%	60.79%	50.13%	61.94%	49.96%	60.02%	51.27%	60.70%	51.58%
	(RBF)	(9.98%)	(10.58%)	(9.32%)	(9.52%)	(10.03%)	(9.85%)	(8.06%)	(7.60%)	(10.28%)	(9.62%)	(9.45%)	(8.06%)	(10.94%)	(9.34%)	(9.04%)	(8.42%)
	SVM	51.68%	57.85%	50.82%	56.67%	52.50%	53.05%	51.50%	52.90%	51.90%	50.57%	50.50%	50.43%	51.42%	50.42%	49.66%	49.05%
	(linear)	(10.69%)	(11.03%)	(7.11%)	(7.28%)	(11.21%)	(10.5%)	(7.43%)	(8.78%)	(9.18%)	(11.44%)	(7.87%)	(9.22%)	(9.12%)	(11.94%)	(7.47%)	(8.72%)
	SVM	52.55%	53.01%	54.84%	49.29%	32.06%	74.44%	29.80%	74.13%	22.70%	83.26%	23.92%	82.70%	25.33%	80.09%	24.93%	78.61%
	(poly)	(12.05%)	(11.75%)	(10.61%)	(9.52%)	(13.05%)	(10.15%)	(10.90%)	(8.03%)	(13.59%)	(9.35%)	(12.63%)	(9.37%)	(13.85%)	(10.20%)	(13.23%)	(9.39%)
	KNN	50.27%	49.43%	48.62%	49.97%	62.31%	43.48%	56.44%	47.66%	60.99%	48.35%	58.67%	52.13%	61.93%	47.73%	59.13%	49.04%
		(9.64%)	(9.94%)	(8.85%)	(8.35%)	(9.03%)	(9.46%)	(9.63%)	(9.98%)	(10.35%)	(10.6%)	(12.36%)	(8.70%)	(9.83%)	(9.38%)	(11.45%)	(9.42%)
	DT	49.44%	49.92%	48.48%	48.35%	48.89%	51.37%	47.19%	50.17%	48.80%	49.11%	50.33%	50.50%	49.62%	48.53%	49.71%	48.93%
		(9.87%)	(10.89%)	(11.23%)	(10.94%)	(9.29%)	(11.61%)	(10.36%)	(9.43%)	(10.26%)	(9.53%)	(11.74%)	(9.19%)	(10.75%)	(9.92%)	(9.51%)	(9.22%)
	NB	53.75%	48.88%	51.66%	49.33%	43.56%	62.32%	42.63%	61.84%	42.14%	59.84%	41.91%	58.76%	42.70%	61.15%	41.92%	60.00%
		(11.76%)	(11.22%)	(10.17%)	(9.79%)	(9.92%)	(8.92%)	(8.33%)	(6.91%)	(10.69%)	(8.68%)	(8.44%)	(7.47%)	(9.85%)	(8.46%)	(8.52%)	(6.80%)
	LDA	50.30%	57.78%	50.75%	54.21%	50.03%	52.06%	49.04%	51.70%	51.35%	51.91%	49.12%	49.26%	50.49%	53.14%	49.74%	48.72%
		(10.99%)	(10.83%)	(8.77%)	(8.30%)	(11.29%)	(9.15%)	(8.02%)	(9.08%)	(10.09%)	(12.22%)	(7.99%)	(8.25%)	(9.27%)	(10.72%)	(7.66%)	(8.06%)
FS	SVM	57.02%	42.39%	48.52%	48.75%	57.52%	56.74%	56.20%	58.98%	56.24%	53.45%	57.00%	54.11%	57.70%	53.59%	56.92%	56.12%
	(RBF)	(15.04%)	(13.36%)	(15.00%)	(14.48%)	(10.68%)	(9.95%)	(8.60%)	(8.76%)	(10.28%)	(11.40%)	(8.62%)	(8.98%)	(10.29%)	(12.33%)	(8.29%)	(7.94%)
	SVM	47.44%	56.77%	46.03%	56.60%	54.66%	57.08%	53.12%	59.54%	55.21%	53.21%	55.52%	53.02%	55.32%	54.34%	54.59%	55.68%
	(linear)	(11.00%)	(9.60%)	(10.43%)	(8.43%)	(11.02%)	(9.87%)	(8.32%)	(7.78%)	(10.22%)	(9.87%)	(6.69%)	(8.10%)	(9.58%)	(10.71%)	(8.20%)	(8.39%)
	SVM	15.38%	85.81%	18.76%	83.34%	37.77%	74.42%	35.52%	74.03%	63.47%	43.35%	63.46%	44.26%	53.14%	56.54%	51.81%	55.60%
	(poly)	(11.99%)	(14.39%)	(19.18%)	(21.00%)	(10.83%)	(8.39%)	(11.29%)	(8.17%)	(13.98%)	(18.66%)	(13.79%)	(17.17%)	(14.54%)	(17.39%)	(15.46%)	(17.07%)
	KNN	52.22%	49.70%	51.35%	51.26%	63.86%	43.62%	64.68%	44.47%	73.69%	33.90%	70.90%	35.99%	71.51%	34.15%	73.82%	38.69%
		(9.68%)	(9.34%)	(7.13%)	(8.37%)	(9.13%)	(8.84%)	(10.67%)	(7.88%)	(9.58%)	(11.54%)	(7.21%)	(9.67%)	(8.50%)	(10.00%)	(8.11%)	(11.19%)
	DT	50.43%	51.47%	50.35%	50.66%	51.18%	51.50%	50.96%	52.28%	50.49%	48.20%	50.96%	51.05%	50.79%	50.30%	51.56%	51.22%
		(10.94%)	(10.76%)	(8.04%)	(10.54%)	(10.47%)	(8.89%)	(9.05%)	(9.38%)	(11.39%)	(11.28%)	(10.34%)	(9.65%)	(9.57%)	(11.82%)	(10.38%)	(9.87%)
	NB	42.27%	59.70%	41.57%	59.16%	44.25%	61.70%	44.32%	60.62%	45.86%	56.99%	46.71%	56.37%	45.47%	60.15%	44.31%	58.68%
		(11.02%)	(10.11%)	(9.82%)	(9.19%)	(11.05%)	(9.94%)	(7.46%)	(6.83%)	(11.43%)	(9.59%)	(9.36%)	(8.03%)	(11.02%)	(9.09%)	(9.65%)	(7.88%)
	LDA	50.75%	53.92%	48.73%	53.52%	54.13%	55.30%	53.74%	58.53%	56.15%	52.44%	54.99%	54.03%	55.30%	56.12%	53.67%	55.03%
		(10.52%)	(9.97%)	(10.30%)	(7.31%)	(10.58%)	(10.70%)	(8.19%)	(6.17%)	(10.94%)	(9.51%)	(6.43%)	(8.21%)	(9.49%)	(9.29%)	(8.40%)	(7.98%)

Note. Mean sensitivity (Sens, %) and specificity (Spec, %) displayed with standard deviation (SD) appearing in parentheses. nr = no confound regression; cr = age, sex & education regressed from features.

Supplementary Table S20. Classification performance (sensitivity & specificity) estimates for global cognition across feature sets for pure and univariate FS (ANOVA F-test) pipeline in the unmatched sample.

		21				421				1200				1621			
		nr		cr		nr		cr		nr		cr		nr		cr	
		Sens	Spec	Sens	Spec	Sens	Spec	Sens	Spec	Sens	Spec	Sens	Spec	Sens	Spec	Sens	Spec
Pure	SVM	59.55%	54.27%	53.23%	50.29%	61.07%	57.54%	56.22%	57.39%	59.95%	56.64%	58.10%	51.59%	60.19%	57.24%	57.01%	52.71%
	(RBF)	(7.94%)	(9.17%)	(6.80%)	(7.98%)	(8.51%)	(7.05%)	(6.94%)	(7.18%)	(8.82%)	(6.75%)	(7.35%)	(6.37%)	(8.56%)	(6.56%)	(6.69%)	(6.86%)
	SVM	52.29%	63.24%	43.99%	60.97%	57.15%	56.05%	52.90%	53.71%	56.21%	54.46%	52.87%	51.79%	56.31%	54.90%	53.33%	52.87%
	(linear)	(7.83%)	(7.32%)	(7.19%)	(7.29%)	(7.08%)	(7.32%)	(7.28%)	(6.19%)	(7.34%)	(7.95%)	(5.51%)	(6.03%)	(6.54%)	(7.32%)	(6.18%)	(5.96%)
	SVM	47.79%	61.42%	43.54%	60.36%	29.55%	82.46%	20.21%	81.62%	28.09%	85.67%	15.56%	86.65%	28.77%	84.87%	17.86%	84.87%
	(poly)	(8.68%)	(8.60%)	(7.31%)	(9.50%)	(7.63%)	(6.10%)	(5.62%)	(6.24%)	(7.77%)	(5.79%)	(6.03%)	(5.99%)	(7.78%)	(5.54%)	(6.56%)	(5.84%)
	KNN	50.13%	52.01%	47.52%	54.37%	67.42%	42.03%	52.76%	54.34%	67.81%	39.64%	58.54%	47.38%	68.10%	40.62%	56.13%	51.27%
		(9.57%)	(9.25%)	(7.11%)	(7.51%)	(7.67%)	(7.17%)	(8.59%)	(9.00%)	(7.95%)	(8.63%)	(7.82%)	(7.17%)	(7.37%)	(8.25%)	(6.82%)	(8.24%)
	DT	53.02%	52.73%	48.65%	51.41%	54.65%	51.55%	54.60%	51.88%	50.41%	52.62%	51.29%	52.84%	51.40%	53.77%	52.41%	54.86%
		(7.23%)	(8.28%)	(7.74%)	(9.07%)	(7.45%)	(7.39%)	(8.74%)	(6.53%)	(8.14%)	(7.3%)	(6.79%)	(7.92%)	(7.37%)	(7.00%)	(7.86%)	(7.70%)
	NB	51.66%	58.33%	43.47%	59.55%	46.25%	64.24%	38.64%	65.32%	46.10%	66.15%	39.52%	67.84%	46.01%	65.95%	38.63%	67.54%
		(8.69%)	(7.60%)	(8.55%)	(8.64%)	(7.74%)	(7.5%)	(6.06%)	(5.76%)	(7.87%)	(6.33%)	(5.40%)	(5.56%)	(7.56%)	(6.63%)	(5.56%)	(5.15%)
	LDA	55.24%	59.24%	49.61%	54.70%	56.06%	54.96%	52.53%	52.61%	52.12%	51.77%	52.83%	53.34%	54.08%	51.38%	51.78%	53.25%
		(7.78%)	(7.44%)	(7.60%)	(5.57%)	(7.14%)	(7.5%)	(6.88%)	(6.91%)	(6.55%)	(8.56%)	(5.16%)	(5.61%)	(6.62%)	(7.63%)	(5.85%)	(6.87%)
FS	SVM	51.96%	55.78%	43.71%	56.29%	54.06%	57.92%	52.63%	57.77%	60.16%	53.39%	56.12%	50.54%	57.64%	54.26%	55.49%	52.17%
	(RBF)	(9.11%)	(8.57%)	(10.34%)	(12.42%)	(7.88%)	(7.79%)	(7.08%)	(7.72%)	(8.20%)	(7.74%)	(7.20%)	(6.84%)	(9.07%)	(7.85%)	(6.38%)	(7.32%)
	SVM	45.67%	63.65%	37.86%	66.42%	50.54%	60.34%	47.65%	54.88%	58.01%	54.66%	51.18%	49.96%	58.25%	54.37%	52.24%	49.24%
	(linear)	(8.83%)	(7.54%)	(6.49%)	(8.10%)	(8.23%)	(6.97%)	(7.49%)	(5.71%)	(6.89%)	(6.52%)	(6.21%)	(6.82%)	(7.49%)	(7.20%)	(6.57%)	(6.06%)
	SVM	20.32%	86.64%	25.59%	76.27%	24.82%	82.51%	22.96%	81.41%	34.81%	75.36%	48.87%	57.68%	30.03%	78.60%	39.10%	67.37%
	(poly)	(14.93%)	(15.11%)	(32.69%)	(34.41%)	(7.26%)	(6.06%)	(5.71%)	(6.27%)	(11.17%)	(9.62%)	(15.56%)	(15.73%)	(9.74%)	(6.80%)	(14.09%)	(13.20%)
	KNN	49.30%	49.36%	50.63%	50.30%	66.47%	41.18%	57.94%	51.03%	67.91%	37.72%	64.16%	40.10%	67.63%	36.35%	63.29%	40.85%
		(8.52%)	(7.99%)	(7.20%)	(7.96%)	(7.22%)	(7.14%)	(6.20%)	(8.72%)	(7.90%)	(7.19%)	(7.44%)	(7.64%)	(8.16%)	(7.42%)	(6.11%)	(8.11%)
	DT	50.48%	49.90%	51.01%	49.26%	53.13%	52.98%	52.67%	53.05%	51.16%	49.11%	49.90%	51.92%	51.24%	48.96%	50.13%	51.08%
		(8.05%)	(8.22%)	(8.82%)	(8.83%)	(7.64%)	(8.02%)	(8.00%)	(8.46%)	(7.39%)	(7.35%)	(8.77%)	(8.58%)	(7.30%)	(7.21%)	(8.45%)	(7.58%)
	NB	45.43%	63.20%	37.22%	65.32%	45.56%	65.95%	39.23%	67.69%	45.32%	67.00%	41.05%	65.66%	45.36%	66.54%	38.93%	66.99%
		(8.53%)	(8.97%)	(7.61%)	(9.68%)	(6.19%)	(6.95%)	(6.23%)	(5.16%)	(7.07%)	(6.99%)	(6.74%)	(5.51%)	(6.71%)	(6.58%)	(5.78%)	(5.17%)
	LDA	51.27%	57.69%	47.67%	56.41%	51.47%	58.92%	48.86%	52.36%	56.78%	56.33%	50.28%	49.80%	55.91%	53.28%	52.14%	51.58%
		(8.45%)	(7.00%)	(5.94%)	(7.14%)	(8.05%)	(6.31%)	(6.38%)	(5.51%)	(5.84%)	(7.32%)	(5.89%)	(5.98%)	(7.61%)	(7.59%)	(6.36%)	(6.49%)

Note. Mean sensitivity (Sens, %) and specificity (Spec, %) displayed with standard deviation (SD) appearing in parentheses. nr = no confound regression; cr = age, sex & education regressed from features.

Regression

Supplementary Table S21. Regression performance for global & domain-specific cognition across pipeline configurations in feature set 421.

		COGNITIVE COMPOSITE						NON-VERBAL MEMORY & EXECUTIVE						VERBAL MEMORY & LANGUAGE					
		MAE			R^2			MAE			R^2			MAE			R^2		
		nr	nr-cr	cr-cr	nr	nr-cr	cr-cr	nr	nr-cr	cr-cr	nr	nr-cr	cr-cr	nr	nr-cr	cr-cr	nr	nr-cr	cr-cr
Pure	SVR	0.76	0.79	0.79	0.06	-0.01	0.00	0.76	0.79	0.79	0.03	-0.02	-0.01	0.81	0.82	0.82	-0.03	-0.03	-0.03
		(0.06)	(0.03)	(0.03)	(0.06)	(0.04)	(0.04)	(0.06)	(0.03)	(0.03)	(0.07)	(0.05)	(0.06)	(0.06)	(0.02)	(0.02)	(0.07)	(0.05)	(0.04)
	RVR	0.77	0.82	0.81	0.01	-0.08	-0.06	0.79	0.83	0.83	-0.03	-0.10	-0.08	0.84	0.83	0.84	-0.10	-0.08	-0.09
		(0.06)	(0.04)	(0.03)	(0.08)	(0.07)	(0.06)	(0.06)	(0.04)	(0.04)	(0.08)	(0.07)	(0.07)	(0.06)	(0.04)	(0.03)	(0.10)	(0.07)	(0.06)
	Elastic	0.80	0.79	0.79	-0.01	0.00	0.00	0.79	0.79	0.79	-0.01	0.00	0.00	0.81	0.81	0.81	-0.02	0.00	0.00
	Net	(0.07)	(0.03)	(0.03)	(0.02)	(0.00)	(0.00)	(0.07)	(0.03)	(0.03)	(0.02)	(0.00)	(0.00)	(0.06)	(0.02)	(0.02)	(0.03)	(0.00)	(0.00)
	Lasso	0.80	0.79	0.79	-0.01	0.00	0.00	0.79	0.79	0.79	-0.01	0.00	0.00	0.81	0.81	0.81	-0.02	0.00	0.00
		(0.07)	(0.03)	(0.03)	(0.02)	(0.00)	(0.00)	(0.07)	(0.03)	(0.03)	(0.02)	(0.00)	(0.00)	(0.06)	(0.02)	(0.02)	(0.03)	(0.00)	(0.00)
	Ridge	1.09	1.13	1.14	-0.92	-1.11	-1.12	1.11	1.14	1.14	-0.99	-1.09	-1.10	1.13	1.16	1.16	-1.12	-1.17	-1.18
	(λ=1)	(0.09)	(0.09)	(0.09)	(0.34)	(0.32)	(0.31)	(0.09)	(0.09)	(0.09)	(0.42)	(0.29)	(0.28)	(0.10)	(0.09)	(0.09)	(0.39)	(0.33)	(0.32)
	Ridge	0.78	0.82	0.82	-0.02	-0.08	-0.08	0.79	0.82	0.82	-0.04	-0.09	-0.09	0.83	0.84	0.83	-0.1	-0.11	-0.11
	(adj. λ)	(0.06)	(0.04)	(0.04)	(0.09)	(0.07)	(0.08)	(0.06)	(0.04)	(0.04)	(0.09)	(0.08)	(0.09)	(0.06)	(0.03)	(0.03)	(0.1)	(0.07)	(0.07)
FS &	SVR	0.78	0.79	0.79	0.00	0.00	0.00	0.77	0.79	0.79	0.02	0.00	0.00	0.80	0.81	0.81	-0.02	0.00	-0.01
HPO		(0.06)	(0.03)	(0.03)	(0.05)	(0.01)	(0.02)	(0.07)	(0.03)	(0.03)	(0.04)	(0.02)	(0.02)	(0.06)	(0.02)	(0.02)	(0.05)	(0.01)	(0.01)
	RVR	0.81	0.84	0.84	-0.05	-0.14	-0.13	0.80	0.83	0.79	-0.05	-0.10	0.00	0.85	0.86	0.82	-0.13	-0.15	-0.02
		(0.06)	(0.04)	(0.04)	(0.09)	(0.08)	(0.08)	(0.06)	(0.03)	(0.03)	(0.07)	(0.07)	(0.03)	(0.06)	(0.04)	(0.03)	(0.11)	(0.07)	(0.02)
	Elastic	0.77	0.79	0.79	0.05	0.00	0.00	0.76	0.79	0.79	0.04	0.00	0.00	0.80	0.81	0.81	0.00	0.00	0.00
	Net	(0.06)	(0.03)	(0.03)	(0.06)	(0.02)	(0.02)	(0.06)	(0.03)	(0.03)	(0.05)	(0.03)	(0.03)	(0.06)	(0.02)	(0.02)	(0.04)	(0.01)	(0.01)
	Lasso	0.78	0.79	0.79	0.03	0.00	0.00	0.77	0.79	0.79	0.02	0.00	0.00	0.81	0.81	0.81	-0.02	0.00	0.00
		(0.06)	(0.03)	(0.03)	(0.03)	(0.01)	(0.01)	(0.07)	(0.03)	(0.03)	(0.03)	(0.01)	(0.01)	(0.06)	(0.02)	(0.02)	(0.03)	(0.00)	(0.00)
	Ridge	0.78	0.79	0.79	0.03	0.00	0.00	0.77	0.79	0.79	0.03	0.00	0.00	0.80	0.81	0.81	-0.02	0.00	0.00
		(0.06)	(0.03)	(0.03)	(0.04)	(0.01)	(0.01)	(0.07)	(0.03)	(0.03)	(0.04)	(0.01)	(0.02)	(0.06)	(0.02)	(0.02)	(0.04)	(0.01)	(0.01)

Note. Average mean absolute error (MAE) and coefficient of determination (R^2) displayed with standard deviation (SD) appearing in parentheses. Pure = no feature selection; FS+HPO = feature selection and hyperparameter optimization; Ridge(adj. λ): default values manually adjusted; nr = no confound regression; nr-cr = age, sex & education regressed from target only; cr-cr = age, sex & education regressed from target and features.

Supplementary Table S22. Regression performance for global & domain-specific cognition across pipeline configurations in feature set 421 in comparison to dummy regressor.

		COGNITIVE COMPOSITE			NON-VERBAL MEMORY & EXECUTIVE			VERBAL MEMORY & LANGUAGE		
		nr	nr-cr	cr-cr	nr	nr-cr	cr-cr	nr	nr-cr	cr-cr
Pure	SVR	86%	46%	54%	76%	48%	50%	52%	30%	50%
	RVR	60%	12%	14%	42%	8%	10%	14%	12%	10%
	Elastic Net	22%	0%	0%	16%	8%	8%	32%	4%	4%
	Lasso	22%	0%	0%	16%	8%	8%	32%	4%	4%
	Ridge (λ=1)	0%	0%	0%	0%	0%	0%	0%	0%	0%
	Ridge (adj. λ)	48%	14%	10%	42%	18%	16%	18%	6%	16%
FS & HPO	SVR	60%	50%	46%	70%	54%	60%	62%	10%	10%
	RVR	42%	8%	4%	24%	6%	6%	14%	0%	0%
	Elastic Net	90%	56%	46%	84%	76%	70%	68%	2%	2%
	Lasso	98%	40%	42%	88%	38%	40%	24%	4%	4%
	Ridge	76%	60%	42%	84%	72%	70%	60%	50%	48%

Note. Percentage (%) of folds for which real models outperform dummy regressor in terms of coefficient of determination (R^2). Pure = no feature selection; FS+HPO = feature selection and hyperparameter optimization; Ridge(adj. λ): default values manually adjusted; nr = no confound regression; nr-cr = age, sex & education regressed from target only; cr-cr = age, sex & education regressed from target and features. Colour scheme: green = real model outperforms dummy in ≥ 80% of folds; orange = real model outperforms dummy in 50 - 80% of folds; red = real model outperforms dummy in ≤ 50% of folds.

Supplementary Table S23. Regression performance across feature sets and pipeline configurations for global cognition.

		421						1621					
		MAE			R^2			MAE			R^2		
		nr	nr-cr	cr-cr	nr	nr-cr	cr-cr	nr	nr-cr	cr-cr	nr	nr-cr	cr-cr
Pure	SVR	0.76 (0.06)	0.79 (0.03)	0.79 (0.03)	0.06 (0.06)	-0.01 (0.04)	0.00 (0.04)	0.75 (0.06)	0.79 (0.03)	0.79 (0.03)	0.07 (0.06)	0.00 (0.04)	0.00 (0.03)
	RVR	0.77 (0.06)	0.82 (0.04)	0.81 (0.03)	0.01 (0.08)	-0.08 (0.07)	-0.06 (0.06)	0.76 (0.06)	0.80 (0.03)	0.80 (0.03)	0.05 (0.07)	-0.03 (0.05)	-0.02 (0.05)
	Elastic Net	0.80 (0.07)	0.79 (0.03)	0.79 (0.03)	-0.01 (0.02)	0.00 (0.00)	0.00 (0.00)	0.80 (0.07)	0.79 (0.03)	0.79 (0.03)	-0.01 (0.02)	0.00 (0.00)	0.00 (0.00)
	Lasso	0.80 (0.07)	0.79 (0.03)	0.79 (0.03)	-0.01 (0.02)	0.00 (0.00)	0.00 (0.00)	0.80 (0.07)	0.79 (0.03)	0.79 (0.03)	-0.01 (0.02)	0.00 (0.00)	0.00 (0.00)
	Ridge ($\lambda = 1$)	1.09 (0.09)	1.13 (0.09)	1.14 (0.09)	-0.92 (0.34)	-1.11 (0.32)	-1.12 (0.31)	1.28 (0.08)	1.42 (0.09)	1.40 (0.10)	-1.72 (0.50)	-2.13 (0.40)	-2.09 (0.41)
	Ridge (adj. λ)	0.78 (0.06)	0.82 (0.04)	0.82 (0.04)	-0.02 (0.09)	-0.08 (0.07)	-0.08 (0.08)	0.85 (0.07)	0.90 (0.04)	0.90 (0.04)	-0.19 (0.15)	-0.30 (0.13)	-0.31 (0.14)
FS & HPO	SVR	0.78 (0.06)	0.79 (0.03)	0.79 (0.03)	0.00 (0.05)	0.00 (0.01)	0.00 (0.02)	0.77 (0.06)	0.79 (0.03)	0.79 (0.03)	0.02 (0.06)	0.00 (0.02)	-0.01 (0.02)
	RVR	0.81 (0.06)	0.84 (0.04)	0.84 (0.04)	-0.05 (0.09)	-0.14 (0.08)	-0.13 (0.08)	0.82 (0.07)	0.84 (0.05)	0.83 (0.04)	-0.08 (0.12)	-0.13 (0.10)	-0.11 (0.10)
	Elastic Net	0.77 (0.06)	0.79 (0.03)	0.79 (0.03)	0.05 (0.06)	0.00 (0.02)	0.00 (0.02)	0.76 (0.06)	0.79 (0.03)	0.79 (0.03)	0.05 (0.06)	0.00 (0.02)	0.00 (0.02)
	Lasso	0.78 (0.06)	0.79 (0.03)	0.79 (0.03)	0.03 (0.03)	0.00 (0.01)	0.00 (0.01)	0.78 (0.06)	0.79 (0.03)	0.79 (0.03)	0.04 (0.04)	0.01 (0.01)	0.01 (0.01)
	Ridge	0.78 (0.06)	0.79 (0.03)	0.79 (0.03)	0.03 (0.04)	0.00 (0.01)	0.00 (0.01)	0.77 (0.06)	0.79 (0.03)	0.79 (0.03)	0.04 (0.06)	0.00 (0.02)	0.00 (0.02)

Note. Average mean absolute error (MAE) and coefficient of determination (R^2) displayed with standard deviation (SD) appearing in parentheses. Pure = no feature selection; FS+HPO = feature selection and hyperparameter optimization; Ridge(adj. λ): default values manually adjusted; nr = no confound regression; nr-cr = age, sex & education regressed from target only; cr-cr = age, sex & education regressed from target and features.

Supplementary Table S24. Regression performance across feature sets and pipeline configurations for global cognition in comparison to dummy regressor.

		421			1621		
		nr	nr-cr	cr-cr	nr	nr-cr	cr-cr
Pure	SVR	86%	46%	54%	92%	48%	50%
	RVR	60%	12%	14%	82%	26%	46%
	Elastic Net	22%	0%	0%	22%	0%	0%
	Lasso	22%	0%	0%	22%	0%	0%
	Ridge ($\lambda = 1$)	0%	0%	0%	0%	0%	0%
	Ridge (adj. λ)	48%	14%	10%	6%	2%	2%
FS &HPO	SVR	60%	50%	46%	72%	50%	40%
	RVR	42%	8%	4%	30%	14%	14%
	Elastic Net	90%	56%	46%	90%	64%	60%
	Lasso	98%	40%	42%	92%	76%	74%
	Ridge	76%	60%	42%	80%	58%	58%

Note. Percentage (%) of folds for which real models outperform dummy regressor in terms of coefficient of determination (R^2). Pure = no feature selection; FS+HPO = feature selection and hyperparameter optimization; Ridge(adj. λ): default values manually adjusted; nr = no confound regression; nr-cr = age, sex & education regressed from target only; cr-cr = age, sex & education regressed from target and features. Colour scheme: green = real model outperforms dummy in ≥ 80% of folds; orange = real model outperforms dummy in 50 - 80% of folds; red = real model outperforms dummy in ≤ 50% of folds.

Validation Analyses
Parcellation granularity

Supplementary Table S25. 800-Node Parcellation: Classification performance across feature sets in the matched sample for global cognition.

		Feature Set 21		Feature Set 821		Feature Set 2400		Feature Set 3221	
		nr	cr	nr	cr	nr	cr	nr	cr
Pure	SVM (RBF)	49.05% (5.99%)	50.28% (6.04%)	55.52% (6.03%)	56.53% (5.90%)	56.01% (6.15%)	56.01% (6.20%)	55.78% (6.21%)	52.24% (4.15%)
	SVM (linear)	52.19% (6.98%)	51.35% (6.20%)	53.85% (7.38%)	53.93% (6.90%)	53.87% (7.55%)	54.55% (6.24%)	54.83% (6.82%)	52.24% (4.15%)
	SVM (poly)	48.40% (6.48%)	49.06% (5.92%)	51.24% (5.10%)	50.48% (4.92%)	52.24% (4.15%)	51.93% (4.06%)	51.62% (4.63%)	52.24% (4.15%)
	KNN	47.79% (6.18%)	49.88% (5.94%)	52.16% (5.68%)	52.07% (6.59%)	53.83% (6.33%)	52.41% (5.74%)	53.01% (6.59%)	52.24% (4.15%)
	DT	47.87% (7.33%)	48.90% (6.55%)	50.39% (7.10%)	51.05% (5.73%)	53.17% (6.56%)	51.44% (6.94%)	51.71% (5.55%)	52.24% (4.15%)
	NB	51.45% (5.92%)	51.75% (5.47%)	53.16% (5.92%)	51.98% (6.18%)	52.35% (5.55%)	51.82% (5.47%)	52.39% (5.75%)	52.24% (4.15%)
	LDA	52.60% (6.73%)	52.16% (6.18%)	54.12% (6.36%)	50.99% (6.68%)	55.84% (5.69%)	53.47% (7.25%)	55.95% (5.44%)	52.24% (4.15%)
FS	SVM (RBF)	49.05% (6.82%)	50.00% (6.98%)	56.30% (5.88%)	55.87% (7.11%)	53.55% (6.22%)	53.51% (7.14%)	54.63% (7.54%)	53.86% (8.06%)
	SVM (linear)	51.21% (6.57%)	49.94% (6.25%)	54.00% (6.03%)	52.46% (6.79%)	52.05% (6.95%)	51.37% (8.05%)	52.43% (6.33%)	52.02% (7.22%)
	SVM (poly)	51.97% (5.11%)	51.49% (4.35%)	53.46% (6.47%)	53.43% (5.31%)	53.73% (5.72%)	53.39% (5.93%)	54.05% (5.00%)	54.41% (5.91%)
	KNN	50.23% (5.78%)	53.82% (7.21%)	51.23% (5.33%)	52.92% (6.80%)	51.86% (5.13%)	53.47% (5.14%)	51.74% (5.02%)	53.58% (5.58%)
	DT	49.76% (6.59%)	50.77% (6.56%)	51.05% (7.28%)	50.49% (6.63%)	52.17% (6.78%)	51.42% (6.67%)	50.94% (6.84%)	53.08% (6.49%)
	NB	51.16% (6.13%)	50.96% (6.48%)	52.55% (5.02%)	52.24% (5.84%)	50.99% (5.16%)	50.73% (5.48%)	52.00% (5.37%)	51.27% (5.44%)
	LDA	50.47% (6.00%)	50.74% (6.31%)	53.51% (5.89%)	52.04% (6.91%)	52.47% (7.72%)	52.21% (7.26%)	54.14% (7.34%)	52.48% (6.86%)

Note. Mean balanced accuracies (BAC (%)) displayed with standard deviation (SD) appearing in parentheses. Pure = pipeline without feature selection; FS = univariate FS (ANOVA F-test) pipeline; nr = no confound regression; cr = age, sex & education regressed from features.

Supplementary Table S26. 800-Node Parcellation: Classification performance across feature sets in the matched sample for global cognition in comparison to dummy classifier.

		Feature Set 21		Feature Set 821		Feature Set 2400		Feature Set 3221	
		nr	cr	nr	cr	nr	cr	nr	cr
Pure	SVM (RBF)	26%	46%	78%	84%	76%	74%	76%	74%
	SVM (linear)	62%	54%	66%	68%	72%	76%	72%	70%
	SVM (poly)	38%	42%	52%	44%	66%	68%	58%	54%
	KNN	28%	46%	56%	58%	64%	58%	66%	52%
	DT	44%	48%	50%	52%	70%	60%	60%	52%
	NB	52%	56%	62%	56%	64%	54%	58%	64%
	LDA	62%	58%	76%	54%	78%	64%	80%	74%
FS	SVM (RBF)	40%	48%	92%	80%	68%	66%	66%	62%
	SVM (linear)	54%	48%	70%	56%	58%	50%	56%	56%
	SVM (poly)	54%	60%	64%	62%	70%	68%	72%	72%
	KNN	48%	68%	56%	58%	56%	66%	64%	72%
	DT	48%	48%	54%	50%	60%	56%	48%	56%
	NB	52%	44%	68%	62%	62%	54%	64%	56%
	LDA	50%	52%	76%	60%	50%	54%	76%	58%

Note. Percentage (%) of folds for which real models outperform dummy classifier in terms of balanced accuracy (BAC). nr = no confound regression; cr = age, sex & education regressed from features. Colour scheme: green = real model outperforms dummy in ≥ 80% of folds; orange = real model outperforms dummy in 50 - 80% of folds; red = real model outperforms dummy in ≤ 50% of folds.

Supplementary Table S27. 800-Node Parcellation: Prediction performance for global cognition across feature sets in the unmatched sample.

		821									3221								
		MAE			R^2			folds > ref.			MAE			R^2			folds > ref.		
		nr	nr-cr	cr-cr	nr	nr-cr	cr-cr	nr	nr-cr	cr-cr	nr	nr-cr	cr-cr	nr	nr-cr	cr-cr	nr	nr-cr	cr-cr
Pure	SVR	0.75	0.79	0.79	0.07	0.00	0.01	90%	52%	56%	0.75	0.79	0.79	0.07	0.01	0.02	90%	62%	70%
		(0.06)	(0.03)	(0.03)	(0.06)	(0.05)	(0.04)				(0.06)	(0.03)	(0.03)	(0.06)	(0.04)	(0.04)			
	RVR	0.76	0.81	0.80	0.05	-0.03	-0.02	76%	30%	38%	0.75	0.80	0.79	0.07	0.00	0.01	84%	46%	48%
		(0.06)	(0.04)	(0.03)	(0.08)	(0.07)	(0.07)				(0.06)	(0.04)	(0.04)	(0.08)	(0.06)	(0.06)			
	Elastic	0.80	0.79	0.79	-0.02	0.00	0.00	44%	0%	0%	0.80	0.79	0.79	-0.02	0.00	0.00	44%	0%	0%
	Net	(0.07)	(0.03)	(0.03)	(0.03)	(0.00)	(0.00)				(0.07)	(0.03)	(0.03)	(0.03)	(0.00)	(0.00)			
	Lasso	0.80	0.79	0.79	-0.02	0.00	0.00	44%	0%	0%	0.80	0.79	0.79	-0.02	0.00	0.00	44%	0%	0%
		(0.07)	(0.03)	(0.03)	(0.03)	(0.00)	(0.00)				(0.07)	(0.03)	(0.03)	(0.03)	(0.00)	(0.00)			
	Ridge	1.97	2.07	2.05	-5.57	-5.99	-5.82	0%	0%	0%	0.95	1.02	1.02	-0.50	-0.68	-0.66	6%	0%	0%
	($\lambda = 1$)	(0.12)	(0.14)	(0.12)	(1.44)	(0.78)	(0.79)				(0.07)	(0.08)	(0.07)	(0.29)	(0.24)	(0.23)			
	Ridge	0.80	0.85	0.85	-0.06	-0.14	-0.14	28%	10%	6%	0.84	0.90	0.90	-0.18	-0.29	-0.28	18%	2%	2%
	(adj. λ)	(0.06)	(0.05)	(0.05)	(0.14)	(0.11)	(0.11)				(0.06)	(0.06)	(0.06)	(0.2)	(0.17)	(0.17)			
FS &	SVR	0.76	0.79	0.79	0.04	0.00	0.00	74%	62%	56%	0.76	0.79	0.79	0.04	-0.01	0.00	72%	46%	50%
HPO		(0.06)	(0.03)	(0.03)	(0.06)	(0.03)	(0.04)				(0.06)	(0.03)	(0.03)	(0.06)	(0.02)	(0.02)			
	RVR	0.81	0.85	0.85	-0.04	-0.14	-0.14	32%	0%	4%	0.81	0.86	0.85	-0.05	-0.15	-0.13	40%	4%	6%
		(0.07)	(0.05)	(0.04)	(0.10)	(0.09)	(0.09)				(0.07)	(0.05)	(0.05)	(0.12)	(0.10)	(0.09)			
	Elastic	0.76	0.79	0.79	0.06	0.01	0.01	82%	66%	66%	0.76	0.79	0.79	0.07	0.01	0.01	86%	64%	62%
	Net	(0.06)	(0.03)	(0.03)	(0.07)	(0.03)	(0.03)				(0.06)	(0.03)	(0.03)	(0.07)	(0.03)	(0.03)			
	Lasso	0.77	0.79	0.79	0.04	0.01	0.01	88%	82%	84%	0.77	0.79	0.79	0.04	0.01	0.01	84%	70%	74%
		(0.06)	(0.03)	(0.03)	(0.04)	(0.01)	(0.01)				(0.06)	(0.03)	(0.03)	(0.04)	(0.02)	(0.02)			
	Ridge	0.77	0.79	0.79	0.04	0.01	0.01	72%	66%	68%	0.76	0.79	0.79	0.05	0.00	0.00	80%	60%	60%
		(0.06)	(0.03)	(0.03)	(0.06)	(0.03)	(0.03)				(0.06)	(0.03)	(0.03)	(0.07)	(0.02)	(0.02)			

Note. Average mean absolute error (MAE) and coefficient of determination (R^2) displayed with standard deviation (SD) appearing in parentheses. Folds > ref. = percentage (%) of folds for which real models outperform dummy regressor in terms of coefficient of determination (R^2); Pure = pipeline without feature selection; FS = pipeline with feature selection; Ridge(adj. λ): default values manually adjusted; nr = no confound regression; nr-cr = age, sex & education regressed from target; cr-cr = age, sex & education regressed from target and features. Colour scheme: green = real model outperforms dummy in ≥ 80% of folds; orange = real model outperforms dummy in 50 - 80% of folds; red = real model outperforms dummy in ≤ 50% of folds.

Supplementary Figure S28. Classification and prediction performance for global cognition in the 800-nodes parcellation. (A) Mean balanced accuracies (BACs (%)) across folds are displayed for matched sample. Error bars correspond to standard deviation (SD). (B) Mean Absolute Error (MAE) across folds is displayed for unmatched sample. *Ridge default values manually adjusted.

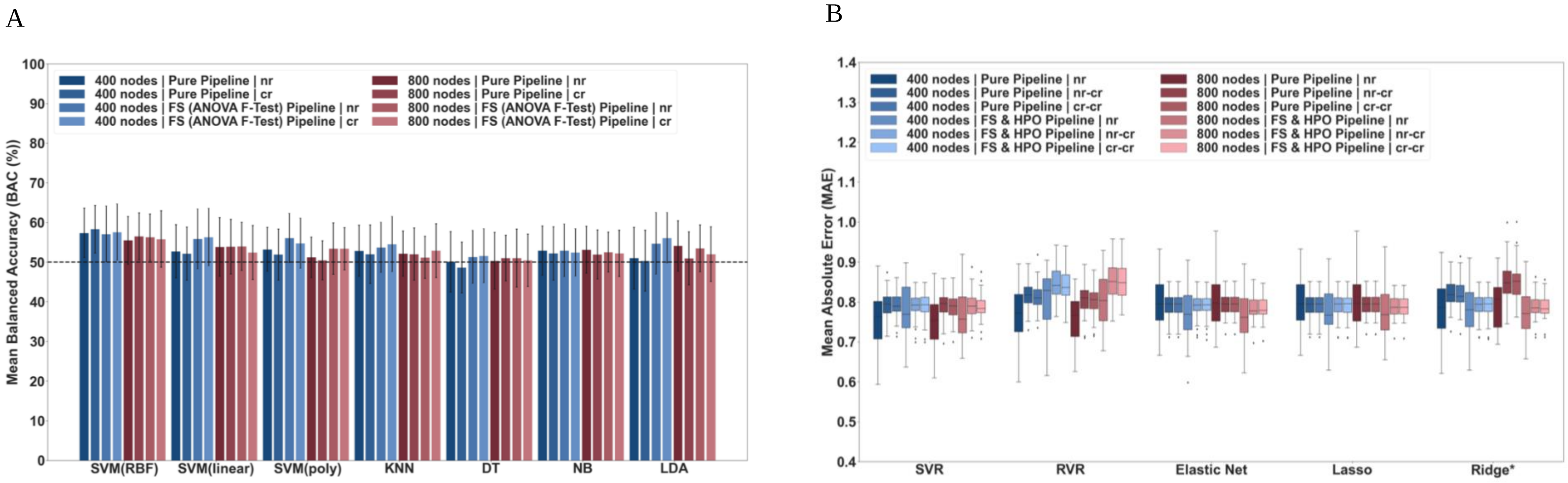

Classification performance in male & female subsamples

Supplementary Table S29. Demographic information on male and female subsamples.

	Males						Females					
	Initial Sample			Matched Sample			Initial Sample			Matched Sample		
	N	M_{age}	Education	N	M_{age}	Education	N	M_{age}	Education	N	M_{age}	Education
High	-	-	-	143	67.21 (6.5)	7.08 (1.90)	-	-	-	116	65.27 (5.65)	5.92 (1.74)
Low	-	-	-	143	68.41 (6.35)	6.83 (1.84)	-	-	-	116	65.39 (5.34)	5.84 (1.66)
Total	441	67.50 (6.8)	6.95 (1.91)	286	67.81 (6.44)	6.96 (1.87)	372	66.38 (6.53)	5.93 (1.84)	232	65.33 (5.48)	5.88 (1.70)

Note. Standard deviation (SD) appears in parentheses.

Supplementary Table S30. Classification performance in male and female subsamples of the matched sample for feature set 421.

	Pure				FS			
	Females	folds > ref.	Males	folds > ref.	Females	folds > ref.	Males	folds > ref.
SVM (RBF)	54.23% (9.20%)	64%	53.82% (8.67%)	62%	54.05% (9.65%)	64%	54.65% (8.83%)	72%
SVM (linear)	50.18% (9.84%)	56%	53.46% (9.75%)	60%	54.80% (9.49%)	76%	52.85% (8.91%)	58%
SVM (poly)	53.49% (7.54%)	64%	51.56% (7.25%)	50%	53.33% (7.85%)	62%	53.00% (8.94%)	58%
KNN	50.92% (8.42%)	56%	50.88% (6.67%)	52%	50.66% (8.92%)	54%	52.97% (9.68%)	58%
DT	49.48% (10.79%)	44%	53.97% (11.07%)	58%	50.41% (9.41%)	52%	51.43% (10.15%)	56%
NB	52.53% (9.95%)	58%	51.93% (9.42%)	54%	51.01% (8.76%)	58%	51.04% (9.87%)	50%
LDA	49.69% (9.64%)	46%	51.30% (9.85%)	52%	55.57% (7.97%)	70%	53.01% (9.07%)	54%

Note. Mean balanced accuracies (BAC (%)) displayed with standard deviation (SD) appearing in parentheses. Folds > ref. = percentage (%) of folds for which real models outperform dummy classifier in terms of balanced accuracy (BAC); Pure = pipeline without feature selection; FS = univariate FS (ANOVA F-test) pipeline; nr = no confound regression; cr = age, sex & education regressed from features. Colour scheme: green = real model outperforms dummy in ≥ 80% of folds; orange = real model outperforms dummy in 50 - 80% of folds; red = real model outperforms dummy in ≤ 50% of folds.

Supplementary Table S31. Prediction performance of global cognition in male and female subsamples across pipeline configurations in feature set 421.

		**Females**									**Males**								
		MAE			R^2			folds > ref.			MAE			R^2			folds > ref.		
		nr	nr-cr	cr-cr	nr	nr-cr	cr-cr	nr	nr-cr	cr-cr	nr	nr-cr	cr-cr	nr	nr-cr	cr-cr	nr	nr-cr	cr-cr
Pure	SVR	0.73 (0.10)	0.81 (0.05)	0.80 (0.05)	0.04 (0.12)	0.01 (0.09)	0.02 (0.09)	86%	58%	64%	0.78 (0.09)	0.78 (0.04)	0.78 (0.04)	0.04 (0.11)	-0.02 (0.08)	-0.03 (0.08)	78%	40%	38%
	RVR	0.74 (0.09)	0.81 (0.05)	0.81 (0.05)	0.03 (0.19)	0.00 (0.12)	0.01 (0.12)	76%	50%	62%	0.79 (0.10)	0.82 (0.06)	0.82 (0.06)	-0.01 (0.14)	-0.10 (0.12)	-0.10 (0.13)	58%	24%	28%
	Elastic Net	0.82 (0.09)	0.82 (0.03)	0.82 (0.03)	-0.03 (0.07)	0.00 (0.00)	0.00 (0.00)	0%	2%	2%	0.82 (0.09)	0.78 (0.03)	0.78 (0.03)	-0.03 (0.07)	0.00 (0.00)	0.00 (0.00)	0%	4%	4%
	Lasso	0.76 (0.10)	0.82 (0.03)	0.82 (0.03)	-0.03 (0.05)	0.00 (0.00)	0.00 (0.00)	0%	2%	2%	0.82 (0.09)	0.78 (0.03)	0.78 (0.03)	-0.03 (0.07)	0.00 (0.00)	0.00 (0.00)	48%	4%	4%
	Ridge (λ=1)	1.52 (0.18)	1.64 (0.16)	1.63 (0.15)	-3.35 (1.46)	-3.12 (0.69)	-3.09 (0.70)	0%	0%	0%	2.01 (0.22)	2.19 (0.23)	2.20 (0.22)	-5.70 (2.17)	-6.56 (1.49)	-6.71 (1.49)	0%	0%	0%
	Ridge (adj. λ)	0.74 (0.09)	0.01 (0.21)	0.80 (0.06)	0.01 (0.21)	0.02 (0.13)	0.02 (0.12)	70%	60%	62%	0.83 (0.09)	0.86 (0.07)	0.86 (0.07)	-0.12 (0.18)	-0.20 (0.16)	-0.20 (0.16)	32%	8%	10%
FS & HPO	SVR	0.76 (0.09)	0.81 (0.03)	0.82 (0.04)	-0.04 (0.13)	0.00 (0.04)	-0.01 (0.05)	54%	56%	40%	0.81 (0.09)	0.78 (0.03)	0.78 (0.03)	-0.02 (0.09)	-0.01 (0.01)	-0.01 (0.01)	54%	6%	8%
	RVR	0.78 (0.10)	0.84 (0.07)	0.83 (0.03)	-0.12 (0.27)	-0.12 (0.15)	-0.04 (0.05)	34%	18%	16%	0.85 (0.09)	0.86 (0.07)	0.87 (0.07)	-0.12 (0.13)	-0.22 (0.16)	-0.23 (0.17)	22%	2%	8%
	Elastic Net	0.74 (0.09)	0.81 (0.05)	0.81 (0.04)	0.03 (0.14)	0.01 (0.09)	0.02 (0.08)	82%	64%	68%	0.80 (0.09)	0.78 (0.03)	0.78 (0.03)	0.01 (0.09)	-0.01 (0.02)	-0.01 (0.02)	76%	40%	44%
	Lasso	0.75 (0.10)	0.82 (0.03)	0.82 (0.03)	0.01 (0.11)	-0.01 (0.03)	-0.01 (0.03)	86%	26%	32%	0.80 (0.09)	0.78 (0.03)	0.78 (0.03)	0.01 (0.08)	0.00 (0.01)	0.00 (0.01)	86%	4%	4%
	Ridge	0.76 (0.09)	0.81 (0.03)	0.81 (0.04)	-0.01 (0.16)	0.01 (0.05)	0.01 (0.05)	74%	64%	70%	0.80 (0.09)	0.78 (0.03)	0.78 (0.03)	0.01 (0.10)	0.00 (0.01)	0.00 (0.01)	72%	46%	52%

Note. Average mean absolute error (MAE) and coefficient of determination (R^2) displayed with standard deviation (SD) appearing in parentheses. Folds > ref. = percentage (%) of folds for which real models outperform dummy regressor in terms of coefficient of determination (R^2); Pure = pipeline without feature selection; FS = pipeline with feature selection; nr = no confound regression; Ridge(adj. λ): default values manually adjusted; nr-cr = age, sex & education regressed from target; cr-cr = age, sex & education regressed from target and features. Colour scheme: green = real model outperforms dummy in ≥ 80% of folds; orange = real model outperforms dummy in 50 - 80% of folds; red = real model outperforms dummy in ≤ 50% of folds.

Supplementary Figure S32. Classification and prediction performance results of global cognition in male and female subsamples. (A) Mean balanced accuracies (BACs (%)) across folds are displayed for matched sample. Error bars correspond to standard deviation (SD). (B) Mean Absolute Error (MAE) across folds is displayed for unmatched sample. *Ridge default values manually adjusted.

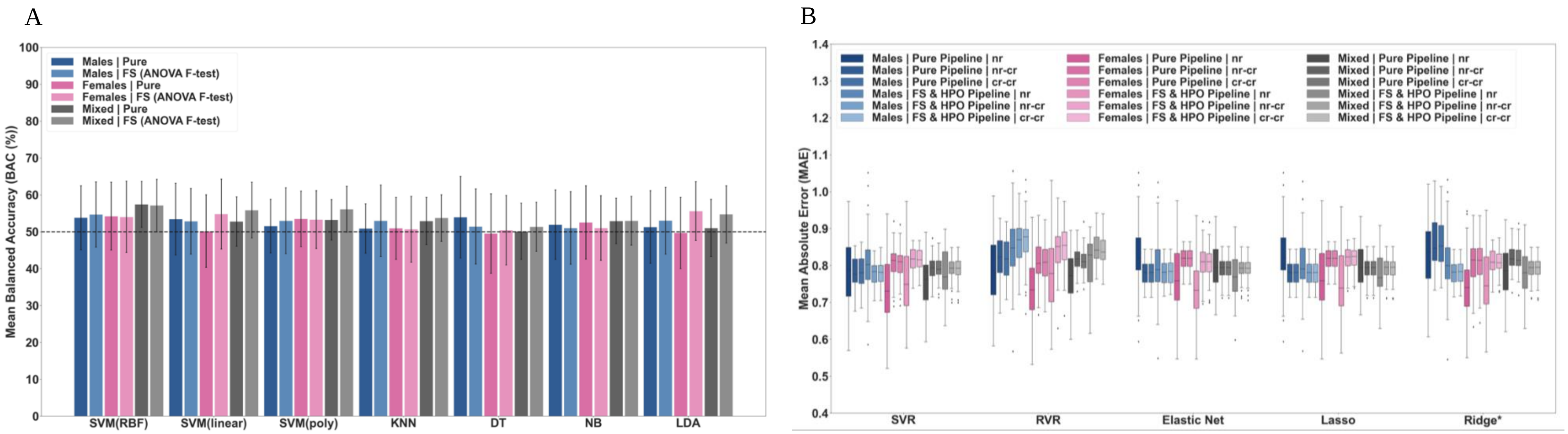

Absolute correlation values

Supplementary Table S33. Classification performance across feature sets based on RSFC strength measures from absolute positive and negative correlation values in the matched sample for global cognition.

		421				1621			
		nr	folds > ref.	cr	folds > ref.	nr	folds > ref.	cr	folds > ref.
Pure	SVM (RBF)	54.84% (6.30%)	72%	55.72% (6.79%)	78%	55.29% (5.59%)	80%	55.57% (5.88%)	80%
	SVM (linear)	51.96% (5.60%)	64%	51.92% (5.62%)	58%	54.04% (6.64%)	66%	53.28% (7.04%)	70%
	SVM (poly)	54.84% (6.75%)	72%	53.29% (5.95%)	66%	54.03% (5.55%)	72%	52.72% (5.81%)	56%
	KNN	53.30% (8.33%)	56%	53.33% (7.44%)	52%	53.90% (5.62%)	74%	54.25% (7.69%)	70%
	DT	50.82% (7.51%)	50%	52.25% (6.93%)	60%	50.43% (6.60%)	46%	49.71% (6.20%)	50%
	NB	51.83% (5.41%)	54%	50.83% (5.40%)	50%	52.14% (5.48%)	58%	51.68% (5.76%)	58%
	LDA	48.19% (6.65%)	34%	47.91% (6.97%)	34%	53.08% (6.81%)	68%	51.34% (8.52%)	48%
FS	SVM (RBF)	54.85% (5.80%)	74%	55.34% (5.11%)	78%	55.38% (6.66%)	74%	55.95% (6.56%)	82%
	SVM (linear)	54.84% (5.99%)	74%	55.22% (6.38%)	72%	56.25% (7.21%)	74%	55.21% (7.12%)	76%
	SVM (poly)	53.00% (7.28%)	64%	54.43% (6.22%)	68%	55.11% (6.31%)	78%	55.65% (6.61%)	82%
	KNN	52.15% (4.94%)	60%	51.95% (5.99%)	62%	51.85% (5.96%)	52%	54.41% (6.37%)	76%
	DT	50.51% (6.94%)	56%	51.44% (6.41%)	56%	51.90% (6.05%)	60%	50.99% (7.36%)	46%
	NB	51.91% (5.63%)	56%	51.79% (5.58%)	50%	52.40% (5.95%)	58%	51.67% (5.98%)	58%
	LDA	54.72% (5.97%)	78%	55.60% (6.58%)	80%	56.79% (6.84%)	78%	54.74% (7.17%)	72%

Note. Mean balanced accuracies (BAC (%)) displayed with standard deviation (SD) appearing in parentheses. Folds > ref. = percentage (%) of folds for which real models outperform dummy classifier in terms of balanced accuracy (BAC); Pure = pipeline without feature selection; FS = univariate FS (ANOVA F-test) pipeline; nr = no confound regression; cr = age, sex & education regressed from features. Colour scheme: green = real model outperforms dummy in ≥ 80% of folds; orange = real model outperforms dummy in 50 - 80% of folds; red = real model outperforms dummy in ≤ 50% of folds.

Supplementary Table S34. Prediction performance of global cognition across feature sets based on RSFC strength measures from absolute positive and negative correlation values across pipeline configurations.

		421									1621								
		MAE			R^2			folds > ref.			MAE			R^2			folds > ref.		
		nr	nr-cr	cr-cr	nr	nr-cr	cr-cr	nr	nr-cr	cr-cr	nr	nr-cr	cr-cr	nr	nr-cr	cr-cr	nr	nr-cr	cr-cr
Pure	SVR	0.76 (0.06)	0.79 (0.03)	0.79 (0.03)	0.05 (0.06)	-0.01 (0.04)	-0.01 (0.05)	84%	42%	50%	0.75 (0.06)	0.79 (0.03)	0.79 (0.03)	0.08 (0.05)	0.00 (0.04)	0.01 (0.04)	94%	42%	66%
	RVR	0.79 (0.06)	0.83 (0.04)	0.82 (0.04)	-0.02 (0.07)	-0.08 (0.07)	-0.07 (0.07)	44%	20%	20%	0.79 (0.06)	0.80 (0.03)	0.80 (0.04)	-0.02 (0.07)	-0.02 (0.06)	-0.01 (0.06)	80%	40%	46%
	Elastic Net	0.80 (0.06)	0.79 (0.03)	0.79 (0.03)	-0.01 (0.02)	0.00 (0.00)	0.00 (0.00)	50%	4%	4%	0.80 (0.06)	0.79 (0.03)	0.79 (0.03)	-0.01 (0.02)	0.00 (0.00)	0.00 (0.00)	50%	4%	4%
	Lasso	0.80 (0.06)	0.79 (0.03)	0.79 (0.03)	-0.01 (0.02)	0.00 (0.00)	0.00 (0.00)	50%	4%	4%	0.80 (0.06)	0.79 (0.03)	0.79 (0.03)	-0.01 (0.02)	0.00 (0.00)	0.00 (0.00)	50%	4%	4%
	Ridge (λ=1)	1.09 (0.07)	1.16 (0.09)	1.16 (0.10)	-1.01 (0.34)	-1.21 (0.40)	-1.22 (0.42)	0%	0%	0%	1.52 (0.10)	1.78 (0.11)	1.71 (0.10)	-2.82 (0.60)	-4.07 (0.54)	-3.70 (0.51)	0%	0%	0%
	Ridge (adj. λ)	0.79 (0.06)	0.83 (0.04)	0.82 (0.04)	-0.02 (0.09)	-0.10 (0.08)	-0.10 (0.09)	54%	8%	10%	0.83 (0.06)	0.92 (0.06)	0.91 (0.06)	-0.15 (0.12)	-0.35 (0.13)	-0.34 (0.13)	14%	0%	0%
FS & HPO	SVR	0.78 (0.07)	0.79 (0.03)	0.79 (0.03)	0.00 (0.04)	0.00 (0.01)	0.00 (0.01)	62%	52%	54%	0.77 (0.07)	0.79 (0.03)	0.79 (0.03)	0.04 (0.05)	0.00 (0.02)	0.00 (0.02)	86%	60%	58%
	RVR	0.81 (0.07)	0.84 (0.04)	0.84 (0.04)	-0.05 (0.08)	-0.13 (0.09)	-0.13 (0.09)	28%	6%	4%	0.80 (0.07)	0.84 (0.05)	0.85 (0.05)	-0.02 (0.10)	-0.14 (0.09)	-0.15 (0.09)	48%	8%	6%
	Elastic Net	0.76 (0.06)	0.79 (0.03)	0.79 (0.03)	0.07 (0.04)	0.00 (0.02)	0.00 (0.02)	100%	56%	58%	0.76 (0.06)	0.79 (0.03)	0.79 (0.03)	0.07 (0.05)	0.01 (0.02)	0.01 (0.02)	92%	72%	78%
	Lasso	0.78 (0.06)	0.79 (0.03)	0.79 (0.03)	0.03 (0.02)	0.00 (0.00)	0.00 (0.00)	94%	32%	40%	0.77 (0.06)	0.79 (0.03)	0.79 (0.03)	0.04 (0.03)	0.00 (0.00)	0.00 (0.00)	92%	6%	6%
	Ridge	0.78 (0.07)	0.79 (0.03)	0.79 (0.03)	0.02 (0.04)	0.00 (0.01)	0.00 (0.01)	80%	52%	52%	0.76 (0.06)	0.79 (0.03)	0.79 (0.03)	0.06 (0.05)	0.00 (0.01)	0.00 (0.01)	92%	54%	56%

Note. Average mean absolute error (MAE) and coefficient of determination (R^2) displayed with standard deviation (SD) appearing in parentheses. Folds > ref. = percentage (%) of folds for which real models outperform dummy regressor in terms of coefficient of determination (R^2); Pure = pipeline without feature selection; FS = pipeline with feature selection; nr = no confound regression; Ridge(adj. λ): default values manually adjusted; nr-cr = age, sex & education regressed from target; cr-cr = age, sex & education regressed from target and features. Colour scheme: green = real model outperforms dummy in ≥ 80% of folds; orange = real model outperforms dummy in 50 - 80% of folds; red = real model outperforms dummy in ≤ 50% of folds.

Supplementary Figure S35. Classification and prediction performance results of global cognition using features (RSFC strength measures) based on positive (main analysis) and absolute positive and negative correlation values in feature set 421. (A) Mean balanced accuracies (BACs (%)) across folds are displayed for matched sample. Error bars correspond to standard deviation (SD). (B) Mean Absolute Error (MAE) across folds is displayed for unmatched sample. *Ridge default values manually adjusted.

A B

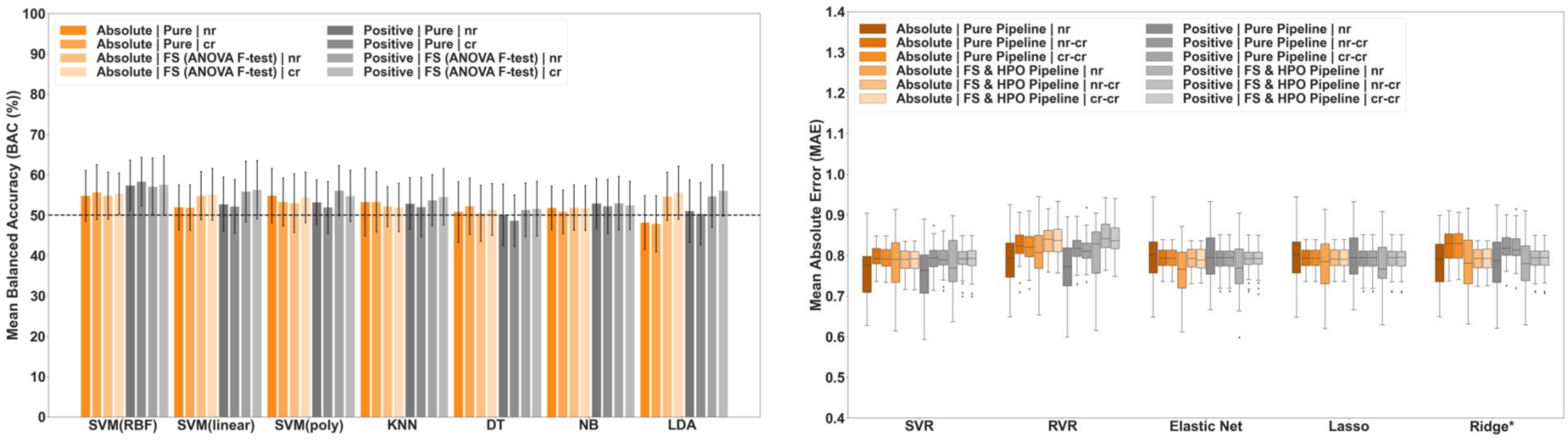

Negative correlation values

Supplementary Table S36. Classification performance across feature sets based on RSFC strength measures from negative correlation values in the matched sample for global cognition.

		421				1621			
		nr	folds > ref.	cr	folds > ref.	nr	folds > ref.	cr	folds > ref.
Pure	SVM (RBF)	53.63% (5.01%)	72%	53.21% (6.20%)	62%	52.75% (5.50%)	64%	51.93% (5.47%)	62%
	SVM (linear)	50.26% (6.88%)	46%	49.53% (6.74%)	34%	50.82% (6.85%)	50%	50.86% (6.70%)	50%
	SVM (poly)	51.20% (4.36%)	52%	50.59% (4.63%)	48%	51.89% (5.49%)	52%	51.04% (4.62%)	50%
	KNN	50.46% (6.33%)	52%	50.12% (6.72%)	48%	51.54% (5.79%)	48%	49.80% (5.77%)	40%
	DT	50.62% (7.84%)	48%	49.74% (6.61%)	46%	51.99% (8.47%)	54%	52.06% (7.48%)	60%
	NB	52.14% (5.58%)	62%	52.45% (5.97%)	62%	52.11% (5.33%)	62%	52.69% (5.71%)	64%
	LDA	50.53% (6.99%)	52%	50.45% (7.28%)	50%	50.66% (5.20%)	48%	50.82% (6.93%)	54%
FS	SVM (RBF)	51.90% (5.41%)	58%	51.62% (5.06%)	58%	51.65% (5.35%)	54%	52.58% (6.84%)	66%
	SVM (linear)	54.26% (5.49%)	70%	53.51% (5.93%)	62%	53.20% (6.82%)	66%	52.32% (7.38%)	58%
	SVM (poly)	52.11% (3.79%)	66%	51.42% (4.16%)	54%	51.89% (5.28%)	56%	51.98% (6.08%)	56%
	KNN	51.88% (7.12%)	60%	50.12% (5.49%)	42%	53.28% (5.68%)	58%	50.54% (5.44%)	52%
	DT	51.65% (7.06%)	54%	48.42% (6.31%)	28%	49.11% (6.80%)	42%	49.08% (6.96%)	48%
	NB	52.21% (5.68%)	64%	52.42% (6.51%)	62%	51.12% (5.30%)	52%	52.28% (5.99%)	64%
	LDA	54.73% (5.18%)	80%	54.05% (5.62%)	68%	51.91% (6.63%)	52%	52.89% (6.71%)	60%

Note. Mean balanced accuracies (BAC (%)) displayed with standard deviation (SD) appearing in parentheses. Folds > ref. = percentage (%) of folds for which real models outperform dummy classifier in terms of balanced accuracy (BAC); Pure = pipeline without feature selection; FS = univariate FS (ANOVA F-test) pipeline; nr = no confound regression; cr = age, sex & education regressed from features. Colour scheme: green = real model outperforms dummy in ≥ 80% of folds; orange = real model outperforms dummy in 50 - 80% of folds; red = real model outperforms dummy in ≤ 50% of folds.

Supplementary Table S37. Prediction performance of global cognition across feature sets based on RSFC strength measures from negative correlation across pipeline configurations.

		421									1621								
		MAE			R^2			folds > ref.			MAE			R^2			folds > ref.		
		nr	nr-cr	cr-cr	nr	nr-cr	cr-cr	nr	nr-cr	cr-cr	nr	nr-cr	cr-cr	nr	nr-cr	cr-cr	nr	nr-cr	cr-cr
Pure	SVR	0.77	0.81	0.81	0.03	-0.04	-0.03	76%	12%	16%	0.77	0.80	0.80	0.05	-0.02	-0.02	84%	32%	30%
		(0.06)	(0.03)	(0.03)	(0.06)	(0.03)	(0.04)				(0.06)	(0.03)	(0.03)	(0.06)	(0.04)	(0.04)			
	RVR	0.79	0.83	0.83	-0.02	-0.09	-0.08	50%	14%	18%	0.78	0.82	0.82	0.02	-0.06	-0.05	64%	18%	28%
		(0.06)	(0.04)	(0.04)	(0.09)	(0.07)	(0.08)				(0.06)	(0.03)	(0.03)	(0.08)	(0.06)	(0.07)			
	Elastic Net	0.80	0.79	0.79	-0.01	0.00	0.00	52%	0%	0%	0.80	0.79	0.79	-0.01	0.00	0.00	52%	0%	0%
		(0.07)	(0.02)	(0.02)	(0.02)	(0.00)	(0.00)				(0.07)	(0.02)	(0.02)	(0.02)	(0.00)	(0.00)			
	Lasso	0.80	0.79	0.79	-0.01	0.00	0.00	52%	0%	0%	0.80	0.79	0.79	-0.01	0.00	0.00	52%	0%	0%
		(0.07)	(0.02)	(0.02)	(0.02)	(0.00)	(0.00)				(0.07)	(0.02)	(0.02)	(0.02)	(0.00)	(0.00)			
	Ridge (λ=1)	1.14	1.19	1.21	-1.25	-1.39	-1.41	0%	0%	0%	1.26	1.30	1.31	-1.57	-1.71	-1.72	0%	0%	0%
		(0.10)	(0.08)	(0.09)	(0.39)	(0.33)	(0.33)				(0.10)	(0.08)	(0.09)	(0.37)	(0.36)	(0.37)			
	Ridge (adj. λ)	0.81	0.85	0.85	-0.07	-0.15	-0.15	26%	4%	2%	0.88	0.92	0.92	-0.26	-0.34	-0.35	4%	0%	0%
		(0.07)	(0.04)	(0.04)	(0.07)	(0.07)	(0.08)				(0.08)	(0.05)	(0.06)	(0.13)	(0.14)	(0.15)			
FS & HPO	SVR	0.78	0.79	0.79	0.00	-0.01	-0.01	64%	14%	10%	0.77	0.79	0.79	0.02	0.00	0.00	82%	56%	58%
		(0.07)	(0.02)	(0.02)	(0.06)	(0.01)	(0.01)				(0.07)	(0.03)	(0.03)	(0.06)	(0.02)	(0.03)			
	RVR	0.81	0.86	0.85	-0.07	-0.16	-0.15	28%	2%	2%	0.81	0.85	0.85	-0.06	-0.13	-0.13	36%	8%	8%
		(0.07)	(0.04)	(0.04)	(0.09)	(0.08)	(0.08)				(0.07)	(0.04)	(0.04)	(0.09)	(0.09)	(0.10)			
	Elastic Net	0.77	0.79	0.79	0.04	0.00	0.00	84%	46%	50%	0.77	0.79	0.79	0.04	0.01	0.01	82%	72%	68%
		(0.07)	(0.02)	(0.02)	(0.04)	(0.01)	(0.01)				(0.07)	(0.02)	(0.02)	(0.05)	(0.02)	(0.02)			
	Lasso	0.79	0.79	0.79	0.01	0.00	0.00	82%	26%	26%	0.78	0.79	0.79	0.02	0.01	0.01	84%	74%	72%
		(0.06)	(0.02)	(0.02)	(0.03)	(0.00)	(0.00)				(0.07)	(0.02)	(0.02)	(0.03)	(0.01)	(0.01)			
	Ridge	0.78	0.79	0.79	0.02	0.00	0.00	74%	62%	58%	0.78	0.79	0.79	0.03	0.00	0.00	84%	66%	68%
		(0.07)	(0.02)	(0.02)	(0.04)	(0.01)	(0.01)				(0.07)	(0.02)	(0.03)	(0.05)	(0.02)	(0.02)			

Note. Average mean absolute error (MAE) and coefficient of determination (R^2) displayed with standard deviation (SD) appearing in parentheses. Folds > ref. = percentage (%) of folds for which real models outperform dummy regressor in terms of coefficient of determination (R^2); Pure = pipeline without feature selection; FS = pipeline with feature selection; nr = no confound regression; Ridge(adj. λ): default values manually adjusted; nr-cr = age, sex & education regressed from target; cr-cr = age, sex & education regressed from target and features. Colour scheme: green = real model outperforms dummy in ≥ 80% of folds; orange = real model outperforms dummy in 50 - 80% of folds; red = real model outperforms dummy in ≤ 50% of folds.

Classification of extreme cognitive groups

Supplementary Table S38. Classification performance of extreme cognitive groups across feature sets and samples for global cognition.

		Unmatched				Matched			
		421		1621		421		1621	
		nr	cr	nr	cr	nr	cr	nr	cr
Pure	SVM	62.50%	59.41%	61.95%	57.19%	60.18%	61.79%	56.93%	58.88%
	(RBF)	(6.82%)	(6.39%)	(8.05%)	(7.78%)	(12.54%)	(11.22%)	(12.29%)	(11.72%)
	SVM	57.05%	50.52%	59.16%	50.67%	53.66%	54.52%	52.57%	57.05%
	(linear)	(7.37%)	(7.10%)	(7.55%)	(6.79%)	(13.30%)	(15.22%)	(13.34%)	(14.46%)
	SVM	59.42%	52.93%	59.42%	52.64%	53.84%	54.41%	55.21%	54.95%
	(poly)	(5.66%)	(4.78%)	(5.75%)	(4.54%)	(9.56%)	(10.68%)	(9.99%)	(12.13%)
	KNN	58.59%	54.76%	55.34%	55.68%	55.41%	53.84%	51.82%	53.86%
		(7.26%)	(7.53%)	(8.00%)	(8.03%)	(10.12%)	(11.84%)	(12.34%)	(11.00%)
	DT	54.64%	56.83%	55.04%	59.83%	56.36%	54.16%	55.20%	52.52%
		(8.79%)	(7.94%)	(7.57%)	(7.57%)	(12.57%)	(12.81%)	(11.46%)	(10.18%)
	NB	58.60%	54.67%	58.85%	55.78%	60.18%	58.27%	59.23%	59.55%
		(7.64%)	(8.41%)	(7.44%)	(8.29%)	(12.01%)	(12.87%)	(11.48%)	(10.92%)
	LDA	51.82%	51.38%	56.38%	52.85%	59.27%	52.88%	61.11%	56.95%
		(7.93%)	(7.07%)	(6.28%)	(8.08%)	(10.85%)	(13.65%)	(11.67%)	(14.15%)
FS	SVM	59.77%	57.45%	60.14%	57.91%	59.05%	59.07%	60.64%	58.95%
	(RBF)	(7.12%)	(6.66%)	(6.45%)	(6.72%)	(11.79%)	(12.84%)	(9.97%)	(11.20%)
	SVM	59.73%	52.04%	60.24%	50.55%	53.13%	57.73%	54.73%	54.23%
	(linear)	(7.65%)	(8.66%)	(7.36%)	(7.03%)	(14.62%)	(13.77%)	(13.76%)	(13.99%)
	SVM	57.90%	53.66%	57.76%	54.34%	54.39%	54.05%	53.84%	53.61%
	(poly)	(6.00%)	(5.77%)	(6.21%)	(7.79%)	(11.00%)	(11.65%)	(11.78%)	(12.05%)
	KNN	52.60%	52.37%	53.99%	56.76%	51.91%	53.23%	53.29%	51.73%
		(7.91%)	(7.34%)	(7.72%)	(7.46%)	(12.70%)	(10.83%)	(11.47%)	(11.62%)
	DT	53.98%	54.13%	54.58%	56.78%	54.45%	55.30%	51.73%	50.70%
		(6.72%)	(7.50%)	(7.67%)	(7.60%)	(11.59%)	(12.14%)	(12.56%)	(10.82%)
	NB	59.36%	53.00%	59.31%	53.14%	59.68%	58.11%	57.73%	59.07%
		(6.77%)	(8.28%)	(6.85%)	(9.15%)	(11.41%)	(10.72%)	(12.18%)	(11.24%)
	LDA	59.63%	52.35%	55.35%	49.70%	51.88%	53.11%	54.38%	57.52%
		(8.10%)	(8.51%)	(7.81%)	(7.04%)	(13.07%)	(13.82%)	(14.49%)	(12.44%)

Note. Mean balanced accuracies (BAC (%)) displayed with standard deviation (SD) appearing in parentheses. Pure = pipeline without feature selection; FS = univariate FS (ANOVA F-test) pipeline; nr = no confound regression; cr = age, sex & education regressed from features.

Supplementary Table S39. Classification performance of extreme cognitive groups across feature sets and samples for global cognition in comparison to dummy classifier.

		Unmatched				Matched			
		421		1621		421		1621	
		nr	cr	nr	cr	nr	cr	nr	cr
Pure	SVM (RBF)	98%	94%	92%	82%	78%	94%	74%	82%
	SVM (linear)	80%	46%	86%	48%	62%	58%	58%	60%
	SVM (poly)	96%	76%	96%	66%	66%	56%	68%	54%
	KNN	82%	72%	68%	76%	70%	64%	54%	50%
	DT	68%	78%	78%	92%	62%	60%	74%	58%
	NB	84%	68%	90%	72%	78%	74%	78%	72%
	LDA	60%	56%	78%	58%	74%	54%	82%	56%
FS	SVM (RBF)	90%	86%	90%	86%	74%	76%	74%	78%
	SVM (linear)	88%	58%	90%	40%	60%	64%	68%	64%
	SVM (poly)	88%	74%	86%	70%	56%	60%	62%	62%
	KNN	62%	60%	64%	86%	52%	52%	70%	58%
	DT	74%	74%	66%	84%	60%	62%	52%	54%
	NB	92%	64%	94%	64%	80%	72%	80%	76%
	LDA	88%	64%	74%	48%	54%	64%	62%	68%

Note. Percentage (%) of folds with higher accuracy in real model than dummy classifier. Pure = pipeline without feature selection; FS = univariate FS (ANOVA F-test) pipeline; nr = no confound regression; cr = age, sex & education regressed from features. Colour scheme: green = real model outperforms dummy in ≥ 80% of folds; orange = real model outperforms dummy in 50 - 80% of folds; red = real model outperforms dummy in ≤ 50% of folds.

Age classification and prediction

Supplementary Table S40. Results from classification of extreme age groups.

	Pure	folds > ref.	ANOVA F-test	folds > ref.	FS Reg.	folds > ref.	Hybrid	folds > ref.	HPO+FS	folds > ref.	HPO	folds > ref.
SVM (RBF)	79.53% (12.75%)	98%	72.73% (14.04%)	92%	80.87% (12.70%)	100%	71.67% (15.23%)	86%	72.73% (14.04%)	94%	81.57% (11.53%)	100%
SVM (linear)	85.13% (11.21%)	100%	71.40% (16.05%)	90%	77.37% (12.67%)	96%	66.60% (16.47%)	76%	71.40% (16.05%)	84%	83.13% (12.07%)	100%
SVM (poly)	69.40% (12.92%)	94%	74.03% (14.78%)	88%	72.67% (13.98%)	86%	69.80% (13.41%)	94%	74.03% (14.78%)	92%	67.67% (13.65%)	84%
KNN	73.00% (14.76%)	94%	70.57% (14.23%)	92%	71.50% (15.59%)	92%	67.77% (15.92%)	82%	70.57% (14.23%)	94%	74.10% (15.54%)	92%
DT	68.83% (14.23%)	84%	65.97% (13.20%)	90%	58.77% (15.24%)	86%	60.70% (13.88%)	74%	65.97% (13.20%)	76%	67.30% (16.83%)	84%
NB	74.27% (14.42%)	92%	72.73% (14.04%)	78%	78.70% (15.21%)	92%	68.90% (18.40%)	78%	-		-	
LDA	80.60% (12.32%)	100%	71.40% (16.05%)	76%	73.43% (12.55%)	96%	68.87% (15.73%)	86%	-		-	

Note. Mean balanced accuracies (BAC (%)) displayed with standard deviation (SD) appearing in parentheses. Classification results across different FS and HPO pipelines: no FS (Pure), ANOVA F-test filter, FS with regularization (FS Regularization), filter and wrapper combined (Hybrid), hyperparameter optimization and ANOVA F-test filter (HPO + FS), hyperparameter optimization without additional FS (HPO). Folds > ref. = percentage (%) of folds for which real models outperform dummy classifier in terms of balanced accuracy (BAC). Colour scheme: green = real model outperforms dummy in ≥ 80% of folds; orange = real model outperforms dummy in 50 - 80% of folds; red = real model outperforms dummy in ≤ 50% of folds.

Supplementary Table S41. Age prediction results in different samples.

		Extreme Groups				Sample 813				Whole Sample			
		Feature Set 421		Feature Set 1621		Feature Set 421		Feature Set 1621		Feature Set 421		Feature Set 1621	
		MAE	R^2	MAE	R^2	MAE	R^2	MAE	R^2	MAE	R^2	MAE	R^2
Pure	SVR	25.57	-0.97	25.85	-1.03	5.34	0.09	5.36	0.09	8.54	0.16	8.55	0.16
	(RBF)	(3.38)	(1.05)	(3.39)	(1.06)	(0.40)	(0.06)	(0.40)	(0.05)	(0.65)	(0.04)	(0.65)	(0.04)
	RVR	16.43	0.18	17.69	0.08	5.27	0.07	5.17	0.11	7.78	0.36	7.54	0.41
		(3.56)	(0.53)	(3.17)	(0.48)	(0.42)	(0.10)	(0.40)	(0.10)	(0.58)	(0.08)	(0.52)	(0.07)
	Elastic	15.59	0.27	15.10	0.30	5.34	0.10	5.28	0.11	7.99	0.31	7.71	0.36
	Net	(3.29)	(0.40)	(3.05)	(0.33)	(0.40)	(0.05)	(0.40)	(0.06)	(0.57)	(0.05)	(0.57)	(0.05)
	Lasso	16.71	0.14	15.86	0.23	5.57	0.04	5.54	0.04	8.28	0.26	8.07	0.30
		(3.92)	(0.49)	(3.38)	(0.35)	(0.41)	(0.03)	(0.41)	(0.03)	(2.02)	(0.04)	(0.58)	(0.04)
	Ridge	15.81	0.17	14.46	0.32	6.77	-0.65	9.15	-2.04	9.36	0.11	16.03	-1.58
	($\lambda = 1$)	(3.30)	(0.43)	(2.82)	(0.34)	(0.65)	(0.31)	(0.59)	(0.46)	(0.60)	(0.13)	(0.94)	(0.38)
	Ridge	15.63	0.30	14.48	0.36	5.07	0.12	5.53	-0.06	7.65	0.37	8.21	0.31
	($adj. \lambda$)	(2.79)	(0.35)	(2.64)	(0.32)	(0.43)	(0.12)	(0.41)	(0.14)	(0.57)	(0.07)	(0.51)	(0.10)
FS	SVR	17.30	0.01	16.14	0.17	5.42	0.06	5.31	0.08	8.78	0.15	8.20	0.26
		(4.49)	(0.52)	(3.89)	(0.40)	(0.40)	(0.08)	(0.40)	(0.09)	(0.65)	(0.06)	(0.67)	(0.07)
	RVR	17.48	0.04	15.30	0.20	5.44	0.04	5.44	0.02	8.85	0.16	8.48	0.23
		(3.86)	(0.49)	(3.41)	(0.37)	(0.42)	(0.09)	(0.40)	(0.10)	(0.70)	(0.09)	(0.69)	(0.10)
	Elastic	15.73	0.27	14.85	0.33	5.09	0.15	5.02	0.18	7.57	0.38	7.37	0.42
	Net	(3.07)	(0.37)	(2.64)	(0.32)	(0.41)	(0.10)	(0.39)	(0.09)	(0.56)	(0.06)	(0.51)	(0.06)
	Lasso	17.64	0.06	16.59	0.18	5.09	0.15	5.23	0.11	7.68	0.37	7.80	0.36
		(3.83)	(0.52)	(3.11)	(0.37)	(0.41)	(0.10)	(0.40)	(0.09)	(0.52)	(0.07)	(0.53)	(0.07)
	Ridge	18.85	0.02	16.40	0.21	5.36	0.08	5.29	0.10	8.81	0.17	8.16	0.29
		(3.18)	(0.40)	(3.08)	(0.35)	(0.41)	(0.07)	(0.41)	(0.08)	(0.65)	(0.06)	(0.64)	(0.07)

Note. Average mean absolute error (MAE) and coefficient of determination (R^2) displayed with standard deviation (SD) appearing in parentheses**.** Pure = pipeline without feature selection; FS = pipeline with feature selection; Ridge(adj. λ): default values manually adjusted.

Supplementary Table S42. Age prediction results in different samples in comparison to dummy regressor.

		Extreme Groups		Sample 813		Whole Sample	
		421	1621	421	1621	421	1621
Pure	SVR (RBF)	10%	10%	98%	98%	100%	100%
	RVR	88%	78%	90%	92%	100%	100%
	Elastic Net	92%	94%	96%	96%	100%	100%
	Lasso	84%	92%	96%	96%	100%	100%
	Ridge ($\lambda = 1$)	82%	98%	8%	0%	86%	0%
	Ridge ($adj. \lambda$)	98%	100%	90%	56%	100%	100%
FS	SVR	68%	84%	90%	88%	98%	100%
	RVR	72%	88%	80%	76%	98%	100%
	Elastic Net	96%	100%	92%	94%	100%	100%
	Lasso	78%	88%	94%	92%	100%	100%
	Ridge	68%	96%	94%	88%	100%	100%

Note. Percentage (%) of folds for which real models outperform dummy regressor in terms of coefficient of determination (R^2). Pure = pipeline without feature selection; FS = pipeline with feature selection; Ridge(adj. λ): default values manually adjusted. Colour scheme: green = real model outperforms dummy in ≥ 80% of folds; orange = real model outperforms dummy in 50 - 80% of folds; red = real model outperforms dummy in ≤ 50% of folds.

References

Aschenbrenner, S., Tucha, O., & Lange, K. W. (2000). *RWT: Regensburger Wortflüssigkeits-Test*. Hogrefe.

Bäumler, G. (1985). *Farbe-Wort-Interferenztest nach JR Stroop*. Hogrefe, Verlag für Psychologie.

Benton, A., Sivan, A. B., Spreen, O., & Steck, P. (2009). *Der Benton-Test*. Huber.

Bishop, C. M. (2006). *Pattern recognition and machine learning*. Springer.

Caspers, S., Moebus, S., Lux, S., Pundt, N., Schütz, H., Mühleisen, T. W., Gras, V., Eickhoff, S. B., Romanzetti, S., Stöcker, T., Stirnberg, R., Kirlangic, M. E., Minnerop, M., Pieperhoff, P., Mödder, U., Das, S., Evans, A. C., Jöckel, K.-H., Erbel, R., … Amunts, K. (2014). Studying variability in human brain aging in a population-based German cohort- rationale and design of 1000BRAINS.*Frontiers in Aging Neuroscience, 6*. https://doi.org/10.3389/fnagi.2014.00149

Chen, X., Kar, S., & Ralescu, D. A. (2012). Cross-entropy measure of uncertain variables. *Information Sciences*, *201*, 53–60. https://doi.org/10.1016/j.ins.2012.02.049

Della Sala, S., Gray, C., Baddeley, A. D., & Wilson, L. (1997).*The visual patterns test: A test of short-term visual recall.* Thames Valley Test Company.

Drucker, H., Burges, C. J. C., Kaufman, L., Smola, A. J., & Vapnik, V. (1997). *Support Vector Regression Machines: Vol. ADVANCES IN NEURAL INFORMATION PROCESSING SYSTEMS 9*. MIT Press.

Erickson, B. J., Korfiatis, P., Akkus, Z., & Kline, T. L. (2017). Machine Learning for Medical Imaging. *RadioGraphics*, *37*(2), 505–515. https://doi.org/10.1148/rg.2017160130

Fisher, R. A. (1936). THE USE OF MULTIPLE MEASUREMENTS IN TAXONOMIC PROBLEMS. *Annals of Eugenics*, *7*(2), 179–188. https://doi.org/10.1111/j.1469-1809.1936.tb02137.x

Foody, G. M. (1995). Cross-entropy for the evaluation of the accuracy of a fuzzy land cover classification with fuzzy ground data. *ISPRS Journal of Photogrammetry and Remote Sensing*, *50*(5), 2–12. https://doi.org/10.1016/0924-2716(95)90116-V

Gaser, C., Franke, K., Klöppel, S., Koutsouleris, N., Sauer, H., & Alzheimer's Disease Neuroimaging Initiative. (2013). BrainAGE in Mild Cognitive Impaired Patients: Predicting the Conversion to Alzheimer's Disease. *PLoS ONE*, *8*(6), e67346. https://doi.org/10.1371/journal.pone.0067346

Gatterer, G. (2008). *Alters-Konzentrations-Test* (2nd ed). Hogrefe.

Hastie, T., Tibshirani, R., & Friedman, J. H. (2009). *The elements of statistical learning: Data mining, inference, and prediction* (2nd ed). Springer.

Hoerl, A. E., & Kennard, R. W. (1970). Ridge Regression: Biased Estimation for Nonorthogonal Problems. *Technometrics*, *12*(1), 55–67. https://doi.org/10.1080/00401706.1970.10488634

Jockwitz, C., Caspers, S., Lux, S., Eickhoff, S. B., Jütten, K., Lenzen, S., Moebus, S., Pundt, N., Reid, A., Hoffstaedter, F., Jöckel, K.-H., Erbel, R., Cichon, S., Nöthen, M. M., Shah, N. J., Zilles, K., & Amunts, K. (2017). Influence of age and cognitive performance on resting-state brain networks of older adults in a population-based cohort. *Cortex*, *89*, 28–44. https://doi.org/10.1016/j.cortex.2017.01.008

Loh, W. (2011). Classification and regression trees. *WIREs Data Mining and Knowledge Discovery*, *1*(1), 14–23. https://doi.org/10.1002/widm.8

Lux, S., Hartje, W., Reich, C., & Nagel, C. (2012). *VGT: Verbaler Gedächtnistest: Bielefelder Kategorielle Wortlisten*. Verlag Hans Huber.

McLachlan, G. J. (2004). *Discriminant analysis and statistical pattern recognition*. Wiley.

Mohri, M., Rostamizadeh, A., & Talwalkar, A. (2018). *Foundations of machine learning* (2nd ed). The MIT Press.

Morris, J. C., Heyman, A., Mohs, R. C., Hughes, J. P., van Belle, G., Fillenbaum, G., Mellits, E. D., & Clark, C. (1989). The Consortium to Establish a Registry for Alzheimer's Disease (CERAD). Part I. Clinical and neuropsychological assessment of Alzheimer's disease. *Neurology*, *39*(9), 1159–1159. https://doi.org/10.1212/WNL.39.9.1159

Oswald, W. D., & Fleischmann, U. M. (1997). *Das Nürnberger-Alters-Inventar (NAI)*. Hogrefe.

Pereira, F., Mitchell, T., & Botvinick, M. (2009). Machine learning classifiers and fMRI: A tutorial overview. *NeuroImage*, *45*(1), S199–S209. https://doi.org/10.1016/j.neuroimage.2008.11.007

Quinlan, J. R. (1987). DECISION TREES AS PROBABILISTIC CLASSIFIERS. In *Proceedings of the Fourth International Workshop on MACHINE LEARNING* (pp. 31–37). Elsevier. https://doi.org/10.1016/B978-0-934613-41-5.50007-6

Regard, M., Strauss, E., & Knapp, P. (1982). Children's Production on Verbal and Non-Verbal Fluency Tasks. *Perceptual and Motor Skills*, *55*(3), 839–844. https://doi.org/10.2466/pms.1982.55.3.839

Schelling, D. (1997). *Block-tapping-test*. Swets Test Service GmbH.

Schmidt, K. H., & Metzler, P. (1992). *Wortschatztest (WST)*. Beltz Test GmbH.

Schölkopf, B., & Smola, A. J. (2002). *Learning with kernels: Support vector machines, regularization, optimization, and beyond*. MIT Press.

Schölkopf, B., Tsuda, K., & Vert, J.-P. (Eds.). (2004). *Kernel methods in computational biology*. MIT Press.

Serra, A., Galdi, P., & Tagliaferri, R. (2018). Machine learning for bioinformatics and neuroimaging. *WIREs Data Mining and Knowledge Discovery*, *8*(5). https://doi.org/10.1002/widm.1248

Stroop, J. R. (1935). Studies of interference in serial verbal reactions. *Journal of Experimental Psychology*, *18*(6), 643–662. https://doi.org/10.1037/h0054651

Sturm, W., Willmes, K., & Horn, W. (1993). *Leistungsprüfsystem für 50-90jährige (LPS 50+)*. Hogrefe Verlag für Psychologie.

Tibshirani, R. (1996). Regression Shrinkage and Selection Via the Lasso. *Journal of the Royal Statistical Society: Series B (Methodological)*, *58*(1), 267–288. https://doi.org/10.1111/j.2517-6161.1996.tb02080.x

Tipping, M. E. (2001). Sparse Bayesian Learning and the Relevance Vector Machine. *Journal of Machine Learning Research*,*1*((Jun)), 211–244.

Vapnik, V. N. (1995). *The nature of statistical learning theory*. Springer.

Zou, H., & Hastie, T. (2005). Regularization and variable selection via the elastic net. *Journal of the Royal Statistical Society: Series B (Statistical Methodology)*, *67*(2), 301–320.
